# Advances in Understanding the Karyotype Evolution of Tetrapulmonata and Two Other Arachnid Taxa, Ricinulei and Solifugae

**DOI:** 10.3390/genes16020207

**Published:** 2025-02-08

**Authors:** Jiří Král, Alexandr Sember, Klára Divišová, Tereza Kořínková, Azucena C. Reyes Lerma, Ivalú M. Ávila Herrera, Martin Forman, František Šťáhlavský, Jana Musilová, Sabrina Torres Kalme, José G. Palacios Vargas, Magda Zrzavá, Iva Vrbová, Jairo A. Moreno-González, Paula E. Cushing, Alexander V. Gromov, Štěpánka Šebestiánová, Vendula Bohlen Šlechtová, Lorenzo Prendini, Tharina L. Bird

**Affiliations:** 1 Department of Genetics and Microbiology, Faculty of Science, Charles University, Viničná 5, 128 44 Prague, Czech Republic; dweep2@seznam.cz (K.D.); areyes.lerma@gmail.com (A.C.R.L.); ivalu.a@gmail.com (I.M.Á.H.); formivelkejpan@seznam.cz (M.F.); jana-musilova@seznam.cz (J.M.); sabrina-tk2009@hotmail.com (S.T.K.); 2 Institute of Animal Physiology and Genetics AS CR, Rumburská 89, 277 21 Liběchov, Czech Republic; sember@iapg.cas.cz (A.S.); v.slechtova@iapg.cas.cz (V.B.Š.); 3 Na Perštýně 2, 110 00 Prague, Czech Republic; te.korinkova@gmail.com; 4 Department of Zoology, Faculty of Science, Charles University, Viničná 7, 128 44 Prague, Czech Republic; frantisek.stahlavsky@natur.cuni.cz; 5 Division of Crop Genetics and Breeding, Crop Research Institute, Drnovská 507/73, 161 00 Prague, Czech Republic; 6 Departamento de Ecología y Recursos Naturales, Facultad de Ciencias, Universidad Nacional Autónoma de México, México City 04510, Mexico; troglolaphysa@hotmail.com; 7 Faculty of Science, University of South Bohemia, Branišovská 1760, 370 05 České Budějovice, Czech Republic; maggie@prf.jcu.cz; 8 Department of Molecular Biology and Genetics, Institute of Entomology, Biology Centre CAS, Branišovská 31, 370 05 České Budějovice, Czech Republic; 9 Department of Molecular Cytogenetics, Institute of Plant Molecular Biology, Biology Centre CAS, Branišovská 31, 370 05 České Budějovice, Czech Republic; ifukova@umbr.cas.cz; 10 Division of Invertebrate Zoology, American Museum of Natural History, Central Park West at 79th Street, New York, NY 10024, USA; jmorenogonzalez@amnh.org (J.A.M.-G.); lorenzo@amnh.com (L.P.); 11 Department of Zoology, Denver Museum of Nature and Science, 2001 Colorado Blvd., Denver, CO 80205, USA; paula.cushing@dmns.org; 12 Senckenberg Research Institute, Arachnology, Mertonstrasse 17-21, 60325 Frankfurt, Germany; alexander_gromov@yahoo.com; 13 Institute of Physiotherapy and Selected Medical Disciplines, Faculty of Health and Social Sciences, University of South Bohemia, J. Boreckého 1167/27, 370 11 České Budějovice, Czech Republic; stebsebe@seznam.cz; 14 Ditsong National Museum of Natural History, 432 Paul Kruger Street, Pretoria 0001, South Africa; tharina@ditsong.org.za; 15 Department of Zoology and Entomology, University of Pretoria, Pretoria 0083, South Africa

**Keywords:** heterochromatin, holocentric, nucleolus organizer region, polyploidy, sex chromosome, solifuge, somatic pairing, Ricinulei, spider, telomere

## Abstract

Background/Objectives: Arachnids are a megadiverse arthropod group. The present study investigated the chromosomes of pedipalpid tetrapulmonates (orders Amblypygi, Thelyphonida, Schizomida) and two arachnid orders of uncertain phylogenetic placement, Ricinulei and Solifugae, to reconstruct their karyotype evolution. Except for amblypygids, the cytogenetics of these arachnid orders was almost unknown prior to the present study. Methods: Chromosomes were investigated using methods of standard (Giemsa-stained preparations, banding techniques) and molecular cytogenetics (fluorescence in situ hybridization, comparative genomic hybridization). Results and Conclusions: New data for 38 species, combined with previously published data, suggest that ancestral arachnids possessed low to moderate 2n (22–40), monocentric chromosomes, one nucleolus organizer region (NOR), low levels of heterochromatin and recombinations, and no or homomorphic sex chromosomes. Karyotypes of Pedipalpi and Solifugae diversified via centric fusions, pericentric inversions, and changes in the pattern of NORs and, in solifuges, also through tandem fusions. Some solifuges display an enormous amount of constitutive heterochromatin and high NOR number. It is hypothesized that the common ancestor of amblypygids, thelyphonids, and spiders exhibited a homomorphic XY system, and that telomeric heterochromatin and NORs were involved in the evolution of amblypygid sex chromosomes. The new findings support the Cephalosomata clade (acariforms, palpigrades, and solifuges). Hypotheses concerning the origin of acariform holocentric chromosomes are presented. Unlike current phylogenetic hypotheses, the results suggest a sister relationship between Schizomida and a clade comprising other tetrapulmonates as well as a polyploidization in the common ancestor of the clade comprising Araneae, Amblypygi, and Thelyphonida.

## 1. Introduction

The class Arachnida is an ancient and megadiverse arthropod lineage. Extant arachnids comprise two acarine orders (the mites)—Acariformes and Parasitiformes (e.g., [1]), and ten non-acarine orders, namely Amblypygi (whipspiders), Araneae (spiders), Opiliones (harvestmen), Palpigradi (microwhipscorpions), Pseudoscorpiones (pseudoscorpions), Ricinulei (hooded tick spiders), Schizomida (short-tailed whipscorpions), Scorpiones (scorpions), Solifugae (solifuges), and Thelyphonida (whipscorpions) [2]. Higher level arachnid phylogenies remain controversial, despite the use of fossil [3] and molecular datasets (e.g., [1,4,5,6,7,8,9,10,11,12]). This may largely be attributed to the ancient age of arachnids and their rapid radiation [7]. Cytogenetic markers could provide additional data for testing hypotheses concerning arachnid phylogeny and evolution. As hereditary elements of the genome, chromosomes act as independent mutational units and are thus suitable as characters. Cytogenetics may provide chromosome data that are difficult or even impossible to obtain by genome assembly (e.g., centromere position, sex chromosome system, location and base composition of blocks of constitutive heterochromatin, course of meiosis) [13]. This layer of information is, however, largely missing for many arachnid lineages. Most studies investigated spiders [14], acariform and parasitiform mites [15], harvestmen [16], scorpions [17], and pseudoscorpions [18]. The other orders remain mostly or entirely unknown cytogenetically.

The present study aimed to reconstruct the karyotype evolution of the tetrapulmonate arachnids (Tetrapulmonata). This clade includes spiders, and three tropical to subtropical orders namely Amblypygi, Schizomida, and Thelyphonida [19]. Spiders are a highly speciose order consisting of three major lineages (suborder Mesothelae and two infraorders, Mygalomorphae and Araneomorphae) [20], 134 families, and 52,246 species [21], with most species belonging to Araneomorphae. Amblypygids comprise two primary lineages, namely the Paleoamblypygi, represented by a single family containing two relictual genera [22], and Euamblypygi comprising three families, 16 genera, and 277 species [23,24]. The hypothesis concerning the intraordinal phylogeny of amblypygids is based on molecular data. Euamblypygi comprise two clades, Charontidae (including the former family Charinidae) and Phrynoidea (comprising families Phrynichidae and Phrynidae) [24]. Schizomids include two families, 71 genera, and 376 species [25]. Extant thelyphonids comprise a single family containing four subfamilies [19], 16 genera, and 126 species [26]. The intraordinal phylogeny of schizomids and thelyphonids was recently analyzed using molecular data [27].

In contrast to the other supraordinal arachnid clades, tetrapulmonates are considered a monophyletic group with strong support (e.g., [5,28]). The interordinal relationships among other tetrapulmonate orders remain contested, however. Although amblypygids were initially considered to be the sister taxon of spiders [4,29,30,31], the Pedipalpi hypothesis, according to which amblypygids, schizomids, and thelyphonids are monophyletic, is more generally accepted by recent authors. According to this hypothesis, Amblypygi is sister to Uropygi, a monophyletic group comprising schizomids and thelyphonids [5,6,9,10,11,32,33,34]. Pedipalpi was not recovered by Pepato et al. [28] based on molecular data alone but was recovered by their combined analysis of morphological and molecular data [28]. Most recent analyses consistently recovered Uropygi [5,6,27,29,32,33]. However, mitogenomic data suggest Schizomida is the sister group of a clade comprising Amblypygi and Thelyphonida or the sister group of Amblypygi [35].

The phylogenetic placement of tetrapulmonates relative to other arachnids has long been debated. Tetrapulmonates were variously grouped with Palpigradi [4], the extinct paleozoic order Trigonotarbida [36], or Ricinulei [28,31]. Ricinulei, a relictual order that has existed since the Upper Carboniferous [37], comprises a single extant family including three genera and 103 species [38]. Molecular datasets have been used to analyze the internal phylogeny of ricinuleids [39,40,41,42]. Recent phylogenomic studies consistently supported the Arachnopulmonata hypothesis, according to which Tetrapulmonata is the sister group of Panscorpiones (Scorpiones and Pseudoscorpiones). This hypothesis was also supported by data suggesting genome duplication in the arachnopulmonate ancestor [8,43,44,45,46]. The phylogenetic position of arachnopulmonates relative to other arachnids remains unresolved, despite the use of genome-scale datasets [46]. Based on these datasets, Arachnopulmonata is placed, among others, with a clade comprising Solifugae, Ricinulei, and Xiphosura [10], a clade comprising Ricinulei and Xiphosura [47] or a clade comprising Ricinulei, Opiliones, Solifuges, Acariformes, and Parasitiformes [9].

Some of the above-mentioned studies placed solifuges among the relatives of arachnopulmonates [9,10]. The relatively diverse order Solifugae, comprising 15 families and more than 1200 species [48], was traditionally considered the sister group of pseudoscorpions [19], forming Haplocnemata [33]. There is, however, growing evidence for a clade comprising Solifugae and Acariformes, the Poecilophysidea clade [28], initially based on ultrastructural and morphological features (see [49]), and later supported by three molecular phylogenetic analyses [28,50,51]. Two of these studies suggested a close relationship between solifuges, acariform mites, and palpigrades, forming the Cephalosomata clade [28,51]. The intraordinal evolutionary relationships of solifuges were recently tested using ultraconserved elements [52]. According to this study, solifuges comprise two major clades, Boreosolifugae comprising families Eremobatidae, Galeodidae, Karschiidae, Rhagodidae, and Australosolifugae containing the remaining families.

In terms of cytogenetics, spiders are the most studied order of tetrapulmonates. The chromosome diversity of spiders mirrors their enormous diversity. Diploid numbers vary from 5 to 152 [53]. In some groups, monoarmed (acrocentric and subtelocentric) chromosomes predominate whereas in others, biarmed (metacentric and submetacentric) chromosomes predominate. The chromosomes of superfamily Dysderoidea are holocentric. Spiders are characterized by complex sex chromosome systems, which encompass a varied number of different X chromosomes (up to 13) and, in some clades, also a peculiar Y microchromosome, which pair without chiasmata during male meiosis [54,55]. Furthermore, an additional largely undifferentiated pair (or even two) of sex chromosomes is probably part of the spider sex chromosome system [54,55,56,57,58]. In many spider taxa, the number of chromosomes was reduced by fusions [54]. The number of nucleolar organizer regions (NORs) ranges widely in spiders, from one to ten [59].

The cytogenetics of Pedipalpi is poorly known. To date, karyotypes are available for 17 amblypygids (members of the families Charontidae and Phrynidae [60,61,62]), one thelyphonid (*Thelyphonus sepiaris*, also reported as *Thelyphonus indicus*) [63,64,65]), and two schizomids [66]). Diploid numbers were reported for two other amblypygids [63,67] and another thelyphonid [68]. Available data suggest that, in the amblypygid families studied, there was a reduction in the number of chromosomes during evolution, as in spiders [60,62].

In order to understand the karyotype evolution of tetrapulmonates, and thus contribute towards a greater understanding of arachnid evolution, a range of cytogenetic characters was analyzed, namely diploid chromosome number (2n), chromosome morphology, constitutive heterochromatin (CH), NORs, telomeric repeats, sex chromosomes, and meiotic features. The present study includes 38 species (Table S1), representing a cross-section of all Pedipalpi clades and two orders, which might be related to Arachnopulmonata, Ricinulei and Solifugae. Except for the amblypygids *Damon medius* and *Paraphrynus mexicanus*, and the solifuge *Rhagodes* sp. [60,67], the cytogenetics of species selected was analyzed for the first time. Information about the cytogenetics of Pedipalpi could be important for reconstructing not only the ancestral tetrapulmonate karyotype but also the ancestral spider karyotype, thus elucidating the origin of unusual sex chromosomes in spiders. Except for the diploid number of one solifuge species [67], the cytogenetics of Ricinulei and Solifugae was previously unknown. Fundamental traits of karyotype evolution in cephalosomate and tetrapulmonate arachnids are reconstructed for the first time, using new data presented herein. These data may also contribute characters for phylogenetic analyses concerning cephalosomate and arachnopulmonate arachnids.

## 2. Material and Methods

### 2.1. Material Examined and Taxonomic Determination

Live specimens of different taxa of Pedipalpi (Amblypygi, Schizomida, and Thelyphonida), Solifugae, and Ricinulei, were obtained and housed in plastic terraria at appropriate temperature and humidity. Amblypygids and thelyphonids were reared to ontogenetic stages suitable for study, but ricinuleids and most schizomids did not molt in captivity, and solifuges did not survive more than a few weeks. Specimen provenance data are presented in Table S1.

Material examined is deposited at the Department of Genetics and Microbiology, Faculty of Science, Charles University, Prague, the American Museum of Natural History, New York, NY, USA, and the Denver Museum of Nature and Science, Denver, CO, USA. Undescribed taxa or immatures which lack diagnostic characters could not be determined to species or even genus. Some small specimens (e.g., schizomids) were damaged by dissection and their exact determination was consequently impossible.

### 2.2. Optimal Ontogenetic Stages and Tissues for Chromosome Preparation

A high frequency of cell division was detected in the gonads and intestines of most or all life stages in the taxa studied. Solifuge postembryos and ventral glands of amblypygid males provided additional sources of dividing cells. Preparations from small juveniles were obtained from the entire content of the opisthosoma. Male gonads were optimal tissues, also providing information about meiotic chromosome behavior. Complete sequences of male meiosis were found in the testes of subadult (amblypygids, thelyphonids) and/or adult males (amblypygids, thelyphonids, schizomids, ricinuleids). In subadult male schizomids and some subadult male amblypygids and thelyphonids, meiotic cells consisted of early prophase I (i.e., leptotene–pachytene) spermatocytes only. Adult male amblypygids and thelyphonids produced meiotic cells continually throughout the year. In contrast, spermatocytes were only detected in some adult male ricinuleids and schizomids, suggesting meiosis was restricted to a relatively short period. No meiotic cells were found in juvenile and adult male solifuges, and a very low frequency of cell division in adult solifuges. Meiotic cells were also found in the ovaries of juvenile females in all taxa studied except ricinuleids. However, only prophase nuclei of the first meiotic division were detected. Unlike in the other arachnids studied, no gonads could be detected in juvenile ricinuleids and their opisthosomas contained only occasional mitotic plates.

### 2.3. Preparation of Chromosomes and Analysis of Constitutive Heterochromatin

Chromosome preparations were obtained by a spreading technique using a histological plate [69,70]. Large-volume tissues were fixed three times (6, 10, and 30 min). Slides for molecular cytogenetics were dehydrated in an ethanol series (70, 80, and 96%; 1 min each) and stored at −75 °C. Constitutive heterochromatin was detected by C-banding, using a standard protocol [71]. To preserve the chromatin for C-banding, the temperature of the histological plate was reduced to 35 °C. Among the amblypygids and thelyphonids, the temperature of the Ba(OH)_2_ solution (Lach-Ner, Neratovice, Czech Republic) was reduced to 40 °C in order to preserve the chromatin. Fluorescence banding was performed according to Sola et al. [72] with some modifications [71]. During this procedure, chromosomes were stained with the AT-specific fluorochrome 4′,6-diamidino-2-phenolindole (DAPI) (Sigma-Aldrich, St. Louis, MO, USA).

### 2.4. Detection of NORs and Telomere Repeats by Fluorescence In Situ Hybridization (FISH)

NORs were detected using an 18S rDNA probe amplified from the genome of the spider *Dysdera erythrina* (Dysderidae) and labelled with biotinylated dATP using the Bionick Labeling System nick translation kit (Invitrogen, Life Technologies, San Diego, CA, USA) (see [59,73] for details). The probe for detection of the arthropod telomeric motif (TTAGG)_n_ was generated by non-template PCR with primers 5′-TAGGTTAGGTTAGGTTAGGT-3′ and 5′-CTAACCTAACCTAACCTAAC-3′ (Generi Biotech, Hradec Králové, Czech Republic) using Taq HS DNA polymerase (GibcoBRL, Life Technologies Inc., Karlsruhe, Germany), following Sahara et al. [74]. The resulting fragments, with an average length of 1 kbp, were labelled with biotinylated dATP (see above for kit specification).

The protocol of Sahara et al. [74], with some modifications, was followed for FISH. Where necessary, slides were exposed to 65 °C (4 h) to improve adherence of chromosomes to the surface of the slides and then treated with proteinase K (Sigma-Aldrich) in 1× PBS (1 μg/mL, 5 min, 37 °C) to remove cytoplasmatic residues. Preparations were treated with RNase A (Biotech, Prague, Czech Republic) in 2× SSC (200 μg/mL, 1 h, 37 °C) to prevent probe hybridization with RNA. Chromosome DNA was denatured in 70% formamide (Sigma-Aldrich) in 2× SSC (3 min 30 s, 68 °C) and immediately dehydrated in ethanol series (70% ethanol pre-cooled to −20 °C, followed by 80% and 96% ethanol at RT, 1 min each). The probe cocktail for one slide (10 μL) contained 50 ng of probe, 25 μg of sonicated salmon sperm (Sigma-Aldrich), 50% deionized formamide, 10% dextran sulphate (Serva Electrophoresis GmbH, Heidelberg, Germany), and 2× SSC. The probe was denatured at 90 °C (5 min). Hybridization was performed overnight (37 °C, moist chamber). Post-hybridization washes and detection of probe signals [Cy3-conjugated streptavidin (Jackson ImmunoResearch Laboratories, West Grove, PA, USA) followed by one round of signal amplification with biotinylated anti-streptavidin (Vector Laboratories, Newark, NJ, USA) and Cy3-conjugated streptavidin] were carried out as described in [74]. Chromosomes were counterstained with 0.5 μg/mL DAPI and mounted in DABCO-based antifade (see [75] for composition).

### 2.5. Detection of Sex Chromosomes by Comparative Genomic Hybridization (CGH)

Comparative genomic hybridization is an excellent method to determine homomorphic sex chromosomes as well as for gross-scale delimitation of sex-specific regions in sex chromosomes [58,76]. In selected species, genomic DNA was isolated separately from males and females by standard phenol-chloroform-isoamylalcohol extraction [77]. Labelling of DNA was carried out by nick translation using Nick Translation Mix (Roche Diagnostics, Mannheim, Germany). Male DNA was labelled with Cy3-dUTP (GE Healthcare, Milwaukee, USA) and female DNA with Spectrum Green-dUTP (Vysis, Downers Grove, IL, USA). To reduce the hybridization of shared abundant repetitive DNA, unlabelled female genomic DNA was used as species-specific competitive DNA, fragmented by sonication (Sonopuls HD 2070, Bandelin Electric, Berlin, Germany) with two cycles of five pulses at 70% power, 10 s each. The sonication procedure produced fragments 100–200 bp long. Comparative genomic hybridization followed the procedure of Traut et al. [78]. The slide pretreatment as well as the slide and probe denaturation were conducted as described above for FISH. The hybridization mixture for one slide contained 1 µg of labelled male DNA, 1 or 2 µg of labelled female DNA, 10 µg of unlabelled female DNA (specific competitor), and 25 µg of sonicated salmon sperm DNA (Sigma-Aldrich) (non-specific competitor). The cocktail of *Charon* and *Typopeltis* contained more labelled male (5 µg) and female (10 µg) DNA. Hybridization was performed for three days in a humid chamber (37 °C). A stringent wash [0.1× SSC containing 1% Triton X-100 (Sigma-Aldrich), 5 min, 62 °C] was followed by DAPI counterstaining and mounting in DABCO antifade as described above.

### 2.6. Analysis of Preparations and Images

Standard and C-banded preparations were observed using a BX 50 microscope (Olympus, Tokyo, Japan), and images taken with a CCD camera DP 71 using the CellD program (Olympus). Fluorescent banding was evaluated in a Provis AX70 Olympus microscope, and images taken with a CCD camera DP30W using Acquisition Software (DP Controller version 2.1.1.183) (Olympus). FISH and CGH slides were inspected with an IX81 microscope (Olympus). Black-and-white images were taken separately for each fluorochrome using a CCD camera (ORCA C4742-80-12AG, Hammatsu, Japan). Images were pseudocoloured (light blue for DAPI, red for Cy3, and light green for SpectrumGreen) and superimposed with Cell^R software version 2.0 (Olympus Soft Imaging Solutions, Münster, Germany).

Four metaphase plates per individual and method were used to evaluate the morphology and size of chromosomes. Relative chromosome lengths were calculated as a percentage of the total chromosome length (% of TCL) of the diploid set. Classification of chromosome morphology was based on the position of the centromere according to [79]. Some chromosome pairs exhibited a transitional morphology between two types, which was denoted as morphology a/morphology b. Centromeres were difficult to detect in mitotic chromosomes due to indistinct primary constrictions, especially among most amblypygids and thelyphonids. In some species, centromeres of mitotic chromosomes could be visualized by C-banding as centromeric CH and/or as DAPI-positive areas (probably due to high AT base-pair content) after counterstaining FISH preparations with DAPI. Metaphase II was also informative for determining the position of the centromere because sister chromatids remain connected only at the centromere during this phase. In this case, plates containing both sister cells were used except for *Cryptocellus narino*, *Ricinoides olounoua*, and *Uroproctus assamensis*. Only plates containing one of the two sister cells were available for these species. During karyotype analysis, it was also determined whether the chromosome pairs of the species are predominantly biarmed or monoarmed. Proportion of biarmed and monoarmed pairs was considered approximately the same if the number of biarmed and the monoarmed pairs was the same or if there were one or two more pairs of one type [pairs with a transitional morphology between biarmed and monoarmed (sm/st) were not considered].

Chromosomes were measured using IMAGE TOOL 3.0 (UTHSCSA, San Antonio, TX, USA) or IMAGE J software versions 1.47 to 1.54 (National Institutes of Health, Bethesda, MD, USA). The following terms were used to indicate the position of a structure in a chromosome: terminal (at the chromosome end), subterminal (close to the chromosome end), intercalar (inside chromosome regardless of the centromere position), pericentric (close to the centromere), interstitial (central part of the chromosome arm), and distal (distal part of the chromosome arm).

Patterns of chiasma distribution during male meiosis were also analyzed. Mean chiasma frequency (f) was calculated during late prophase I (i.e., diplotene-diakinesis) as the total number of chiasmata divided by the total number of bivalents. Chiasma frequency was classified as low (1.0–1.19), moderate (1.2–1.59) or relatively high (1.6–2.49). Chiasma positions were classified as pericentric (the chiasma-centromere distance being less than 10% of the chromosome length), distal (the distance of the chiasma from the end of the arm being less than 10% of the chromosome length), or interstitial (positioned on the rest of arm). The term subdistal (distal 20% of the interstitial region) was used in some cases. If the length of the chromosome arm was equal to or shorter than 10% of the chromosome length, all its chiasmata were classified as pericentric. Centromere regions of most species were marked by a prominent knob or flexure during late prophase I, which enabled determination of the centromere position relative to that of the chiasmata. In the absence of these markers, data regarding the location of centromeric CH were obtained by C-banding or DAPI. However, it was seldom possible to determine centromere positions within all bivalents. Consequently, the proportions of particular types of chiasmata (e.g., pericentric, distal, interstitial) could only be approximated. In a few species, it was impossible to determine the position of the centromere on most bivalents. In such cases, the term intercalar is used for chiasmata whose distance from the end of the bivalent was more than 10% of the length of the chromosome.

### 2.7. Evolution of Chromosome Characters

The data were organized into several characters describing number and morphology of chromosomes, sex chromosomes, NORs, and constitutive heterochromatin for each order. The evolution of cytogenetic characters was reconstructed by character mapping on the latest molecular phylogeny of each order except for thelyphonids. Karyotype evolution of thelyphonids could not be analyzed by this method for the following reasons. First, some genera examined cytogenetically have not been included in any published molecular phylogeny. Second, there is no phylogeny of thelyphonids based on morphological characters. The analysis of thelyphonids was therefore based on hypotheses of their morphological evolution and karyotype evolution of related groups. The new data were augmented with data from published studies to reconstruct the karyotype evolution of analysed orders (Table 1). Questionable data [63,68] were excluded. Matrices and selected chromosome characters used in the reconstruction of karyotype evolution of each order are provided in Discussion.

## 3. Results

### 3.1. Amblypygi

#### 3.1.1. Charontidae

**Figure 1:**
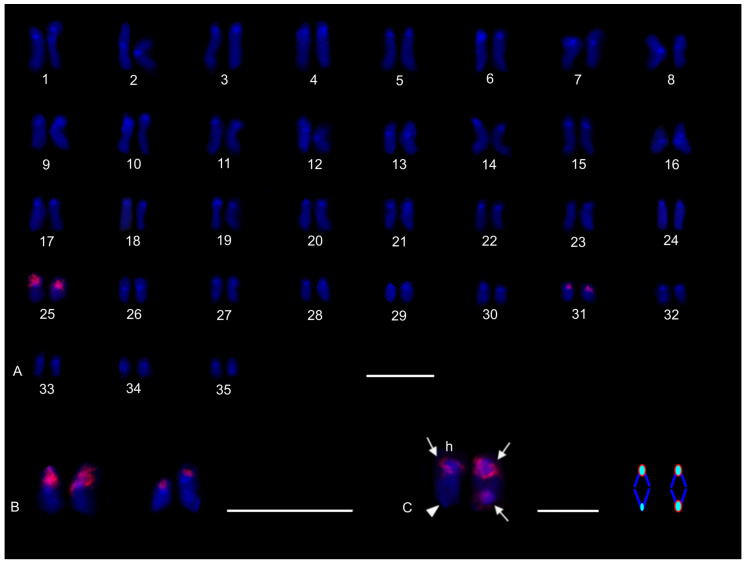
Amblypygi: Charontidae: *Charon* cf. *grayi*, female (**A**,**B**) and male (**C**); karyotype (**A**), chromosome pairs (**B**), and bivalents (**C**) indicating NORs (red) (FISH, DAPI staining). Centromeric blocks DAPI-positive. (**A**) Female karyotype (based on mitotic metaphase). Two acrocentric pairs (nos 25, 31) bear terminal NOR locus. (**B**) NOR-bearing chromosome pairs from another female mitotic metaphase, each with NOR locus at end of short arm. (**C**) Two NOR-bearing rod-like bivalents from male diakinesis (left) and their scheme (right). Left bivalent is heterozygous for presence of NOR; centromeric block of this bivalent associated with NOR (arrow) is larger than block without NOR (arrowhead). In the scheme, centromeric blocks with associated NOR are encircled by red. Abbreviation: h, bivalent heterozygous for presence of NOR. Arrow = centromeric block with associated NOR, arrowhead = centromeric block without associated NOR. Scale bars = 10 μm.

**Figure 2:**
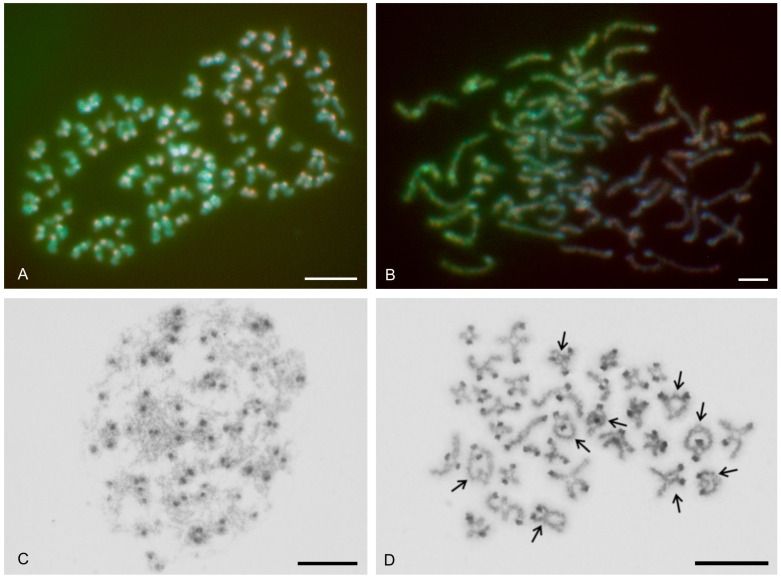
Amblypygi: Charontidae: *Charon* cf. *grayi*, CGH and meiotic division. (**A**,**B**) CGH with male and female genomic probes and DAPI counterstaining; chromosomes without male- (red) and female-specific (green) signals. Centromere regions exhibit bright fluorescence. (**A**) Two sister male metaphases II. (**B**) Female mitotic metaphase. (**C**,**D**) Male prophase I, Giemsa staining. (**C**) Diffuse stage. Bivalents despiralized except for centromeric regions. (**D**) Diplotene. Note considerable condensation of centromeres. Arrows = bivalents with two chiasmata. Scale bars = 10 μm.

The karyotype of the charontid examined, *Charon* cf. *grayi* (2n = 70), exhibited a predominance of monoarmed chromosomes. The karyotype included four metacentric (nos 2, 8, 16, 26) and three submetacentric (nos 7, 12, 13) chromosome pairs. Most biarmed chromosomes belonged to large and medium-sized pairs (Figure 1A). Centromeres were visualized as DAPI-positive regions (Figure 1). Two small acrocentric pairs bore an NOR locus at the end of the short arms (Figure 1A,B). One of these pairs was heterozygous for the presence of an NOR in males (i.e., one chromosome of the pair included NOR only). The NOR-bearing chromosome of the heterozygous pair had a larger DAPI-positive centromeric block than the chromosome without an NOR (see left bivalent in Figure 1C). Females were homozygous for both the presence of an NOR and the size of the centromeric block (Figure 1A,B). Comparative genomic hybridization did not detect sex chromosomes (Figure 2A,B). Male prophase I included a period of considerable despiralization of chromatin between the pachytene and diplotene stages (so-called diffuse stage; Figure 2C). Bivalents were decondensed except for the centromeric regions during this period. A male diffuse stage was also observed in other Pedipalpi and in Ricinulei. Following recondensation of chromatin, centromeres retained a high condensation (Figure 2D). Most bivalents had a single chiasma. Chiasma frequency was moderate (f = 1.24, *n* = 10). Most chiasmata were interstitial. Distal and pericentric chiasmata were also frequent, however. Pericentric chiasmata formed up to 32.4% of the chiasmata on a plate (*n* = 10). Acrocentric pairs often formed two chiasmata (up to nine bivalents per plate, *n* = 10), which were often located close to each other (Figure 2D).

#### 3.1.2. Phrynidae

**Figure 3:**
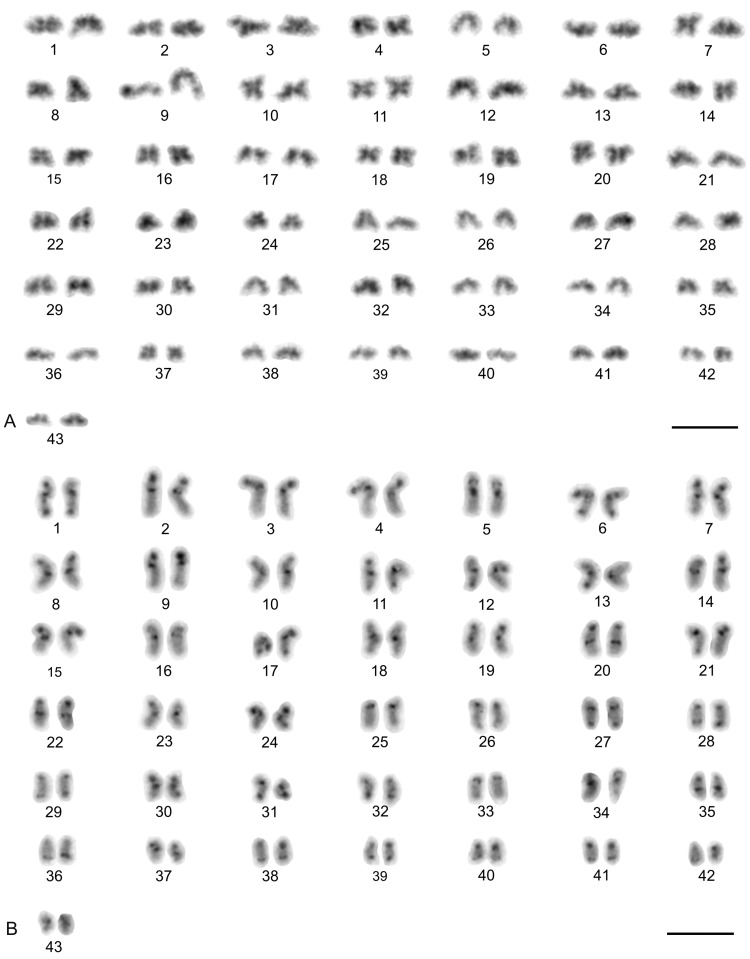
Amblypygi: Phrynidae: *Acanthophrynus coronatus*, male karyotypes, Giemsa staining. (**A**) Standard karyotype (based on two sister metaphases II). (**B**) C-banded karyotype (mitotic metaphase). Centromeric CH in all chromosome pairs; most non-acrocentric pairs terminated by CH at one or both ends. Scale bars = 10 μm.

**Figure 4:**
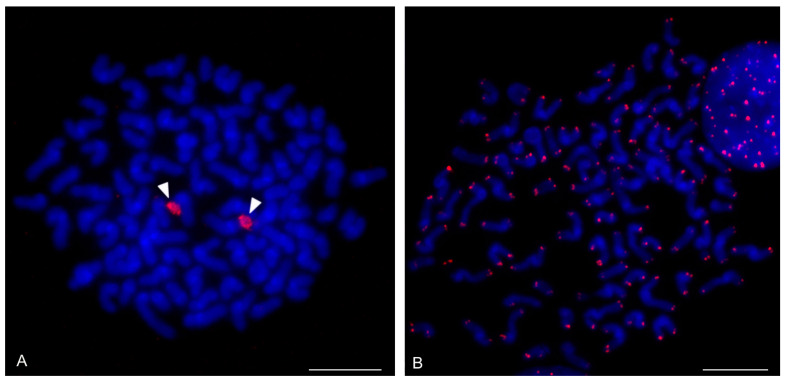
Amblypygi: Phrynidae: *Acanthophrynus coronatus*, male, pattern of NORs and telomeric repeats (red), mitotic metaphases (FISH, DAPI staining). (**A**) Visualization of NORs. Centromeres DAPI-positive. Chromosomes of subtelocentric pair each include NOR. (**B**) Visualization of telomeric repeats. Arrowhead = NOR. Scale bars = 10 μm.

The karyotypes of three phrynids (*Acanthophrynus coronatus*, *Heterophrynus* cf. *elaphus*, and *Paraphrynus mexicanus*) were examined. The karyotype of *A. coronatus* comprised a high number of chromosomes (2n = 86). The size of chromosome pairs decreased gradually (Figure 3A). Data from standard (Figure 3A) and C-banded preparations (Figure 3B) were compiled to determine the morphology of chromosome pairs. Chromosomes were remarkable for their relatively high CH content. All chromosome pairs contained a block of centromeric heterochromatin. In addition to centromeric CH, most non-acrocentric pairs bore a distal block, usually situated at the end of the short arm. Several non-acrocentric pairs bore a block at the ends of both arms (Figure 3B). The karyotype exhibited a predominance of biarmed chromosome pairs. Monoarmed chromosomes comprised six subtelocentric (nos 5, 9, 12, 21, 25, 34) and ten acrocentric (nos 26, 28, 29, 31, 33, 36, 38, 40–42) pairs. Most monoarmed chromosomes were small (Figure 3A,B). Most centromeres were visualized as tiny DAPI-positive regions; non-centromeric CH was not visualized by DAPI. One subtelocentric pair contained an NOR locus at the end of the short arm (Figure 4A). Chromosomes were terminated by (TTAGG)_n_ telomeric repeats (Figure 4B). Most pairs formed only one chiasma (f = 1.09, *n* = 10), usually situated in the distal or pericentric regions.

**Figure 5:**
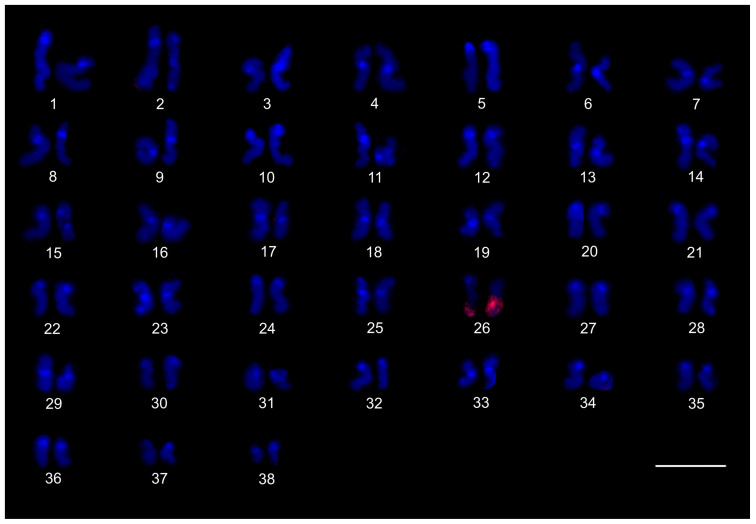
Amblypygi: Phrynidae: *Heterophrynus* cf. *elaphus*, male karyotype, illustrating pattern of NORs (red). Based on mitotic metaphase (FISH, DAPI staining). Centromeres DAPI-positive. Acrocentric pair no. 26 with NOR locus. Scale bar = 10 μm.

**Figure 6:**
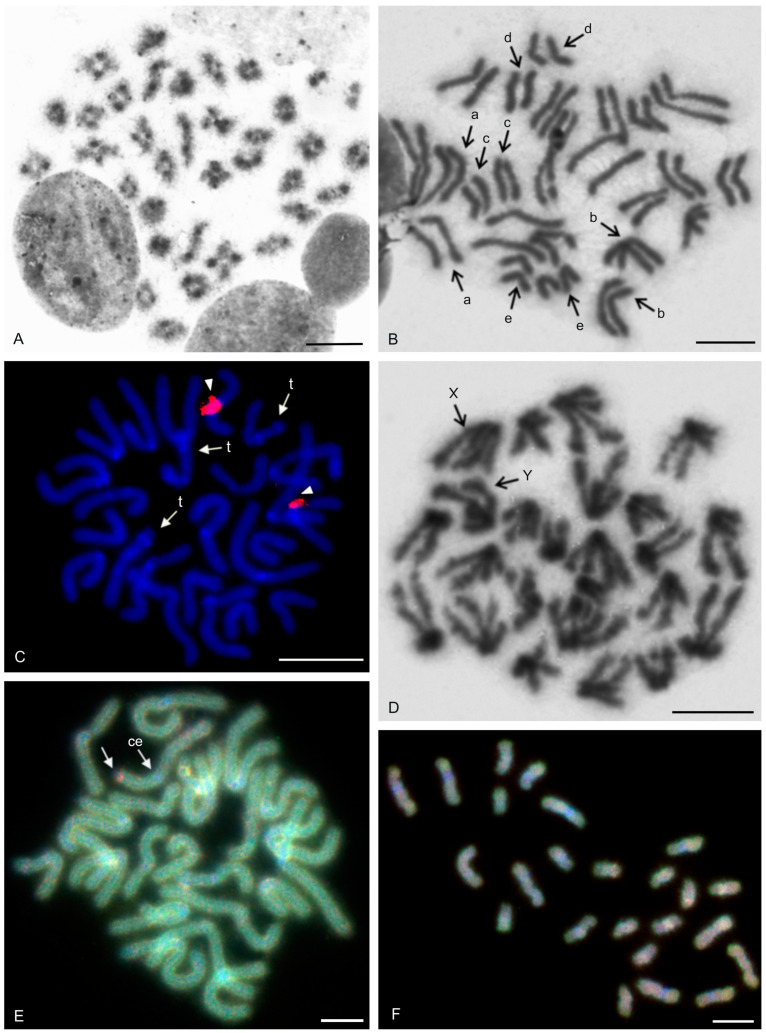
Amblypygi: Phrynidae: *Heterophrynus* cf. *elaphus* (**A**) and *Paraphrynus mexicanus* (**B**–**F**), chromosome plates. (**A**) Male diakinesis (Giemsa staining) indicating partial despiralization of bivalents. Centromeres form dark knobs. (**B**) Male, late spermatogonial metaphase (Giemsa staining), showing associations of homologs. Letter pairs indicate putative homologs. (**C**) Male mitotic metaphase, visualization of NORs (red) (FISH, DAPI staining). Centromeres DAPI-positive. Furthermore, chromosomes of two pairs include tiny subterminal DAPI-positive area. One metacentric pair with pericentric NOR locus. (**D**) Transition from metaphase to anaphase I (Giemsa staining), male. Note metacentric X and submetacentric Y chromosomes. (**E**,**F**) Sex chromosome detection by CGH with male (red) and female (green) genomic probes and DAPI counterstaining on male (**E**) and female (**F**) mitotic metaphase spreads. Y chromosome (**E**) with prominent centromere and male sex-specific signal. Female plate (**F**) without sex-specific signal. Abbreviations: ce, centromere; t, subterminal DAPI-positive area; X, X chromosome, Y, Y chromosome. Arrow = sex-specific signal, arrowhead = NOR. Scale bars = 10 μm.

The karyotype of *H.* cf. *elaphus* (2n = 76) is slightly predominated by monoarmed chromosome pairs. The karyotype contained 17 biarmed pairs (nos 3, 4, 6, 7, 9, 11, 13, 14, 16–19, 23, 25, 28, 29, 33) (Figure 5). Centromeres of mitotic chromosomes were visualized as DAPI-positive regions. One small acrocentric pair included an NOR locus at the end of the long arm (Figure 5). Male meiosis included an extremely long diffuse stage; chromosomes remained partially decondensed even at diakinesis (Figure 6A). Chromosomes also displayed a high despiralization at metaphase II.

The karyotype of *P. mexicanus* (2n = 24) consisted of biarmed chromosomes (Figure 6D). Homologous chromosomes of both sexes tended to be placed close to each other or were loosely associated during gonial mitosis (Figure 6B). The centromeres of approximately half the pairs were DAPI-positive. Moreover, two metacentric pairs contained a tiny DAPI-positive region at the subterminal part of one arm (Figure 6C). A pericentric NOR locus was present on chromosomes of a metacentric pair (seventh or eighth pair) (Figure 6C). The chromosomes of the fifth pair differed slightly in morphology, which was most visible during their separation at the transition from metaphase to anaphase I. One chromosome of the pair was metacentric, whereas the second was submetacentric (Figure 6D). The metacentric chromosome was 1.3× longer than the submetacentric one, which exhibited a male specific region at the terminal part of the short arm (Figure 6E). The female karyotype was without any sex specific signals (Figure 6F). Chiasma frequency during male meiosis was moderate (f = 1.3, *n* = 10). Almost all chiasmata were distal and subdistal.

#### 3.1.3. Phrynichidae

**Figure 7:**
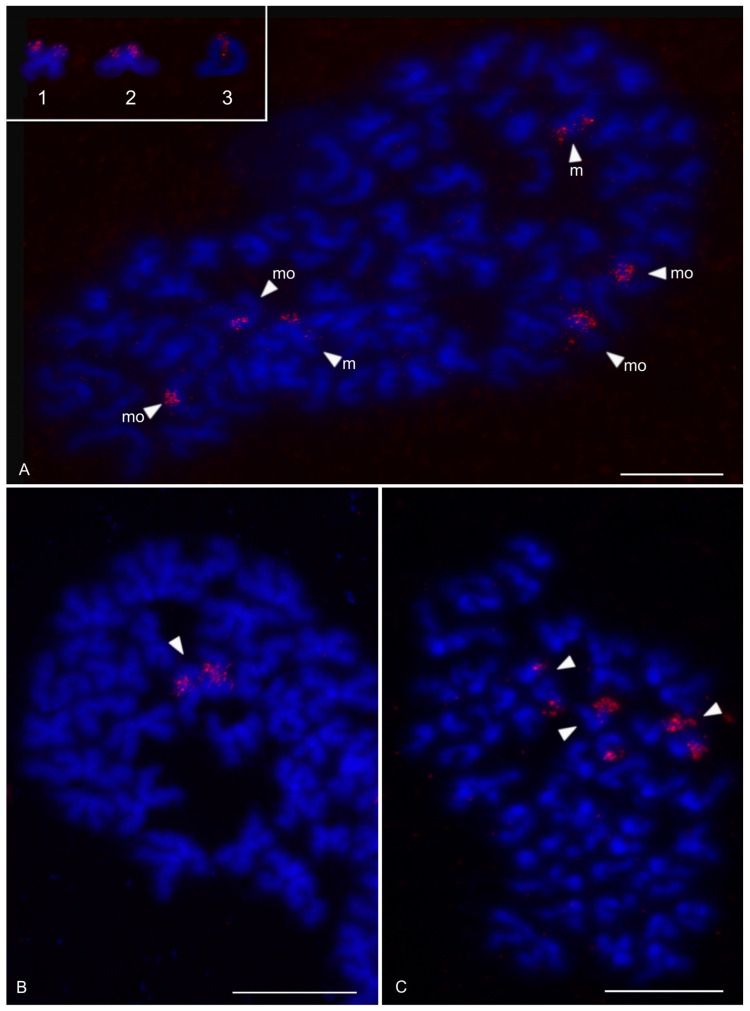
Amblypygi: Phrynichidae: *Euphrynichus amanica* (**A**), *E. bacillifer* (**B**), and *Phrynichus ceylonicus* (**C**), male plates illustrating patterns of NORs (red) (FISH, DAPI staining). (**A**) Two fused sister metaphases II. One metacentric pair with NOR locus and two monoarmed pairs each with NOR locus on short arm. All NOR loci are terminal. Inset: NOR-bearing chromosomes from another metaphase II. Each pair represented by one chromosome. Note metacentric (1), subtelocentric (2), and acrocentric morphology (3) of NOR-bearing chromosomes. (**B**) Metaphase II. Chromosome of metacentric pair with pericentric NOR. (**C**) Metaphase II. Three metacentric chromosomes (belonging to different chromosome pairs) each bear terminal NOR. Abbreviations: m, metacentric NOR-bearing chromosome; mo, monoarmed NOR-bearing chromosome. Arrowhead = NOR-bearing chromosome. Scale bars = 10 μm.

The karyotypes of four phrynichids (*Euphrynichus amanica*, *E. bacillifer*, *Phrynichus ceylonicus*, and *Damon medius*) were examined. The two *Euphrynichus* species differed greatly in 2n as well as in ratios of biarmed to monoarmed chromosome pairs. The karyotype of *E. amanica* (2n = 78) consisted of approximately the same portion of biarmed and monoarmed chromosome pairs. Biarmed chromosomes included fifteen metacentric (nos 5–7, 9, 12, 13, 20–23, 26–28, 32, 38) and four submetacentric (nos 11, 24, 29, 30) pairs (Figure S1). One acrocentric pair, one subtelocentric pair, and one metacentric pair each bore a terminal NOR locus. In the monoarmed pairs, NORs were situated on the short arms (Figure 7A). The karyotype of *E. bacillifer* (2n = 56) was formed by metacentric pairs, except for two submetacentric (nos 8, 10), one subtelocentric (no. 13), and three acrocentric (nos 12, 14, 18) pairs (Figure S2A). Some pairs had DAPI-positive centromeres. One medium-sized metacentric pair contained a pericentric NOR locus (Figure 7B). Chiasma frequency during male meiosis was low in *Euphrynichus* (f = 1.08, *n* = 10 in *E. amanica*; f = 1.16, *n* = 10 in *E. bacillifer*). Chiasmata were mostly distal and interstitial. Pericentric chiasmata were also common (up to thirteen per plate in *E. amanica*; up to seven in *E. bacillifer*) and formed by both biarmed and monoarmed pairs.

The karyotype of *P. ceylonicus* (2n = 52) was formed by metacentric chromosome pairs, except for three metacentric/submetacentric (nos 6, 9, 10), five submetacentric (nos 11, 16, 17, 18, 24), and one subtelocentric (no. 14) pairs (Figure S2B). Three small metacentric pairs each bore a terminal NOR locus (Figure 7C). Chiasma frequency during male meiosis was low (f = 1.07, *n* = 10); chiasmata were mostly distal and interstitial. Pericentric chiasmata were also frequent, sometimes forming up to one third of the chiasmata on a plate.

The metaphases II of *D. medius* contained both biarmed and monoarmed chromosomes. On these plates, two chromosomes (a large submetacentric and a medium-sized submetacentric element, each belonging to a different chromosome pair) bore a terminal NOR at the end of the short arm (Figure S3).

### 3.2. Thelyphonida

#### 3.2.1. Thelyphonidae (Hypoctoninae)

**Figure 8:**
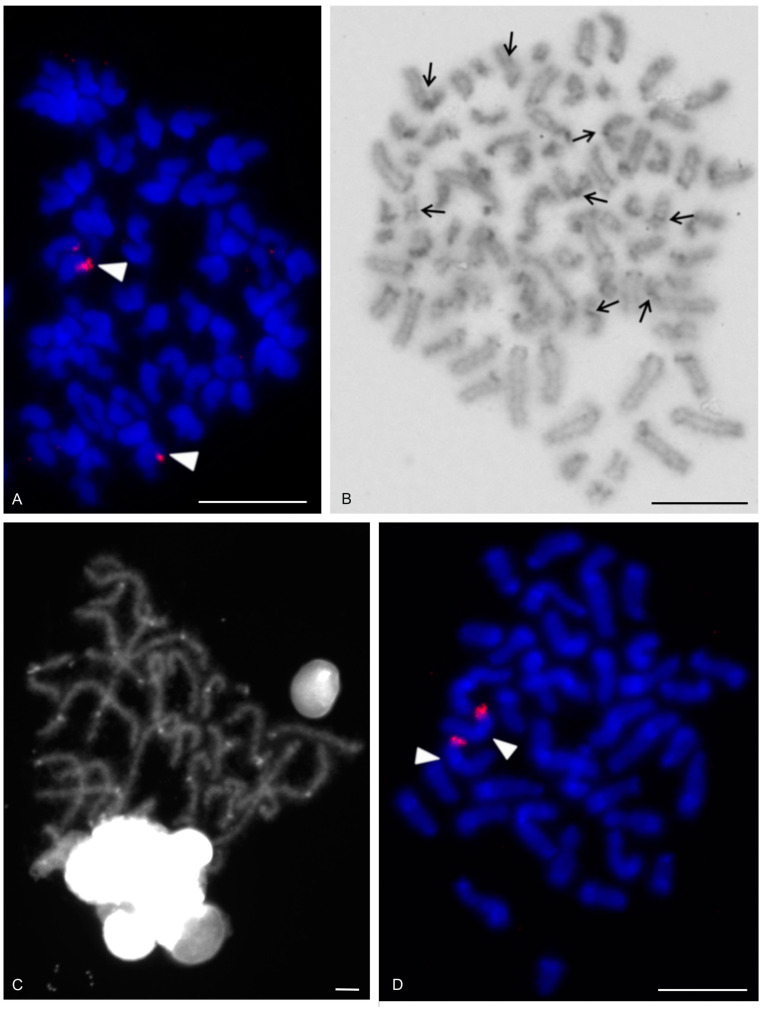
Thelyphonida: Thelyphonidae: Hypoctoninae: *Hypoctonus* cf. *gastrostictus* (**A**), *Labochirus proboscideus* (**B**,**C**), and *Yekuana venezolensis* (**D**), male, patterns of heterochromatin and NORs. (**A**) Metaphase II (FISH, DAPI staining). Two metacentric chromosomes (belonging to different chromosome pairs) each bear terminal NOR (red). (**B**) C-banded mitotic metaphase (Giemsa staining). Note tiny terminal (most chromosomes) or intercalary (arrows) blocks of CH. (**C**) Group of pachytene bivalents (DAPI staining). Note tiny DAPI-positive centromeric blocks. (**D**) Mitotic metaphase (FISH, DAPI staining). Centromeres DAPI-positive. Some chromosomes contain additional terminal DAPI-positive block. Short arms of submetacentric pair terminate by NOR locus (red). Arrowhead = NOR-bearing chromosome. Scale bars = 10 μm.

The karyotype of the three hypoctonines examined, *Hypoctonus* cf. *gastrostictus*, *Labochirus proboscideus*, and *Yekuana venezolensis*, differed considerably in diploid number and chromosome morphology. The karyotype of *H.* cf. *gastrostictus* (2n = 66) exhibited a predominance of biarmed chromosome pairs. Monoarmed pairs comprised two subtelocentric (nos 20, 26) and nine acrocentric (nos 7, 13, 16, 21–24, 29, 32) pairs (Figure S4A). Two metacentric pairs each bore a terminal NOR locus (Figure 8A). Chiasma frequency was moderate (f = 1.25, *n* = 10).

In contrast to the karyotype of *Hypoctonus*, the karyotype of *L. proboscideus* (2n = 78) exhibited a predominance of monoarmed chromosome pairs. Biarmed pairs comprised six metacentric (no. 1, 27, 29, 32, 34, 37), two metacentric/submetacentric (nos 18, 21), and three submetacentric (nos 4, 11, 26) pairs (Figure S4B). In some plates, two additional small pairs were also biarmed. Chromosomes had a low content of CH, which consisted mostly of small terminal and subterminal blocks. Intercalar CH was only present in four chromosome pairs (Figure 8B). This pattern of CH location suggests that most CH were centromeric. Fluorescent banding revealed that centromere regions were AT-rich (Figure 8C). Chiasma frequency during male meiosis was low (f = 1.16, *n* = 10). Some biarmed and monoarmed pairs formed two chiasmata (up to seven monoarmed pairs per plate, *n* = 6). Most chiasmata were distal and subdistal. A pericentric chiasma was often present in the monoarmed chromosome pairs (up to a quarter of the total number of chiasmata on a plate).

The karyotype of *Y. venezolensis* (2n = 38) consisted of approximately the same number of biarmed and monoarmed chromosome pairs. Monoarmed pairs comprised nine acrocentric (nos 7, 9, 11, 13, 14, 16–19) and one subtelocentric (no. 6) pairs. Biarmed pairs comprised four metacentric (nos 1, 2, 5, 15), one metacentric/submetacentric (no. 3), and four submetacentric (nos. 4, 8, 10, 12) pairs. Most biarmed chromosomes belonged to large and medium-sized pairs (Figure S5A). Centromeres were DAPI-positive. Approximately 30% of the chromosomes also bore additional terminal DAPI-positive region. The short arms of a middle-sized submetacentric pair (no. 4) were terminated by an NOR locus (Figure 8D). Chiasma frequency during male meiosis was moderate (f = 1.26, *n* = 10) and some bivalents had up to three chiasmata (one bivalent per ten plates). Chiasmata were mostly distal and pericentric.

#### 3.2.2. Thelyphonidae (Mastigoproctinae)

**Figure 9:**
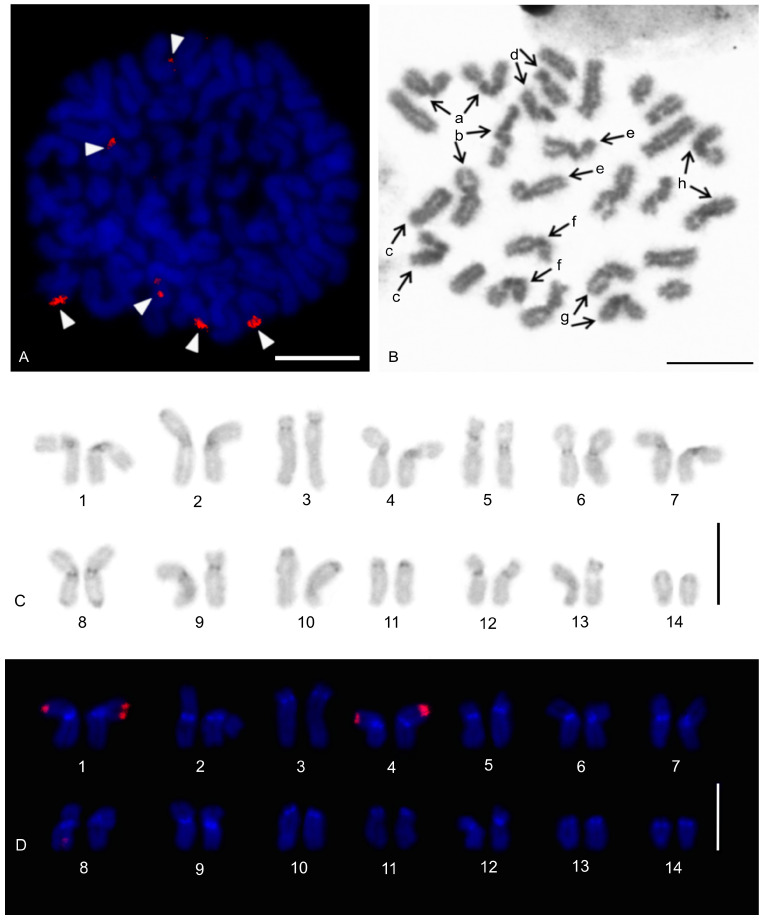
Thelyphonida: Thelyphonidae: Mastigoproctinae: *Uroproctus assamensis* (**A**) and *Mastigoproctus giganteus* (**B**–**D**), male mitotic metaphases. DAPI (**A**,**D**) and Giemsa (**B**,**C**) staining. (**A**) Visualization of NORs (red, FISH). Chromosomes of three pairs each with terminal NOR. (**B**) Associations of homologs. Letter pairs indicate putative homologs. (**C**) C-banded karyotype. Centromeric block of CH in all chromosome pairs; chromosomes of two pairs (second and eighth pair) with both centromeric and terminal CH. (**D**) Karyotype indicating pattern of NORs (red, FISH). Centromeres DAPI-positive. Two metacentric pairs each with terminal NOR locus. Arrowhead = NOR. Scale bar = 10 μm.

The karyotypes of the two mastigoproctines examined, *Uroproctus assamensis* and *Mastigoproctus giganteus*, differed considerably in diploid number and chromosome morphology. To determine chromosome morphology in *U. assamensis* (2n = 72), a haploid karyotype was used in which each chromosome pair was represented by a single chromosome (Figure S5B). The karyotype of this species exhibited a predominance of monoarmed chromosome pairs. Biarmed pairs comprised four metacentric (nos 1, 11, 17, 25), three submetacentric (nos 10, 14, 15), and one submetacentric/subtelocentric (no. 19) pairs. The morphology of the two smallest chromosome pairs was unresolved (Figure S5B). The first chromosome pair was markedly longer (6.4% of TCL) than the rest of the pairs in the karyotype, which decreased gradually in length from 4.6% of TCL (second longest pair) to 1% of TCL (shortest pair). The biarmed chromosomes belonged to large and medium-sized pairs (Figure S5B). Three pairs bore a terminal NOR locus (Figure 9A).

The karyotype of *M. giganteus* (2n = 28) is slightly predominated by biarmed chromosome pairs. Biarmed chromosomes comprised seven metacentric (nos 1, 2, 4, 6–9) and one submetacentric (no. 12) pairs. Monoarmed autosomes comprised one subtelocentric (no. 3) and four acrocentric (nos 10, 11, 13, 14) pairs. One pair (no. 5) exhibited submetacentric/subtelocentric morphology (Figure 9C,D). Chromosome pairs decreased gradually in size. Most monoarmed pairs were small (Figure 9C,D). Homologous chromosomes were often situated close to each other during spermatogonial mitosis (Figure 9B). Constitutive heterochromatin was mostly centromeric (Figure 9C). Centromeric regions of *M. giganteus* differed from those found in *U. assamensis* in two respects. Primary constrictions of chromosomes were clearly distinguishable during the mitotic metaphase (Figure 9B) and the centromere regions were DAPI positive (Figure 9D). Two large metacentric pairs (first and fourth chromosome pairs) of *M. giganteus* each contained a terminal NOR locus (Figure 9D). Chiasma frequency during male meiosis was low in the mastigoproctines (f = 1.17, *n* = 5 in *U. assamensis*; f = 1.05, *n* = 20 in *M. giganteus*). The chiasmata of *M. giganteus* were mostly distal, subdistal, and pericentric. Pericentric chiasmata were formed by both monoarmed and biarmed pairs.

#### 3.2.3. Thelyphonidae (Thelyphoninae)

**Figure 10:**
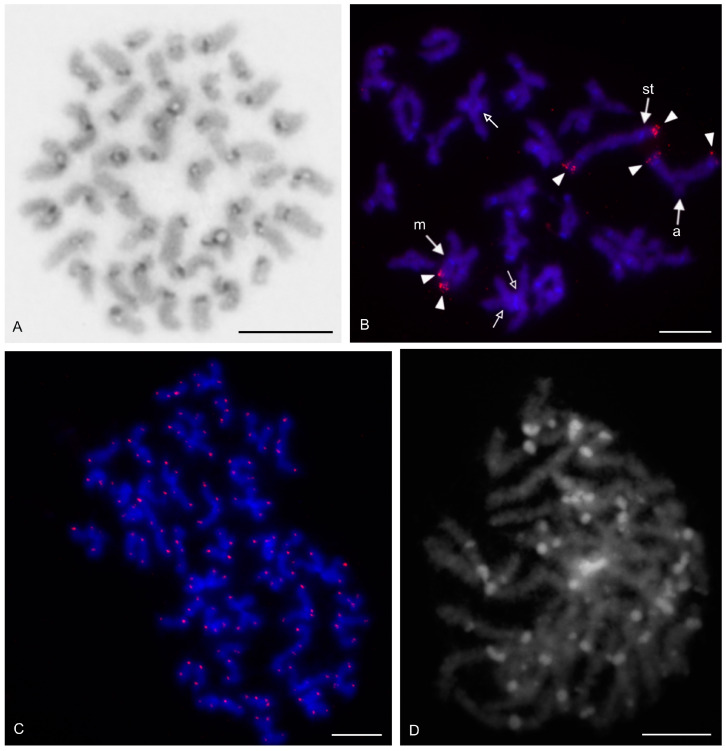
Thelyphonida: Thelyphonidae: Thelyphoninae: *Ginosigma* sp. (**A**–**C**) and *Thelyphonus* cf. *linganus* (**D**), male plates, patterns of heterochromatin, NORs, and telomeric repeats. Giemsa (**A**) and DAPI (**B**–**D**) staining. (**A**) C-banded mitotic metaphase. Centromeric CH in all chromosomes. (**B**) Diplotene, visualization of NORs (red, FISH). Centromeric regions are DAPI positive. Terminal NOR locus in metacentric, subtelocentric, and acrocentric bivalent. (**C**) Two sister metaphases II, visualization of telomeric repeats (red, FISH). (**D**) Pachytene (DAPI staining), bivalents exhibit prominent AT-rich knobs. Abbreviations: a, acrocentric bivalent; m, metacentric bivalent; st, subtelocentric bivalent. Arrowhead = NOR; open arrow = pericentric chiasma. Scale bars = 10 μm.

The karyotypes of the two thelyphonines examined, *Ginosigma* sp. and *Thelyphonus* cf. *linganus* differed considerably in diploid number and chromosome morphology. The karyotype of *Ginosigma* sp. (2n = 40) was formed by four metacentric (nos 1, 2, 4, 15), four submetacentric (nos 11, 14, 16, 17), one submetacentric/subtelocentric (no. 6), four subtelocentric (nos 3, 5, 7, 8), and seven acrocentric (nos. 9, 10, 12, 13, 18–20) chromosome pairs (Figure S6A). Heterochromatin was restricted to the centromere areas; all chromosomes included a centromeric block of CH (Figure 10A). These blocks were DAPI-positive (Figure 10B). Three chromosome pairs had an NOR locus at the end of the short arms. One NOR-bearing pair was metacentric (probably the second or fourth chromosome pair), one submetacentric/subtelocentric (probably the sixth chromosome pair), and one subtelocentric (most probably the eighth chromosome pair) (Figure 10B). Chromosomes were terminated by (TTAGG)_n_ telomeric repeats (Figure 10C). Chiasma frequency during male meiosis was moderate (f = 1.26, *n* = 10). All positions of chiasmata (pericentric, distal, and interstitial) were frequent. Pericentric chiasmata were formed by both biarmed and monoarmed chromosomes (Figure 10B).

The karyotype of *T.* cf. *linganus* (2n = 66) exhibited a predominance of biarmed chromosome pairs. Monoarmed autosomes comprised three subtelocentric (nos 1, 10, 29) and ten acrocentric (nos 4, 12, 14, 16, 18, 21, 22, 30–32) pairs (Figure S6B). Prominent AT-rich knobs were present in pachytene bivalents after fluorescent banding (Figure 10D). The distribution of these knobs suggests that most correspond to centromeric CH.

**Figure 11:**
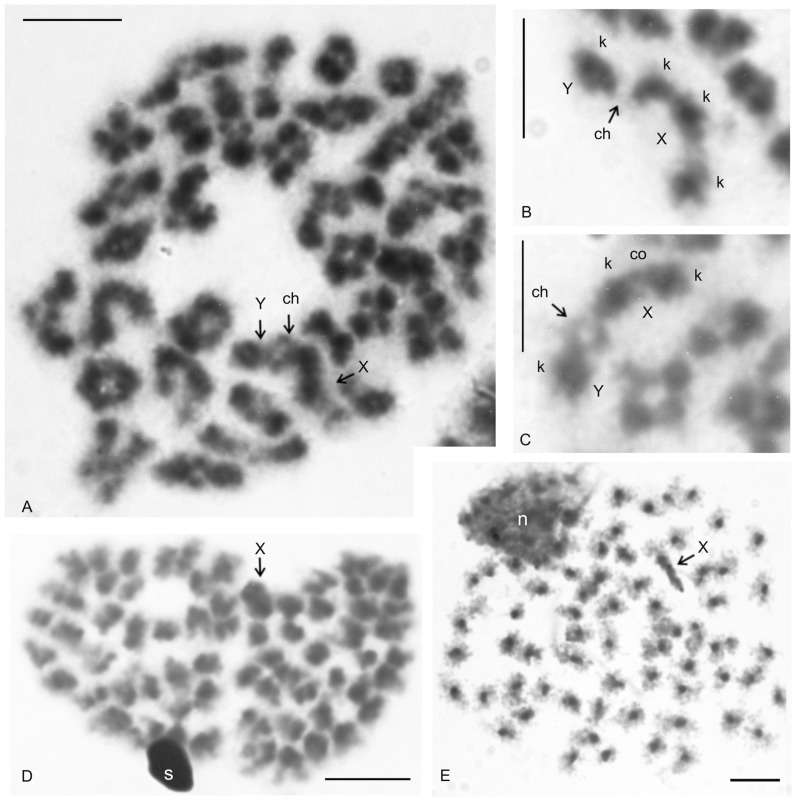
Thelyphonida: Thelyphonidae: Thelyphoninae: *Thelyphonus* cf. *linganus*, behaviour of male sex chromosomes. (**A**) Diakinesis, note heterobivalent comprising larger (putative X) and smaller (putative Y) chromosomes. (**B**,**C**) Sex chromosome bivalent during early (**B**) and late (**C**) diplotene. X chromosome includes three knobs at early diplotene (**B**), two of which fuse during late diplotene; fusion of knobs accompanied by formation of constriction between remaining two knobs (**C**). Y chromosome contains one knob throughout diplotene and diakinesis (**A**–**C**). (**D**) Anaphase I, note heterochromatinization of putative X chromosome. (**E**) Unusual interphase nucleus containing fuzzy chromosomes. Putative X chromosome forms heterochromatic body. Abbreviations: ch, chiasma; co, constriction; k, knob; n, deformed interphase nucleus; s, sperm nucleus; X, putative X chromosome; Y, putative Y chromosome. Scale bars = 10 μm.

A putative heteromorphic sex chromosome pair was present in the karyotype of *T.* cf. *linganus*. The chromosomes of the pair differed considerably in size (Figure 11A) and morphology. The large chromosome was 1.9× longer than the shorter one at diakinesis. One sex chromosome, the putative X chromosome, had a submetacentric morphology, and the second sex chromosome, the putative Y chromosome, had a metacentric morphology (Figure S6B). The putative X chromosome contained three knobs during early diplotene (Figure 11B). Two of these knobs were close to each other and fused during late diplotene, which was followed by the formation of a prominent constriction between the remaining two knobs (Figure 11C). The putative Y chromosome had only a single knob (Figure 11A–C). The putative X chromosome was heterochromatic in some anaphase I and II plates (Figure 11D) and in specific interphase nuclei, which were common in testes tissue (Figure 11E). These interphase nuclei contained a diploid number of fuzzy, dot-like or rod-like chromosomes. Nuclei of similar appearance were also observed in the testes of other Pedipalpi. During diplotene, a single distal chiasma was formed between the sex chromosomes (Figure 11A,C). Concerning the putative X chromosome, the only region containing two adjacent knobs partook in formation of the chiasma (Figure 11B). Chiasma frequency of autosomes during male meiosis was low (f = 1.09, *n* = 5).

#### 3.2.4. Thelyphonidae (Typopeltinae)

**Figure 12:**
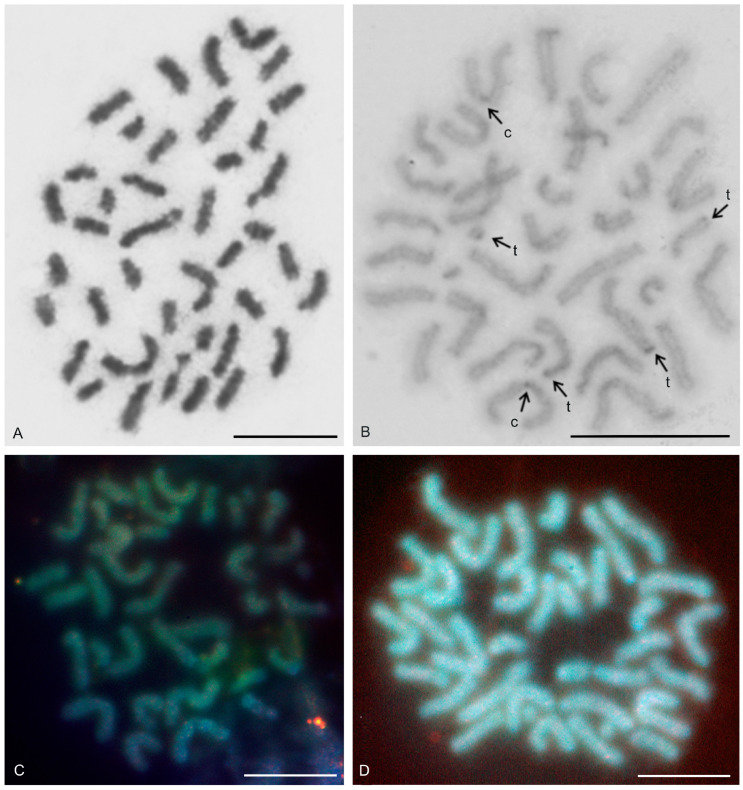
Thelyphonida: Thelyphonidae: Typopeltinae: *Typopeltis crucifer* (**A**) and *T. guangxiensis* (**B**–**D**), male (**B**,**C**) and female (**A**,**D**) plates. (**A**) Mitotic metaphase. (**B**) C-banded mitotic metaphase (Giemsa staining). Small intercalary or terminal block of CH present in several chromosome pairs. (**C**,**D**) Mitotic metaphase of male (**C**) and female (**D**), sex chromosome detection by CGH with male and female genomic probes and DAPI counterstaining. Chromosomes do not contain any male- (red) and female-specific (green) signal. Abbreviations: c, centromeric block of CH; t, terminal block of CH. Scale bars = 10 μm.

**Figure 13:**
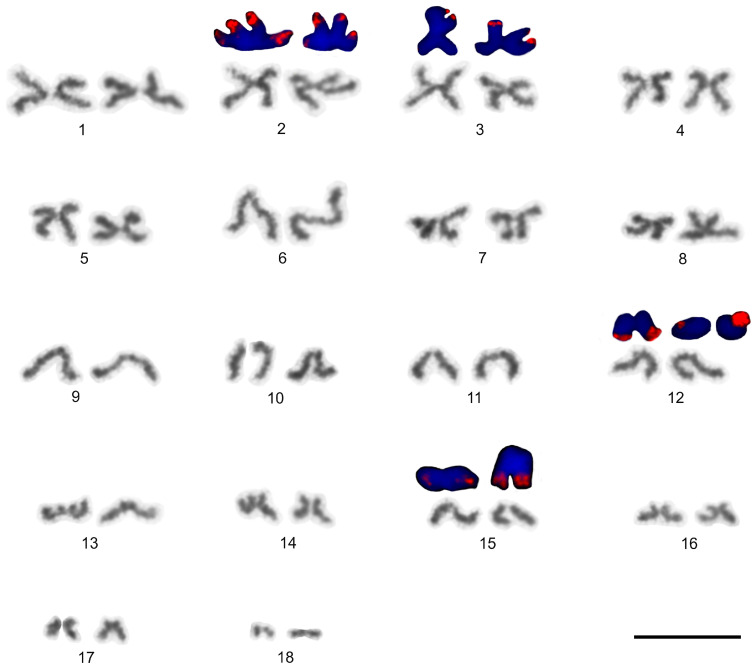
Thelyphonida: Thelyphonidae: Typopeltinae: *Typopeltis guangxiensis*, male karyotype, based on two sister metaphases II (Giemsa staining). Above standard karyotype shown NOR-bearing chromosomes (blue) from another two sister metaphases II (FISH, DAPI staining). One metacentric (no. 3) and two acrocentric pairs (nos 12, 15) each bear single terminal NOR locus (red). Another metacentric pair (no. 2) contains two terminal NOR loci (red). Scale bar = 10 μm.

The karyotypes of the two typopeltines examined, *Typopeltis crucifer* and *T. guangxiensis*, differed in diploid number. The mitotic chromosomes of the *T. crucifer* female (2n = 40) had an indistinct primary constriction, which prevented determination of the chromosome morphology (Figure 12A). The karyotype of *T. guangxiensis* (2n = 36) consisted of approximately the same portion of biarmed and monoarmed chromosome pairs, namely six metacentric (nos 1–3, 5, 7, 8), four submetacentric (nos 4, 14, 16, 17), one subtelocentric (no. 13), and seven acrocentric (nos 6, 9–12, 15, 18) pairs. Most acrocentrics belonged to medium-sized pairs (Figure 13). Chromosome pairs decreased gradually in size (8.9% to 3.3% of TCL), except for the smallest pair which was tiny (2% of TCL). Chromosomes of the smallest pair were often situated close to each other during gonial mitosis in both sexes (Figure 12B). Mitotic chromosomes had a very low CH content. A small block of CH was present in the centromeric area of a large metacentric pair. A small block of CH was also present at one end of several chromosome pairs, including the smallest pair (Figure 12B). Comparative genomic hybridization did not reveal sex chromosomes in the karyotype (Figure 12C,D). Chiasma frequency during male meiosis was relatively high (f = 1.72, *n* = 20); acrocentric pairs often had two chiasmata and, on four plates, three chiasmata were present in at least one, and sometimes two, of the bivalents. Chiasmata were mostly distal and interstitial.

Two large metacentrics (second and third chromosome pairs) and two small acrocentrics (twelfth and fifteenth chromosome pairs) of *T. guangxiensis* had an NOR locus. Both acrocentric pairs and one metacentric pair had a single terminal NOR locus, situated at the end of the long arm in acrocentrics (Figure 13). The second metacentric pair had an NOR locus at each end (Figure 13) whereas one NOR locus was polymorphic in NOR number. One male and one female were homozygous for the NOR (Figure 13), whereas another male was heterozygous for the presence of an NOR.

### 3.3. Schizomida

**Figure 14:**
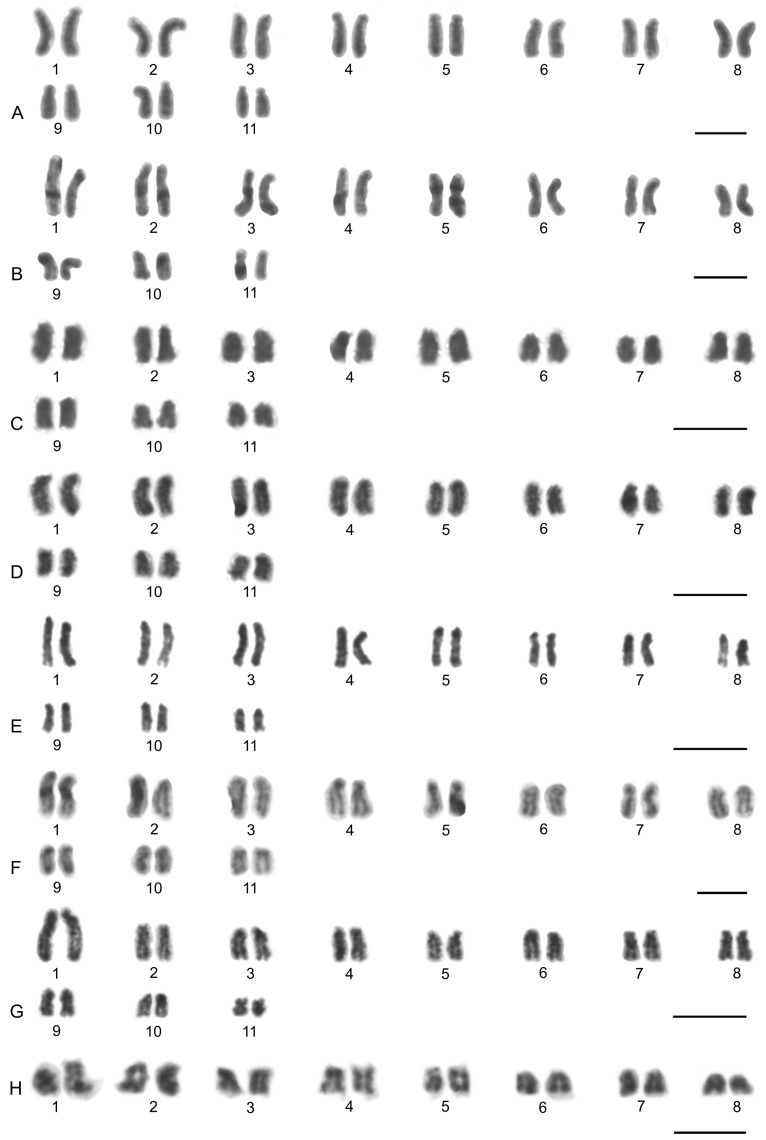
Schizomida: *Stenochrus* sp. (**A**), *Olmecazomus brujo* (**B**), Hubbardiidae (Cameroon) (**C**), Hubbardiidae (Seychelles) (**D**), *Agastoschizomus lucifer* (**E**), *Notozomus* sp. (**F**), *Clavizomus* sp. (**G**), *Orientzomus* sp. (Luzon) (**H**), male (**B**,**F**,**G**) and female (**A**,**C**–**E**,**H**) karyotypes. Based on mitotic metaphase (Giemsa staining). Scale bars = 10 μm.

**Figure 15:**
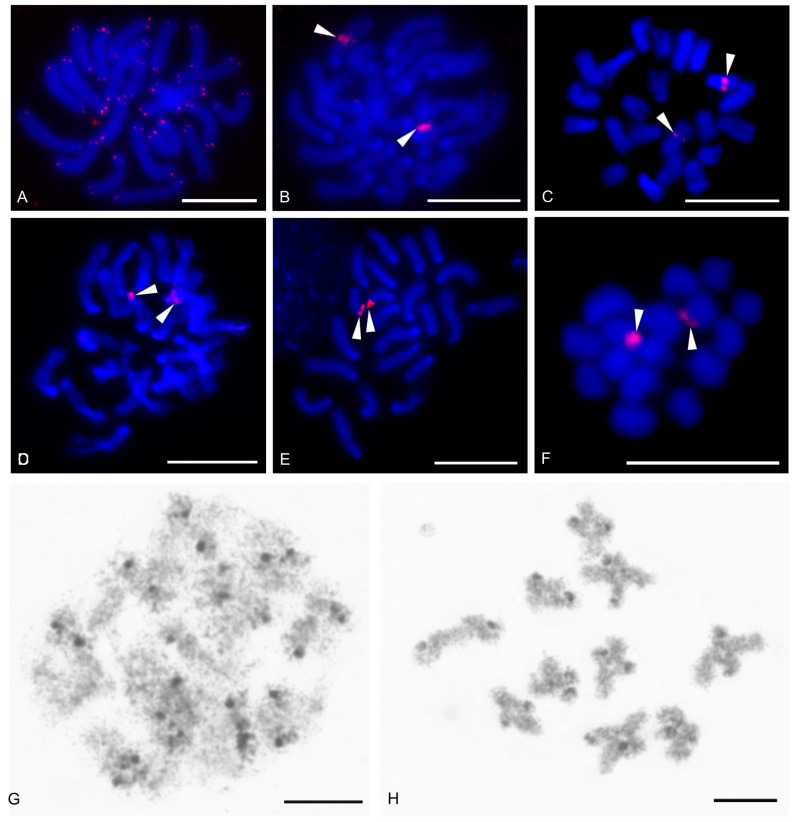
Schizomida: *Stenochrus* sp. (**A**), *Olmecazomus brujo* (**B**,**G**,**H**), Hubbardiidae (Cameroon) (**C**), Hubbardiidae (Seychelles) (**D**), *Agastoschizomus lucifer* (**E**), and *Orientzomus* sp. (Mindanao) (**F**), pattern of NORs, telomeric repeats (DAPI staining), and meiosis (Giemsa staining). Male (**B**,**G**,**H**) and female (**A**,**C**–**F**) plates. (**A**) Mitotic metaphase, visualization of telomeric repeats (red, FISH). (**B**–**F**) Mitotic metaphases, visualization of NORs (red, FISH). (**B**) One chromosome pair with interstitial NOR locus. All chromosomes exhibit DAPI-positive centromere region at one end. (**C**) One chromosome pair with interstitial NOR locus. (**D**) One chromosome pair with terminal NOR locus. (**E**) One chromosome pair with terminal NOR locus. Most chromosomes exhibit DAPI-positive centromere region at one end. (**F**) One chromosome pair with terminal NOR locus. (**G**) Diffuse stage, note considerable decondensation of bivalents except for centromeric regions (dark spots). (**H**) Incomplete diplotene showing ten bivalents. Each bivalent contains one chiasma. Note dark knobs formed by centromere regions. Arrowhead = NOR. Scale bars = 10 μm.

Examined schizomids belonged to *Clavizomus*, *Notozomus*, *Olmecazomus*, *Orientzomus*, *Stenochrus* (Hubbardiidae), *Agastoschizomus* (Protoschizomidae), and two hubbardiids not determined to genus. Their karyotypes had low diploid numbers. The karyotypes of *A. lucifer*, *Notozomus* sp., *O. brujo*, *Stenochrus* sp., and the undetermined hubbardiids had the same diploid number (2n = 22) and were formed by monoarmed chromosome pairs. The chromosome pairs of these species decreased gradually in size (Figure 14A–F). Most chromosome pairs of *Stenochrus* sp. were subtelocentric (nos 1, 2, 4–7, 10, 11) (Figure 14A) and terminated by (TTAGG)_n_ telomeric repeats (Figure 15A). The chromosomes of *Notozomus* were acrocentric except for two subtelocentric pairs (nos 4, 5) (Figure 14F). The chromosomes of *Agastoschizomus*, *Olmecazomus*, and the undetermined hubbardiids were exclusively acrocentric (Figure 14B–E). The chromosomes of *Olmecazomus* contained large DAPI-positive centromeric regions (Figure 15B). Most chromosomes of *Agastoschizomus* also contained DAPI-positive centromeres (Figure 15E). The karyotype of *Clavizomus* also comprised eleven chromosome pairs which were acrocentric except for one metacentric (no. 11) and three subtelocentric (nos 1, 5, 8) pairs (Figure 14G). Chromosomes of this schizomid could be divided into three size groups: one long (16.7% of TCL), nine medium-sized (from 9.7% to 6.7% of TCL), and one small (5.4% of TCL) pair. The karyotype of *Orientzomus* differed from the other schizomids in its lower diploid number (2n = 16). The chromosome pairs of *Orientzomus* sp. from Luzon Island decreased gradually in size. Five pairs (nos 1–5) were biarmed. The other pairs appeared to exhibit acrocentric morphology (Figure 14H). The karyotypes of schizomid males and females did not include a heteromorphic sex chromosome pair (Figure 14).

One chromosome pair of schizomids included an NOR locus (Figure 15B–F), which was interstitial in *Olmecazomus* and the hubbardiid from Cameroon (Figure 15B,C), and terminal in the hubbardiid from the Seychelles, *Agastoschizomus* and *Orientzomus* from Mindanao (Figure 15D–F). The morphology of the NOR-bearing pair is unknown in *Orientzomus*. In the other schizomids used for NOR detection, all chromosome pairs were acrocentric, including the NOR-bearing pair. 

The present study also included an analysis of meiotic division in schizomids. In *Olmecazomus*, a complete meiotic sequence was observed in adult males, and plates of prophase I observed in adult females and juveniles of both sexes. Centromere regions formed a prominent knob during prophase I (Figure 15G,H). Male prophase I included a diffuse stage between pachytene and diplotene (Figure 15G). Bivalents included chiasmata. Chiasma frequency during male meiosis was low (f = 1.05, *n* = 5); most chiasmata were interstitial and pericentric (Figure 15H).

### 3.4. Ricinulei

**Figure 16:**
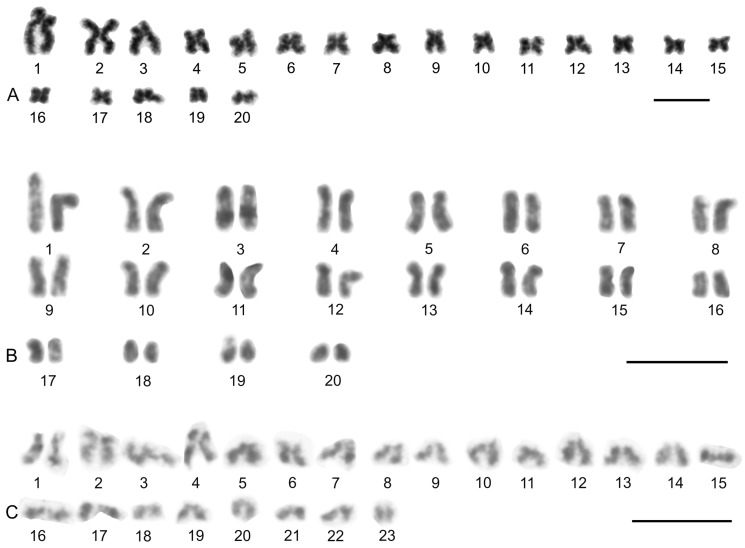
Ricinulei: male (**A**,**C**) and female (**B**) karyotypes (Giemsa staining). Two karyotypes (**A**,**C**) are haploid (each chromosome pair is represented by one chromosome). (**A**) *Ricinoides olounoua* (metaphase II). (**B**) *Pseudocellus gertschi* (mitotic metaphase). (**C**) *Cryptocellus narino* (metaphase II). Scale bars = 10 μm.

The karyotypes of the ricinuleids examined, *Cryptocellus narino*, *Pseudocellus gertschi*, and *Ricinoides olounoua*, had similar chromosome numbers (*C. narino* 2n = 46; *P. gertschi* and *R. olounoua* 2n = 40) (Figure 16A–C). The male and female karyotypes of ricinuleids did not include any heteromorphic sex chromosome pair (Figure 16B and Figure S7D). The karyotypes of particular ricinuleids differed in the proportion of biarmed and monoarmed chromosome pairs. The chromosomes of *R. olounoua* were mostly biarmed except for four subtelocentric pairs (nos 1, 3, 9, 19) and formed two size groups: first to third chromosome pair (from 10.99 to 7.69% of TCL) and fourth to twentieth chromosome pair (from 5.46 to 2.52% of TCL) (Figure 16A). The karyotype of *P. gertschi* had a predominance of monoarmed chromosome pairs. Biarmed chromosomes comprised two metacentric (nos 13, 17) and four submetacentric pairs (nos 1, 7, 10, 12). One pair (no. 2) was submetacentric/subtelocentric (Figure 16B). Chromosome pairs formed three size groups: first chromosome pair (8.8% of TCL), second to fifteenth chromosome pair (from 7.1% to 4.5% of TCL), and sixteenth to twentieth chromosome pair (from 3.5% to 2.1% of TCL). The karyotype of *C. narino* also had a predominance of monoarmed chromosome pairs. All monoarmed chromosomes were acrocentric except for one subtelocentric pair (no. 5). Biarmed chromosome pairs comprised one metacentric (no. 6), one metacentric/submetacentric (no. 4), and two submetacentric (nos 2, 11) pairs (Figure 16C).

**Figure 17:**
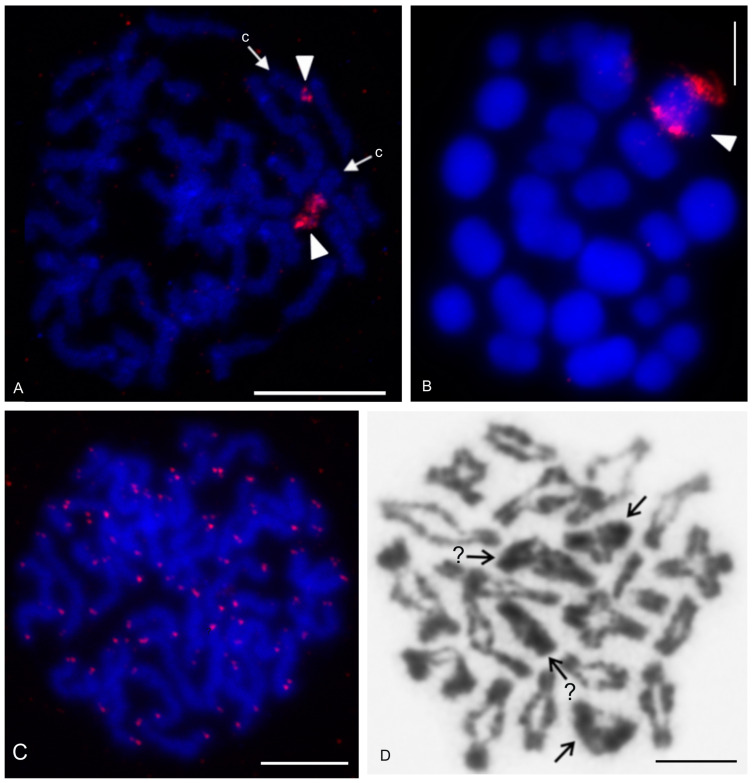
Ricinulei: *Pseudocellus gertschi* (**A**,**C**) and *Cryptocellus narino* (**B**,**D**), males, patterns of NORs, telomeric repeats (DAPI staining), and meiosis (Giemsa staining). (**A**) Mitotic metaphase, visualization of NORs (red, FISH). Centromere regions are tiny, DAPI-positive. Long arm of chromosomes of acrocentric pair each terminating by NOR. Note considerable difference in size of NORs on two chromosomes (c–centromere of NOR-bearing chromosome). (**B**) Metaphase I, visualization of NORs (red, FISH), indicating NOR-bearing bivalent. (**C**) Mitotic metaphase, visualization of telomeric repeats (red, FISH). (**D**) Diplotene. Most bivalents contain one chiasma; two chiasmata unambiguous in two bivalents (arrows), and ambiguous in another two bivalents (marked by ?). Arrowhead = NOR-bearing chromosome (**A**) or bivalent (**B**). Scale bars = 5 μm (**B**,**D**), 10 μm (**A**,**C**).

The present study detected NORs, telomeric repeats and analysed meiotic division in ricinuleids. One chromosome pair of ricinuleids bore an NOR locus (Figure 17A,B and Figure S7A,B) which was subterminal in *Cryptocellus* (Figure S7A) and *Ricinoides* (Figure S7B), and terminal in *Pseudocellus* (Figure 17A). In the latter two ricinuleids, it was a small pair. The chromosomes of the NOR-bearing pair displayed considerable NOR size heteromorphism in the specimen of *Pseudocellus* examined (Figure 17A). The chromosomes of this ricinuleid were terminated by (TTAGG)_n_ telomeric repeats (Figure 17C). A complete sequence of male meiotic division was observed in all examined ricinuleids. Chromatin underwent a considerable decondensation between pachytene and diplotene (Figure S7C). Following recondensation, bivalents showed chiasmata. Chiasma frequency during male meiosis was low (f = 1.15, *n* = 10 in *Ricinoides*; f = 1.10, *n* = 5 in *Cryptocellus*) (Figure 17D and Figure S7D) to moderate (f = 1.2, *n* = 4 in *Pseudocellus*). Most chiasmata were distal and intercalar. Bivalents with two chiasmata were formed by the four largest chromosome pairs only, in *Ricinoides*.

### 3.5. Solifugae

**Figure 18:**
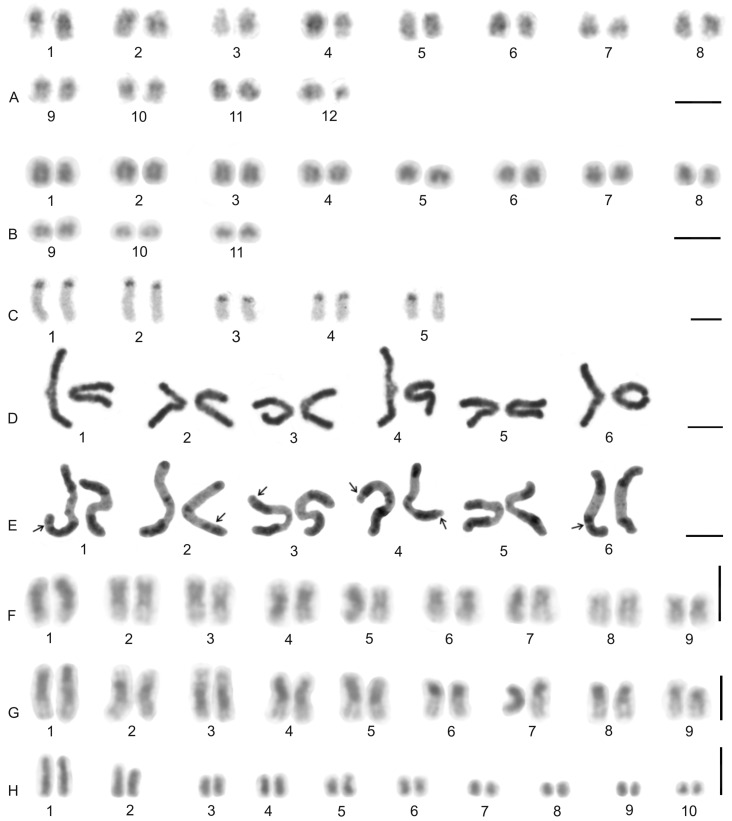
Solifugae: Ammotrechidae (**A**), Eremobatidae (**B**), Daesiidae (**C**), Galeodidae (**D**,**E**), Rhagodidae (**F**,**G**), and Solpugidae (**H**), karyotypes. Based on mitotic metaphase (Giemsa staining) of female (**C**,**D**,**H**), male (**A**,**B**,**E**) or postembryo (**F**,**G**). (**A**) *Ammotrechula mulaiki*. (**B**) *Eremobates similis*. (**C**) *Gluvia dorsalis*, C-banding. Acrocentric karyotype; centromere regions formed by prominent block of CH. (**D**) *Paragaleodes pallidus*, standard karyotype. Karyotype is metacentric. (**E**) *P. pallidus*, C-banded karyotype. Both arms of each pair terminating by large block of CH. Note narrow euchromatic regions which intercalate blocks of CH (arrows). Centromere regions contain small single or double block of CH. (**F**,**G**) *Rhagodes* sp., standard karyotype (**F**) and C-banded karyotype (**G**). Most pairs include large block of CH which forms centromere and surrounding regions (metacentric pairs nos 2, 3) or centromere and arm (metacentric pairs nos 1, 4, 5, 7; subtelocentric pairs 6, 8). (**H**) *Solpugista* sp., acrocentric karyotype. Scale bars = 10 μm.

**Figure 19:**
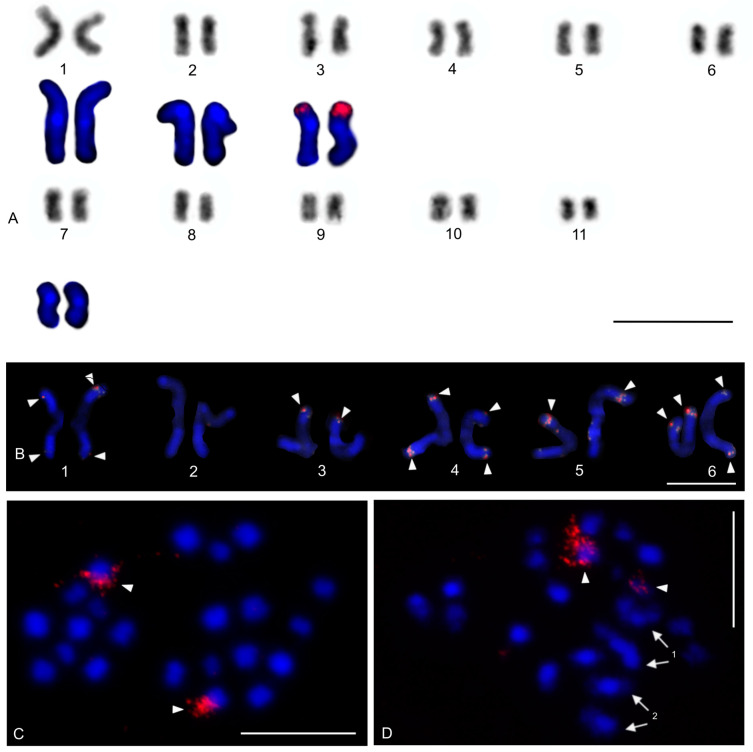
Solifugae: Daesiidae (**A**), Galeodidae (**B**), Eremobatidae (**C**), Solpugidae (**D**), male (**A**,**C**) and female (**B**,**D**) pattern of NORs (red, FISH). Based on mitotic metaphase (DAPI stained, unless otherwise stated). (**A**) *Eberlanzia flava* karyotype (Giemsa staining), mostly acrocentric. Morphology of non-acrocentric pairs shown below standard karyotype using DAPI-stained chromosomes from another plate, centromeres DAPI-positive. Third pair terminating by NOR locus. (**B**) *Paragaleodes pallidus* karyotype. Two pairs (nos 3, 5) bear NOR locus. Three pairs (nos 1, 4, 6) contain two NOR loci, whereas each of two chromosome arms includes one locus. Each NOR locus embedded into large distal block of DAPI-positive CH. (**C**) *Eremobates similis*. One pair bears large terminal NOR locus. (**D**) *Solpugista* sp. One small pair bears NOR locus. Note considerable difference in size of NOR on chromosomes. Homologous chromosomes of two long pairs associated (nos 1 and 2 indicated on image). Arrowheads = NORs. Scale bar = 10 μm.

Representatives of six solifuge families were examined; their karyotypes had low diploid numbers. The male and female karyotypes of solifuges did not include any heteromorphic sex chromosome pair (Figure 18 and Figure 19A). Homologous chromosomes of solifuges often associated during mitosis, sometimes arranged in parallel (Figure S8A). Interphase nuclei often contained a haploid number of heterochromatic blocks, suggesting an association of homologous chromosomes also during interphase (Figure S8B).

#### 3.5.1. Ammotrechidae and Eremobatidae

The karyotypes of the New World solifuges examined, an ammotrechid (*Ammotrechula mulaiki*, 2n = 24; Figure 18A) and two eremobatids (*Eremobates pallipes* and *E. similis*, 2n = 22; Figure 18B and Figure S9A), were similar. They exhibited a predominance of acrocentric chromosome pairs which decreased gradually in size (Figure 18A,B and Figure S9A). The karyotype of *A. mulaiki* was formed by ten acrocentric (nos 1, 2, 4–6, 8–12) and two subtelocentric pairs (nos 3, 7) (Figure 18A). The karyotype of *E. pallipes* consisted of eight acrocentric (nos 2–5, 8–11), two subtelocentric (nos 1, 7), and one metacentric (no. 6) pairs (Figure S9A). The karyotype of *E. similis* was acrocentric, except for a probable metacentric pair (no. 7) (Figure 18B). One chromosome pair of *Eremobates* included an NOR locus (Figure 19C and Figure S9A). In *E. pallipes*, this locus occupied most of an arm of the metacentric pair no. 6 (Figure S9A).

#### 3.5.2. Daesiidae

The three daesiids examined, *Gluvia dorsalis*, *Eberlanzia flava*, and *Gnosippus* sp., differed greatly in diploid number although belonging to the same subfamily (Gluviinae). The karyotype of *G. dorsalis* (2n = 10) consisted of acrocentric chromosome pairs. The first two chromosome pairs were much longer (26.6% and 25.8% of TCL) than the other pairs in the karyotype, which decreased gradually in size from 16.2% to 15.4% of TCL. Constitutive heterochromatin was restricted to centromeric regions (Figure 18C). The male karyotype of *E. flava* (2n = 22) consisted of acrocentric chromosome pairs, except for two metacentric (nos 1, 7) and two subtelocentric (nos 2, 3) pairs. The first chromosome pair was considerably longer (17% of TCL) than the other pairs in the karyotype, which decreased gradually in size from 11.9% to 5.5% of TCL. Centromeres were detected as DAPI-positive regions (Figure 19A) during mitotic prophase and prometaphase. The short arms of a long subtelocentric chromosome pair (no. 3) were terminated by an NOR locus (Figure 19A). The karyotype of *Gnosippus* sp. appeared to display a predominance of acrocentric chromosomes. It consisted of eight chromosome pairs, which decreased gradually in size (Figure S9B).

#### 3.5.3. Galeodidae and Rhagodidae

The karyotypes of the galeodid, *Paragaleodes pallidus*, and rhagodid, *Rhagodes* sp., were unique in having a predominance of metacentric chromosome pairs (Figure 18D,F) and in the presence of a large amount of CH, which formed approximately half of the TCL during the mitotic metaphase. The karyotype of *P. pallidus* (2n = 12) comprised metacentric chromosome pairs which differed only slightly in length (Figure 18D). Both arms of each pair terminated by a large block of CH. The centromeres contained one or two tiny blocks of CH (Figure 18E) which were DAPI-positive (Figure 19B). The karyotype contained five NOR-bearing pairs; three included an NOR locus at both arms. Notably, the NOR loci occurred only in distal heterochromatic blocks forming tiny euchromatic regions embedded into the CH (Figure 18E and Figure 19B). The karyotype of *Rhagodes* sp. (2n = 18) was formed by metacentric chromosome pairs, except for two subtelocentric pairs (nos 6, 8). The chromosome pairs decreased gradually in size (Figure 18F). Most pairs had a large block of CH, which included the centromere and surrounding regions, or the centromere and one of the arms (Figure 18G).

#### 3.5.4. Solpugidae

The karyotype of the solpugid examined, a female *Solpugista* sp. (2n = 20), comprised acrocentric chromosome pairs. The first two chromosome pairs were much longer (19.8% and 15.2% of TCL) than the other pairs, which decreased gradually in size (from 10.8 to 5.3% of TCL) (Figure 18H). The chromosomes of a short pair involved an NOR. They differed in the size of the NOR (Figure 19D).

## 4. Discussion

### 4.1. Evolution of Cytogenetic Characters

#### 4.1.1. Diploid Numbers and Chromosome Morphology

##### Arachnid Orders

Most arachnids have standard (i.e., monocentric) chromosomes. Among tetrapulmonates, monocentric chromosomes are present in amblypygids [60,61,62,71] (present study), schizomids [66,71] (present study), thelyphonids [64,65,71] (present study), and most spiders except for synspermiate araneomorphs of the superfamily Dysderoidea which possess holocentric chromosomes [53,54]. The present study demonstrated that ricinuleids and solifuges also have monocentric chromosomes. All species analyzed in the study were karyotyped for the first time except the two amblypygids, *Paraphrynus mexicanus* [60] and *Damon medius* [67], and the solifuge, *Rhagodes* sp. [67]. The results confirmed published data on their diploid number and chromosome morphology, respectively.

Except for solifuges, a mesodiverse order, the orders investigated in the present study are among the less speciose terrestrial arthropod orders [2]. In amblypygids and thelyphonids, high karyotype diversity includes a large range of diploid numbers (22–86 in amblypygids, 28–78 in thelyphonids) and substantial generic-level karyotype differentiation [60,62] (present study). Karyotype data could therefore be an effective tool to differentiate between closely related species within these morphologically conservative orders. Data for the other three orders examined (Ricinulei, Schizomida, Solifugae) are insufficient to evaluate the generic level of karyotype diversity. Considerable intrageneric differentiation of karyotypes that is large enough for use in cytotaxonomy and phylogenetic reconstructions has been reported in most other arachnid taxa thus far analyzed cytogenetically, namely acariform and parasitiform mites [15,80], harvestmen [16,81], palpigrades [71], pseudoscorpions [18,82], scorpions [17,83], mygalomorph and synspermiate spiders [53,55,73] as well as some clades of entelegyne spiders (e.g., [84]).

##### Amblypygi and Thelyphonida

**Figure 20:**
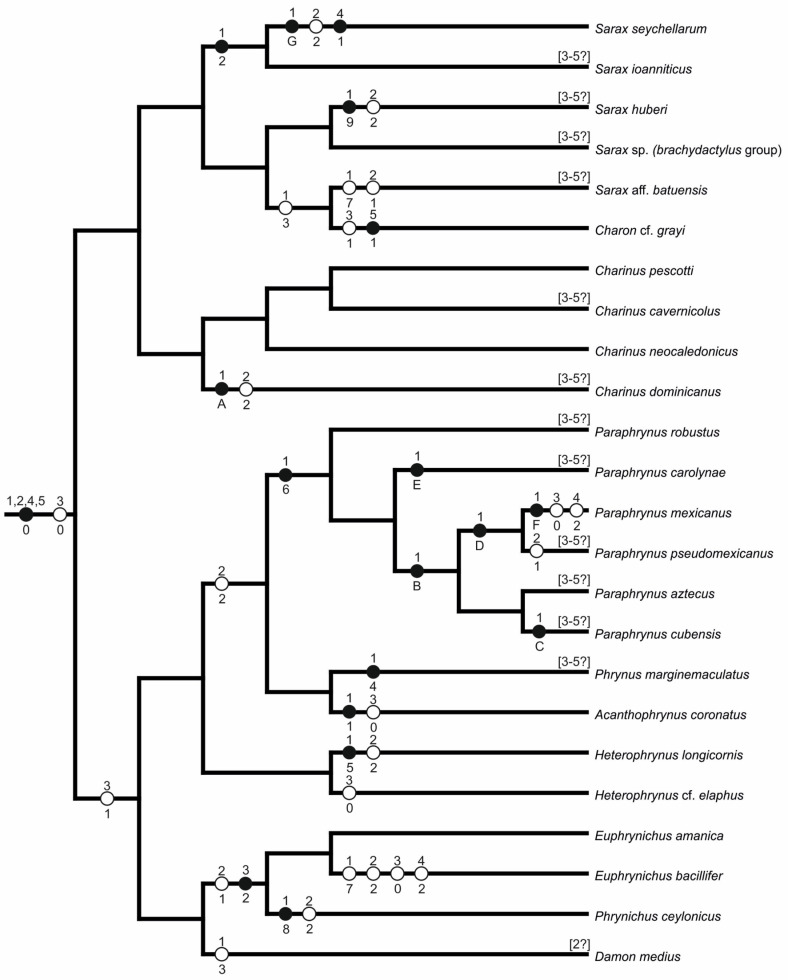
Hypothesis of karyotype evolution in order Amblypygi. Tree topology based on recent molecular phylogenies [24,85]. Karyotype data for 24 species in three families (see Table 1 and Table 2). Character numbers and states above and below circles, respectively. Black and white circles denote hypothesized unique and non-unique synapomorphies, respectively. Data missing for some species indicated in square brackets.

Despite their wide range of diploid numbers, most amblypygids and thelyphonids have comparatively large numbers of chromosomes (2n ≥ 40). A hypothesis of karyotype evolution for amblypygids is presented in Figure 20 (see Table 2 for distribution of cytogenetic characters scored). The ancestral amblypygid karyotype was probably similar to that of the early diverging amblypygid family Charontidae. According to this hypothesis, the ancestral karyotype of charontids had a high 2n (74–76 chromosomes) as well as a predominance of monoarmed chromosomes, with biarmed pairs comprising approximately one-third of the complement. This karyotype structure was found in the charontid genera *Charinus* and *Sarax* [62]. Moreover, similar karyotypes were found in the superfamily Phrynoidea, but with a slightly higher proportion of biarmed pairs [present study]. It may therefore be hypothesized that the ancestral karyotype of Phrynoidea contained more biarmed pairs than the ancestral amblypygid karyotype.

The ancestral karyotypes of spiders and thelyphonids were probably similar to the hypothesized ancestral karyotype of amblypygids. The karyotypes of the earliest-diverging spiders, the Mesothelae, also have high numbers of chromosomes (2n♂ = 71–ca. 96), most of which are monoarmed [86,87]. Likewise, the karyotypes of those thelyphonids with the highest chromosome counts (*Labochirus*, 2n = 78; *Uroproctus*, 2n = 72) are also similar to the hypothesized ancestral amblypygid, in both diploid number and the ratio of monoarmed to biarmed pairs, but these thelyphonid karyotypes are further characterized by the presence of several tiny chromosome pairs. Comparison of amblypygid and thelyphonid data suggest that the ancestral thelyphonid diploid number was close to that observed in *Uroproctus*. Perhaps unsurprisingly, *Uroproctus* also has the most ancestral morphology among Old World thelyphonids [88]. Although those thelyphonids with the hypothetical ancestral karyotype belong to different subfamilies, both are endemic to the Indian subcontinent [19]. This might suggest that thelyphonids evolved in Gondwana and/or that India is a refugium for these ancient lineages. The low diploid number found in New World thelyphonids (*Mastigoproctus*, *Yekuana*) is probably a derived feature within the order.

Amblypygid karyotype evolution probably included pericentric inversions [60,62] (present study), and other events that change chromosome morphology by shifting the centromere while the chromosome number remains unchanged (centromere repositioning and some types of translocations). Large centromere shifts, which seem to be common in amblypygids, are most likely the result of inversions. Centromere repositioning seems to be rather accompanied by only a small shift in the centromere position [89]. Translocation of a large segment can substantially change the position of the centromere. However, such translocations would often result in chromosomes that are significantly larger or smaller than other chromosomes, which has not yet been observed in amblypygids.

Among Phrynoidea, pericentric inversions and other mechanisms of centromere shift mentioned above likely played a role both in the formation of the ancestral karyotype of this superfamily, and in the subsequent karyotype evolution within this clade. These events are often involved in the formation of interspecific reproductive barriers (e.g., [90,91,92,93]) and might thus have contributed to speciation within the Phrynoidea, comprising Phrynidae and Phrynichidae.

The most ancestral karyotypes within Phrynoidea are hypothesized to be those of *Euphrynichus amanica* (2n = 78, Phrynichidae) and *Heterophrynus elaphus* (2n = 76, Phrynidae). They differ from the hypothetical ancestral amblypygid karyotype only in possessing a higher ratio of biarmed pairs, from which it may be hypothesized that the ancestral phrynoid karyotype evolved from the hypothetical ancestral amblypygid karyotype through pericentric inversions of some monoarmed pairs. Centromere repositioning and translocations could also be involved in this process. Although they belong to different families, *E. amanica* and *H. elaphus* have very similar diploid numbers and karyotype composition. This includes a similar placement of biarmed pairs in the karyotype, indicating homeology of most of these pairs. Therefore, these karyotypes probably reflect the plesiomorphic karyotypic structure within Phrynoidea. This hypothesis is supported by the basal positions of *Heterophrynus* and *Euphrynichus* within Phrynidae and Phrynichidae, respectively [24]. Paula-Neto et al. [61] hypothesized that the ancestral karyotype of Phrynoidea comprised a high diploid number. This hypothesis could be refined based on *Heterophrynus* and *Euphrynichus* data presented herein, which suggest that the ancestral Phrynoidea possessed 76–78 chromosomes, including 17–19 biarmed pairs. It may be further hypothesized that the transformation of monoarmed pairs into biarmed pairs through centromeric shift continued in *Acanthophrynus* (Phrynidae) which have a high portion (63%) of biarmed chromosomes. *Acanthophrynus* also has a higher diploid number (2n = 86) than the hypothesized ancestral phrynoid karyotype, probably due to the fission of some biarmed pairs to form monoarmed pairs. This hypothesis is supported by the small size of most monoarmed pairs in *Acanthophrynus*.

Pericentric inversions probably also played a role during karyotype evolution of the family Charontidae. Although the karyotype of *Charon* is close to the hypothetical ancestral amblypygid karyotype in diploid number (2n = 70), it has a lower ratio of biarmed to monoarmed pairs (20% of biarmed pairs). It is hypothesized that this karyotype evolved from a set with a higher ratio of biarmed pairs through pericentric inversions [present study]. This hypothesis is based on an unusual chiasma pattern during male meiosis of *Charon*–bivalents composed of monoarmed chromosomes usually form only a single chiasma [94] but the monoarmed pairs of *Charon* males frequently formed two chiasmata. Previous studies (e.g., [95]) showed that inverted chromosome regions often retain their original chiasma pattern. The two chiasmata in the monoarmed pairs of *Charon* could thus be explained by inversions of some biarmed pairs that retained their chiasmata.

Another major trend in amblypygid karyotype evolution is toward gradual reduction in 2n, accompanied by an increase in the number of biarmed chromosomes. Most karyotypes with reduced 2n (<74) are mostly or completely biarmed [60,61,62] (present study). Amblypygid evolution probably included many independent events with considerable reduction of diploid numbers, mainly via a large number of centric fusions. Such events can form a very efficient reproductive barrier between species [96,97].

The hypothesized involvement of multiple fusions in reproductive isolation of amblypygids can be inferred from karyotype comparison among related species, such as in the genera *Euphrynichus* and *Heterophrynus*. Evolution of the karyotype of *E. bacillifer* (2n = 56; 24 biarmed pairs) from the karyotype of *E. amanica* (2n = 78; 19 biarmed pairs) included a series of centric fusions followed by several inversions. The karyotype of *H. longicornis* (2n = 66; 27 biarmed pairs [61]) probably arose from the *H. elaphus* karyotype (2n = 76; 17 biarmed pairs) via pericentric inversion of five monoarmed pairs and five centric fusions. The karyotype structure of *Phrynichus ceylonicus* (2n = 52, all pairs biarmed except for one monoarmed pair) is similar to a hypothetical karyotype derived from the ancestral phrynoid karyotype by centric fusions of all monoarmed pairs. The karyotype of two charontid species (*Sarax seychellarum*, 2n = 22 and *Charinus dominicanus*, 2n = 42) [62] and four species of the *Paraphrynus aztecus* group (2n = 24–36) [60] comprise entirely biarmed pairs. However, the diploid number of these species is too low to have arisen according to the scenario proposed for *Phrynichus*. The karyotypes of these amblypygids could be derived from the biarmed karyotype formed by fusion of all monoarmed pairs. Subsequent pericentric inversions would transform some biarmed pairs into monoarmed ones, which can fuse into biarmed pairs. This process would be accompanied by a reduction in the diploid number as observed in *Paraphrynus mexicanus* of the *P. aztecus* group [60]. The formation of biarmed karyotypes in amblypygids with low 2n could also involve other rearrangements forming biarmed chromosomes, for example nested fusions (mechanism reviewed by Lysak et al. [98]).

Thelyphonid karyotype evolution probably also involved frequent pericentric inversions and reductions of diploid numbers. Chromosome numbers seem to have decreased independently in each of the four thelyphonid subfamilies. Thelyphonids with reduced diploid numbers can be divided into two groups. The karyotypes of the first group possess slightly reduced diploid numbers and a predominance of biarmed pairs. Two species examined fall into this group, namely the hypoctonine *Hypoctonus* cf. *gastrostictus* (2n = 66, 22 biarmed pairs) and the thelyphonine *Thelyphonus* cf. *linganus* (2n = 66, 20 biarmed pairs). Reduced diploid numbers and the prevalence of biarmed pairs might suggest these karyotypes evolved from an ancestral thelyphonid karyotype through centric fusions, but this would have resulted in fewer biarmed pairs than are currently observed, especially in *Hypoctonus*. It is thus hypothesized that centric fusions were accompanied by pericentric inversions of some monoarmed pairs, which increased the proportion of biarmed chromosomes.

The karyotypes of the second group have much reduced diploid numbers and contain approximately the same proportion of biarmed and monoarmed pairs or are predominated by monoarmed pairs. *Mastigoproctus giganteus* can also be included in this group; its karyotype is only slightly predominated by biarmed pairs. Representatives of the second group were found in all thelyphonid subfamilies. *Thelyphonus sepiaris*, for example, differed considerably from *T.* cf. *linganus* both in diploid number (2n = 42 [65]; 2n = 44 [64]) and the morphology of the chromosomes, which were reported to be mostly [65] or entirely monoarmed [64]. Based on the high number of monoarmed pairs which form two chiasmata during male meiosis in *T. sepiaris* (see Figures 6 and 7 in [65]), it is hypothesized that the ancestral *Thelyphonus* karyotype was predominantly biarmed, similar to that of *T.* cf. *linganus*, and that the number of biarmed pairs was subsequently reduced through pericentric inversions during the evolution of *T. sepiaris*. As in *T. sepiaris*, most other species in the second group possess a high number of monoarmed pairs forming two chiasmata, which suggests a predominantly biarmed set in the ancestors of these species. The diploid number observed in *Typopeltis crucifer* (2n = 40) differs from the 2n = 12 reported by Warren [68]. The latter figure is, however, dubious as this author misinterpreted fundamental processes of chromosome division (cf. [99]). *Typopeltis guangxiensis* differs from *T. crucifer* in the slightly lower diploid number (2n = 36) and one small-sized chromosome pair. The latter could have arisen via non-reciprocal translocation of a large segment to another pair.

The distribution of cytogenetic characters scored for representatives of order Thelyphonida is provided in Table 3.

##### Schizomida

**Figure 21:**
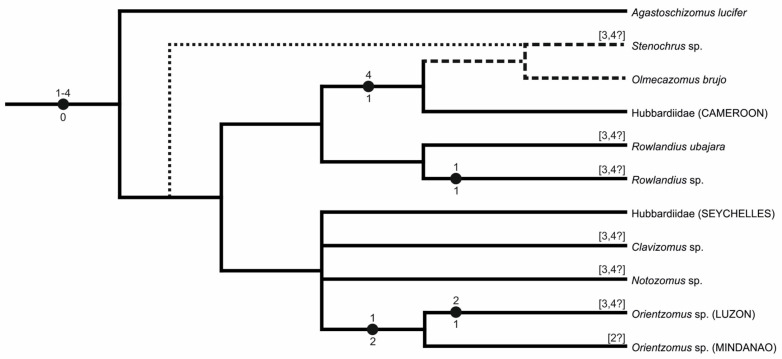
Hypothesis of karyotype evolution in order Schizomida. Tree topology based on recent molecular phylogeny [27] with phylogenetic positions of some species determined based on distributions and phylogenetic affinities, position of *Olmecazomus* determined based on karyotype data (dashed line), and alternative phylogenetic positions of *Stenochrus* indicated by dashed and dotted lines. Karyotype data for eleven species representing both families (see Table 1 and Table 4). Character numbers and states above and below circles, respectively. Black and white circles denote hypothesized unique and non-unique synapomorphies respectively. Data missing for some species indicated in square brackets.

Schizomids have much lower diploid numbers than amblypygids and thelyphonids, ranging from 16 to 22 chromosomes. Schizomid karyotypes are relatively conserved, which facilitates a reconstruction of their ancestral features. A hypothesis of karyotype evolution in schizomids is presented in Figure 21 (see Table 4 for distribution of cytogenetic characters scored). The ancestral schizomid karyotype probably comprised eleven acrocentric pairs, which gradually decreased in size. This karyotype structure was observed in representatives of the two primary lineages of schizomids, i.e., *Olmecazomus* (Hubbardiidae), two undetermined hubbardiids, and *Agastoschizomus* (Protoschizomidae). A very similar karyotype was observed in *Notozomus* and *Stenochrus*. In contrast to the previous species, some pairs were subtelocentric, a morphology that might have arisen by pericentric inversion. Alternatively, the short arms of subtelocentric chromosomes could have arisen by the accumulation of constitutive heterochromatin. Accumulation of this type of chromatin in the short arms of monoarmed chromosomes is a common phenomenon that has been found, for example, in some mammals [100,101,102].

The hubbardiid *Clavizomus* has the same 2n as the hypothetical ancestral schizomid but differs from the latter in two respects. First, the chromosome pairs decrease in size discontinuously, which could be the result of non-reciprocal translocations. Second, the smallest pair is biarmed, but this morphology could not have arisen through centric fusion as the diploid number of *Clavizomus* is not reduced. It is therefore hypothesized that this biarmed morphology formed through pericentric inversion. The hubbardiid *Rowlandius ubajara* has the same karyotypic structure as *Clavizomus* (2n = 22, all chromosomes monoarmed except for one biarmed pair) [66]. Therefore, the biarmed pair of this schizomid probably also originated through pericentric inversion. The karyotype of *Rowlandius* sp. has one less chromosome pair than *R. ubajara*, and its biarmed chromosome pair is much larger than the biarmed pair of *R. ubajara* [66]. These features of the karyotype suggest that the single biarmed pair of *Rowlandius* sp. arose through centric fusion.

The apparently most derived schizomid karyotype was observed in the hubbardiid *Orientzomus*, which has a lower diploid number comprising eight chromosome pairs. The metacentric morphology of most pairs suggests a reduction of the 2n through centric fusions. Three centric fusions are necessary to reduce the hypothetical ancestral schizomid set (i.e., eleven monoarmed pairs) to eight pairs. The number of metacentric pairs in *Orientzomus* is higher (comprising five pairs) than expected to be produced by fusions, thus indicating that some metacentric pairs might have arisen through pericentric inversion.

##### Ricinulei

**Figure 22:**
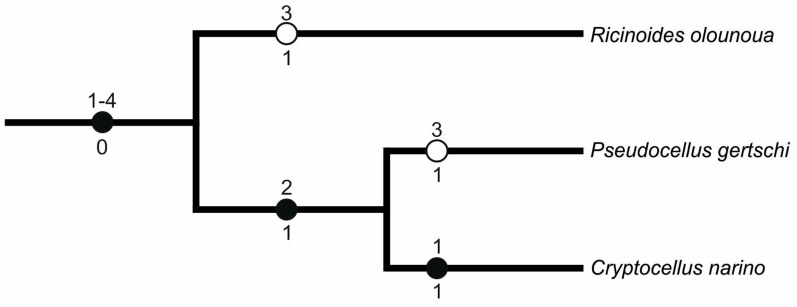
Hypothesis of karyotype evolution in order Ricinulei. Tree topology based on recent molecular phylogeny [42]. Karyotype data for three species representing all genera (see Table 1 and Table 5). Character numbers and states above and below circles, respectively. Black and white circles denote hypothesized unique and non-unique synapomorphies, respectively.

Ricinuleids have relatively high chromosome numbers (2n = 40–46). Based on phylogenetic data, extant ricinuleids comprise two sister clades, an African clade (*Ricinoides*) and a New World clade, comprising *Cryptocellus* and *Pseudocellus* [42]. Although these clades separated during the Mesozoic before the breakup of Gondwana, their karyotypes remain similar, which indicate a slow karyotype evolution of ricinuleids (see Figure 22 for a hypothesis of karyotype evolution in ricinuleids and Table 5 for distribution of cytogenetic characters scored). The ancestral karyotype of ricinuleids probably comprised 40 chromosomes that were predominantly biarmed as in *Ricinoides.* The proportion of monoarmed chromosomes subsequently increased by pericentric inversions in the New World clade. The number of monoarmed pairs further increased in *Cryptocellus*, accompanied by an increase in the diploid number. This pattern suggests the occurrence of chromosome fissions during the evolution of this genus. The prominent, large pairs observed in ricinuleids could arise by non-reciprocal translocations.

##### Solifugae

**Figure 23:**
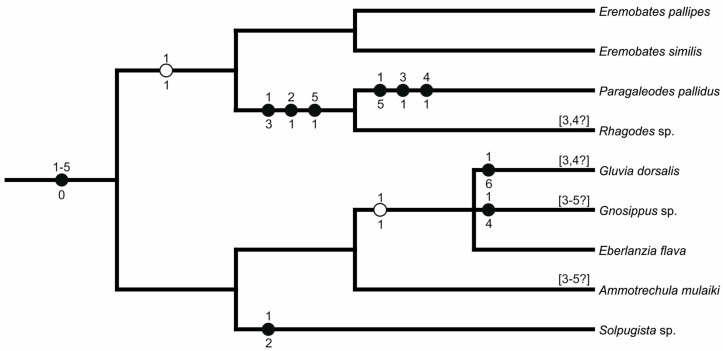
Hypothesis of karyotype evolution in order Solifugae. Tree topology based on recent molecular phylogeny [52]. Karyotype data for nine species in six families (see Table 1 and Table 6). Character numbers and states above and below circles, respectively. Black and white circles denote hypothesized unique and non-unique synapomorphies, respectively. Data missing for some species indicated in square brackets.

Solifuges have low diploid numbers, ranging from 10 to 24 chromosomes. A hypothesis of karyotype evolution in solifuges is presented in Figure 23 (see Table 6 for distribution of cytogenetic characters scored). The ancestral solifuge karyotype probably comprised twelve chromosome pairs, which decreased gradually in size, and were mostly or exclusively acrocentric. This karyotype and a closely related karyotype formed by eleven pairs were observed in families Ammotrechidae, Daesiidae, and Eremobatidae, which represent both suborders of solifuges, Australosolifugae and Boreosolifugae. Although molecular markers suggest a deep divergence between ammotrechids and eremobatids (during the Upper Paleozoic) [52,103,104], similarity of their karyotypes [present study] and mitochondrial genomes [103] implies that these genome characteristics evolved slowly in the two families. The species of *Eremobates* examined, *E. pallipes* and *E. similis*, exhibit the same diploid number and chromosome morphology except for the morphology of two pairs. These differences could reflect the occurrence of pericentric inversions or translocations during the evolution of the genus.

**Figure 24:**
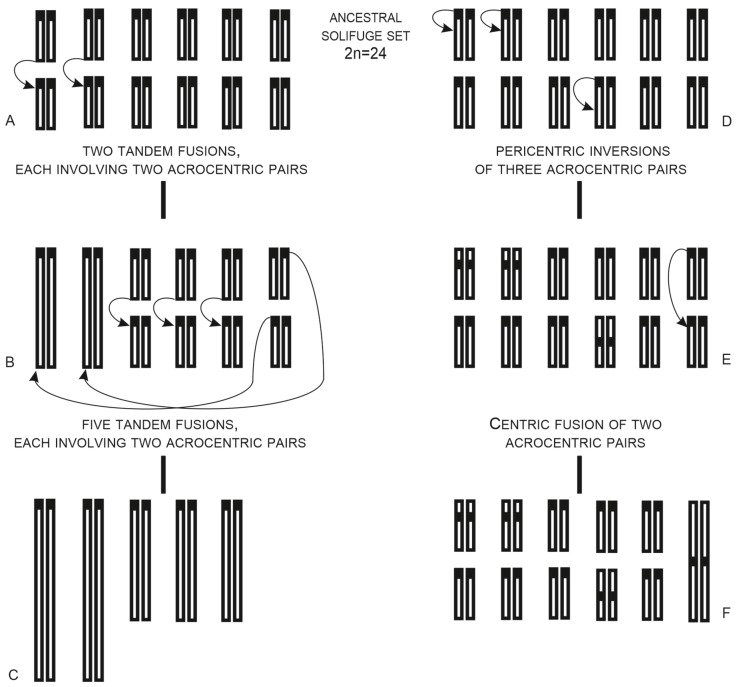
Hypotheses of karyotype evolution in selected Solifugae. Reconstruction of karyotype evolution based on hypothetical ancestral solifuge karyotype, comprising twelve acrocentric chromosome pairs, which enables most parsimonious derivation of all known solifuge karyotypes. (**A**–**C**) Left branch: operation of tandem fusions (arrows) during solifuge evolution; hypothesized formation of karyotypes of *Solpugista* (2n = 20) (**B**) and *Gluvia* (2n = 10) (**C**) from ancestral solifuge karyotype (**A**). Karyotype of *Solpugista* (**B**) representing an intermediate stage to karyotype of *Gluvia* (**C**). (**D**–**F**) Right branch: hypothesized formation of *Eberlanzia* karyotype (2n = 22) (**F**) from ancestral solifuge karyotype through three pericentric inversions (three arrows) (**D**), followed by centric fusion (arrow) (**E**).

It is hypothesized that the diploid numbers of solifuges were often reduced, notably in families Daesiidae, Galeodidae, Rhagodidae, and Solpugidae. In three solifuges examined (*Gluvia*, *Gnosippus*, and *Solpugista*), the diploid number was lower than the hypothetical ancestral solifuge number and the chromosome morphology monoarmed. Reduction of the diploid number in these solifuges suggests that their karyotype evolution involved fusions. Two of the chromosome pairs were much longer than the others in *Gluvia* and *Solpugista*. Karyotypes of these solifuges evolved independently. The simplest way to derive them from the ancestral solifuge karyotype is by tandem fusions (Figure 24). The karyotype of *Solpugista* (Solpugidae) has a slightly reduced diploid number (20) compared to the ancestral solifuge karyotype. Each of the two large chromosome pairs of *Solpugista* probably arose through tandem fusion of two chromosome pairs (Figure 24). The karyotype of *Gluvia* (Daesiidae) (2n = 10) could have evolved from a karyotype resembling that of *Solpugista*, in which each of the ten chromosome pairs arose through tandem fusion of two chromosome pairs (Figure 24). Unlike *Gluvia* and *Solpugista*, the karyotype of *Eberlanzia* (2n = 22) (Daesiidae) possesses only a single prominent chromosome pair which, moreover, is metacentric. *Eberlanzia* also exhibits a higher diploid number than the former genera, which is close to the hypothetical ancestral solifuge number. The prominent metacentric pair probably originated through centric fusion. Other non-acrocentric pairs of this species probably arose through pericentric inversion (Figure 24). Compared to other solifuges, the karyotypes of galeodids and rhagodids possess a predominance of biarmed chromosomes. These karyotypes could have been derived from the ancestral karyotype of solifuges through centric fusions of all (*Paragaleodes*) or most (*Rhagodes*) pairs.

#### 4.1.2. Somatic Pairing of Chromosomes

Somatic pairing occurs in some eukaryotes and refers to the association formed between homologous chromosomes during mitosis or even during most of the cell cycle [105]. Surprisingly, evidence for somatic pairing in arachnids was found in the present study. Among solifuges and some Pedipalpi (Amblypygi and Thelyphonida) homologous chromosomes often formed loose associations during gonial and somatic mitosis. In solifuges, interphase nuclei often contained a haploid number of heterochromatic blocks, suggesting close contact between homologs during interphase. This pattern was not observed in all nuclei, however. Therefore, the association between homologs is probably restricted to a specific period of interphase.

Somatic pairing is probably more common in Pedipalpi than the results suggest, as it is difficult to detect somatic pairing in these arachnids, for various reasons. Firstly, Pedipalpi chromosomes usually do not contain large blocks of CH hindering the detection of chromosome associations during interphase. Secondly, homologs form very loose associations during mitosis in Pedipalpi. This, combined with the observation that associations are often found only in some plates, suggests that associations of somatic chromosomes were not detected up to now due to their instability. This is corroborated by observations that the associations were better preserved when hypotonization time was reduced, preventing overspreading of the chromosome plates. Thirdly, it is often difficult to identify homologs in a Pedipalpi plate due to high diploid numbers and/or indistinct primary constrictions.

#### 4.1.3. Constitutive Heterochromatin

Constitutive heterochromatin has thus far been detected in seven arachnid orders, namely amblypygids [61,62], harvestmen [106], palpigrades [71], parasitiform mites [107,108], scorpions (see [109] for review), spiders (see [54] for review), and thelyphonids [65]. The present study considerably increased the knowledge of CH in amblypygids and thelyphonids, and provides the first data on CH in ricinuleids, schizomids, and solifuges. These findings, together with data available in the literature, suggest that most arachnids have a low to moderate amount of CH which is mostly located in the centromeric and telomeric regions. Centromeric CH is predominantly AT-rich in most species examined. A low to moderate CH, located in the centromeric and telomeric regions, and predominantly AT-rich centromeric CH, could be ancestral for arachnids.

Unlike other arachnids, two families of solifuges, the rhagodids and galeodids, possess an enormous amount of CH, which constitutes approximately half the total chromosome length of the diploid set. Comparable amounts of CH are known to exist in only a few animal taxa, for example diplopods [110] and some clades of Hymenoptera [111]. Although the karyotypes of rhagodids and galeodids are similar in terms of the predominance of biarmed chromosomes, they differ in their CH patterns. The expansion of CH in these solifuges could have several functions. Firstly, expanded CH could contribute to reproductive isolation. Heterochromatin can be a rapidly evolving reproductive barrier [112]. Expansion of CH could further accelerate the formation of reproductive barriers due to a faster generation of species-specific CH patterns [113]. Secondly, the expanded CH could facilitate somatic pairing. Constitutive heterochromatin can promote meiotic pairing of homologs in the absence of chiasmata [114]. It is therefore likely that CH also takes part in somatic pairing, and that the expansion of CH could strengthen this pairing. Thirdly, the associated homologous blocks of expanded CH could form an environment conducive to the formation and function of nucleoli. This hypothesis is supported by the restriction of galeodid NORs to enormous distal blocks of CH (see section below). As NORs can also take part in achiasmatic pairing [114], it is tempting to hypothesize that galeodid NORs strengthen somatic pairing of homologous chromosomes along with large blocks of CH.

#### 4.1.4. Nucleolar Organizer Regions

Nucleolar organizer regions have thus far been studied in seven arachnid orders, namely amblypygids [61,62], harvestmen (e.g., [115,116,117,118,119,120]), palpigrades [71], parasitiform mites [107,121,122], pseudoscorpions (e.g., [123]), scorpions (e.g., [83,124,125,126,127,128]), and spiders (e.g., [55,57,59,70,73,84,129,130,131,132,133]). Except for harvestmen, scorpions, and spiders, data on NORs are limited because few species have been investigated. Most arachnids have a low number of NOR loci. The karyotypes of ancestral arachnids probably contained a single NOR locus [59]. NORs are usually located at the ends of chromosome arms in arachnids, which is probably also an ancestral feature of these arthropods [61]. Arachnids only have autosome NORs except for some ticks [122] and spiders (e.g., [57,73,132,133]) which also exhibit sex chromosome-linked NORs. In many arachnids, NORs have been detected through silver staining. As this technique only visualizes NORs that are transcriptionally active during the preceding interphase [134], FISH was used in the present study, as it permits visualization of both active and inactive NORs. The present study outlines the first NOR data for four arachnid orders, namely Ricinulei, Schizomida, Solifugae, and Thelyphonida.

The ricinuleids and schizomids examined possess a single NOR locus, in accordance with the hypothetical ancestral arachnid pattern. The NOR locus of ricinuleids is subterminal or terminal. The NOR locus of schizomids is terminal or interstitial. The latter location is less common in arachnids, and probably arose through the inversion of a terminal NOR locus. An interstitial NOR, only observed in Mexican and West African hubbardiids, is probably a synapomorphy of these clades (Figure 21).

A single NOR locus is also hypothesized to be ancestral for solifuges [present study]. This pattern was observed in all solifuges that exhibit a presumed ancestral pattern of chromosome morphology, i.e., all or almost all pairs acrocentric. The number of NORs was considerably larger in the biarmed karyotypes of galeodids with one, or sometimes two, subterminal NOR loci present on most of the chromosome pairs.

Amblypygid karyotypes possess one to three NOR loci. Data from early diverging clades (i.e., Paracharontidae, Charontidae) could provide insight into the ancestral NOR pattern of amblypygids. Although data from paracharontids are missing, representatives of Charontidae exhibited one (all examined representatives of *Charinus* and *Sarax*) [62] or two (*Charon*) [present study] NOR loci. Whereas the NORs of *Charinus* and *Charon* were situated at the end of the chromosome [62] (present study), the NOR of a single species of *Sarax* examined was interstitial, probably arising from a paracentric inversion of a terminal NOR [62]. The phylogenetic distribution of charontids with a single NOR (Figure 20) suggests that this pattern is ancestral for this early diverging clade and therefore also for Amblypygi.

The ancestral karyotype of Phrynoidea probably possessed two NOR loci, based on two lines of evidence. Firstly, species with two NOR loci occur in both families of this clade, namely Phrynidae [61] and Phrynichidae [present study]. Secondly, the diploid numbers of species with two NOR loci are close to the range of the hypothetical ancestral amblypygid karyotype.

The data presented herein imply frequent changes in NOR patterns during the evolution of Phrynoidea. It is hypothesized that the number of NOR loci was often reduced to a single locus in this clade, i.e., three times in phrynids and once in phrynichids, probably by loss or fusion of these structures. Among phrynids, the hypothetical ancestral number of NORs (i.e., two loci) was only observed in *Heterophrynus longicornis* (Heterophryninae) [61]. The karyotype of *H. elaphus* contained a single NOR locus with the second NOR locus presumably lost. The ancestral NOR pattern of Phryninae probably consisted of two subtelocentric chromosome pairs, each with an NOR locus terminating the short arm. The present study included two genera of Phryninae, *Acanthophrynus* and *Paraphrynus*. The karyotype of *Acanthophrynus* contained a single NOR-bearing pair, which retains the ancestral chromosome morphology and the location of the NOR locus. The second NOR locus has been lost. *Paraphrynus* also possessed a single NOR-bearing pair. However, this pair differed from that of *Acanthophrynus* in its metacentric morphology and in the pericentric location of the NOR locus. This pattern probably originated through centric fusion of the ancestral subtelocentric pairs of Phryninae, each with an NOR locus terminating the short arm.

Within family Phrynichidae, the number of NOR loci was apparently reduced in *Euphrynichus bacillifer.* Interestingly, the single NOR-bearing chromosome pair of this species had the same morphology and location of the NOR locus as *Paraphrynus*. The data presented herein suggest that the pericentric NOR locus of *E. bacillifer* arose in a similar manner to that of *Paraphrynus*, i.e., through centric fusion of two monoarmed chromosome pairs with an NOR locus at the end of the short arm. Two such putative chromosome pairs were observed in *E. amanica*. In addition to these two pairs, the karyotype of *E. amanica* also included a metacentric pair terminated by an NOR locus. This locus was absent, presumably lost, in *E. bacillifer*. A frequent occurrence of centric fusions during the evolution of *P. mexicanus* and *E. bacillifer* is suggested by a considerable decrease in their diploid number accompanied by a conversion of all (*P. mexicanus*) or most (*E. bacillifer*) monoarmed pairs into biarmed pairs.

The number of NOR loci increased to three in the phrynichids, *E. amanica* and *Phrynichus ceylonicus*. *Euphrynichus* and *Phrynichus* form a separate clade [24], for which three NOR loci could be a synapomorphy.

Considerable diversification of the NOR pattern was observed among thelyphonids, with the number of NOR loci ranging from one to five. Comparison of the species examined suggests that the ancestral thelyphonid karyotype probably contained two or three terminal NOR loci. Although the NOR-bearing chromosome pairs were exclusively biarmed in most species examined, the ancestral morphology of these pairs in thelyphonids remains unclear. No NOR data are available for taxa that exhibit the hypothetical ancestral karyotype of the order, i.e., predominantly acrocentric. The NOR pattern of *Yekuana* (Hypoctoninae) and *Typopeltis* (Typopeltinae) is presumably derived. These genera also possess low diploid numbers, hypothesized to be derived in thelyphonids. Derived karyotype features are also consistent with the derived morphology of these genera [88]. A reduction in the diploid number in *Yekuana* was probably accompanied by a decrease in the number of NOR loci to a single locus. Despite the reduced chromosome number, the number of NOR loci increased in *Typopeltis*, which has four chromosome pairs terminating by an NOR locus, with one metacentric pair even possessing two NOR loci. Some specimens of *Typopeltis* exhibited NOR polymorphism being heterozygous for the presence of an NOR at one locus. This pattern could arise through hybridization between individuals that differed in NOR genotype within this locus.

In the present study, NOR polymorphisms were also observed in several other arachnids. In the amblypygid *Charon*, the data suggest a sex-linked polymorphism of the NOR locus with males being heterozygous for the presence of an NOR (see section on sex chromosomes). NOR size polymorphisms were detected in the ricinuleid, *Pseudocellus*, and in the solifuge, *Solpugista*. NOR polymorphisms are relatively common in animal populations (e.g., [59,135,136]).

#### 4.1.5. Telomeric Repeats

Eukaryotic DNA is terminated by non-coding telomere repeats with a highly conserved sequence [137]. This sequence is therefore a suitable marker for higher level phylogeny. Vítková et al. [67] determined the ancestral telomere motif (TTAGG)_n_ of arthropods, including arachnids, and analyzed its evolution. These authors examined nine arachnid orders, including two tetrapulmonate orders (spiders, amblypygids) and solifuges, and found that all arachnids, except spiders, exhibit the arthropod motif. Reyes Lerma et al. [62] confirmed this motif in amblypygids. In the present study, the evolution of telomere arrays was traced in tetrapulmonates and their putative relatives. The arthropod motif was reconfirmed in amblypygids and found in representatives of the other orders of Pedipalpi (Schizomida, Thelyphonida) and in Ricinulei. Loss of the arthropod motif can therefore be considered an autapomorphy for spiders.

#### 4.1.6. Sex Chromosomes

Sex chromosomes were probably either absent or homomorphic ancestrally in arachnids [71]. This hypothesis is supported by the recent finding of homomorphic sex chromosomes in buthid scorpions [125]. Heteromorphic (i.e., morphologically differentiated) sex chromosomes only evolved in some arachnid clades. They have been revealed in pseudoscorpions [82], spiders [54,138], most ticks [80,139], some clades of acariform mites [15], and harvestmen [81].

In the present study, CGH was used to detect sex chromosomes that show a sufficient degree of sequence differentiation. Among arachnids, this approach has thus far been applied only to spiders [58]. Using CGH, it was possible, for the first time, to demonstrate the presence of sex chromosomes along with a putative male-specific region (MSY) in amblypygids, namely an XY system in *Paraphrynus mexicanus* (Phrynidae). The biarmed X and Y chromosomes of *Paraphrynus* differed slightly in morphology, specifically in the size and position of the centromere. The MSY was located at the end of the short arm of the Y chromosome. The results also suggested that an XY system is present in *Charon grayi* (Charontidae). The putative sex chromosomes of *Charon* were acrocentric, and the Y chromosome differed from the X chromosome by a reduction of the centromeric CH and the absence of NOR. These differences probably reflect an initial differentiation of the sex chromosomes. Nevertheless, it was not possible to detect an MSY in this species using CGH. The absence of a CGH signal could be because the MSY is small and thus below the detection limit of CGH, or due to insufficient differences in the amount and composition of accumulated repetitive DNA between sexes which in turn would result in the lack of sex-specific signal [140,141]. Lastly, the MSY might have been situated close to the centromere region (see below) which would make it difficult to differentiate from the bright fluorescence of this chromosome region. A combination of the above reasons is also possible.

The presence of sex chromosomes in amblypygids has been previously indicated by a specific behavior of a chromosome pair in the oogonial mitoses of charontid *Sarax ioanniticus* [62]. Other reports on amblypygid sex chromosomes are almost certainly misinterpretations. In one study, Millot and Tuzet [63] described a single male sex chromosome (i.e., an X0 system) in a species of *Sarax* (Charontidae). However, the karyotype of the other *Sarax* species examined did not contain heteromorphic sex chromosomes, nor an X0 system [62]. In another study, Paula-Neto et al. [61] reported that homomorphic sex chromosomes are present in *Heterophrynus longicornis* (Phrynidae). Their assumption was based on the absence of heteromorphic sex chromosomes in this taxon. However, the absence of heteromorphic chromosomes does not imply the presence of homomorphic sex chromosomes.

Data on the presence of sex chromosomes in thelyphonids are contradictory. For *Thelyphonus sepiaris*, Millot and Tuzet [63] reported a single sex chromosome, Kasturi Bai and Parthasarathy [64] found heteromorphic X and Y chromosomes, and Jayarama and Gowda [65] did not find any sex chromosomes. Data on *Thelyphonus* cf. *linganus* presented herein support the presence of an XY system in *Thelyphonus*. As with the findings of Kasturi Bai and Parthasarathy [64] on *T. sepiaris*, a considerable difference was observed in the size of the sex chromosomes of *T.* cf. *linganus*. The contradictory data on sex chromosomes in *T. sepiaris* may be due to taxonomic misidentification, a rapid size reduction of the Y chromosome due to its degeneration, or both. It is therefore possible that Kasturi Bai and Parthasarathy [64] and Jayarama and Gowda [65] studied different chromosome races, subspecies, or species, in which the sex chromosomes of the population studied by the latter authors had no, or only slight, morphological differentiation. These ideas are supported by the slightly different karyotype described by these authors. However, it is unlikely that *T. sepiaris* includes both populations with a slight morphological differentiation of sex chromosomes and populations with a loss of the Y chromosome (i.e., formation of the X0 system). Therefore, the report of a single male sex chromosome in *T. sepiaris* [63] is most likely a misinterpretation. Remarkably, heteromorphic sex chromosomes were not found in another thelyphonine, *Ginosigma*. Therefore, within the Thelyphoninae examined to date, morphological differentiation of sex chromosomes seems restricted to *Thelyphonus*, but this might eventually prove to also be the case in other closely related genera. Members of the other thelyphonid subfamilies also did not have heteromorphic sex chromosomes.

The sex chromosome differentiation in *Thelyphonus* was not analyzed with CGH. However, this technique was used to detect sex chromosomes in *Typopeltis*, a thelyphonid without heteromorphic sex chromosomes [present study]. Although the results were negative, it remains possible that the set of *Typopeltis* contains proto-sex chromosomes, which cannot be detected by CGH.

Information about the sex chromosomes of Pedipalpi could be important to elucidate the origin of the peculiar sex chromosomes of spiders. The karyotypes of most spiders contain multiple X chromosomes [54,86,138]. Recent findings suggest that spiders also have a homomorphic sex chromosome pair comprising an X and Y chromosome, hypothesized to be the ancestral sex chromosome system of spiders [56]. This hypothesis is supported by the similarity between the spider homomorphic sex chromosome pair and the Pedipalpi X and Y sex chromosomes with their low morphological differentiation. This similarity could reflect a common origin of the Pedipalpi XY systems and the spider homomorphic sex chromosome pair. If so, the ancestor of amblypygids, thelyphonids, and spiders would have a homomorphic XY system. Such a system would have considerable antiquity as these groups can be traced to the Upper Carboniferous [142]. Multiple X chromosomes of spiders could originate from nondisjunctions of an X chromosome belonging to the homomorphic sex chromosome pair [56]. This scenario is supported by the unique behavior of multiple X chromosomes in the female germline of spiders [56,57].

The presumed antiquity of the homomorphic XY system in tetrapulmonates raises the question as to how these slightly differentiated sex chromosomes arose, and how they resisted the evolutionary forces responsible for degeneration of the Y chromosome. The formation of sex chromosomes is usually initiated by a suppression of recombination between major sex-determining genes [143]. The linkage of the MSY to the telomere region in the amblypygid *Paraphrynus* implies the involvement of a specific telomere structure in this process. According to Phillips and Ihssen [144], telomeric CH could promote the formation of MSY in the subtelomeric region through the suppression of recombinations in this part of the chromosome. The Y chromosome of *Paraphrynus* differs from the X chromosome in the location of the centromere and a slight reduction in size, which suggests deletion(s) of part of the Y chromosome. The different position of the Y centromere could also have occurred through pericentric inversion(s) of the X and/or Y chromosome. Deletions and inversions are often involved in sex chromosome differentiation [143,145]. Despite the structural differentiation of the Y chromosome, the MSY of *Paraphrynus* remains tiny and restricted to a telomere region. Expansion of the MSY may have been prevented by a recombination hotspot in the subtelomeric area of the Y chromosome. This hypothesis is supported by the distal location of most chiasmata during male meiosis of *Paraphrynus*. The same mechanism was suggested to be involved in preventing the expansion of MSY in tinamid birds [146]. The sex chromosome differentiation in *Paraphrynus* is not accompanied by an accumulation of CH; autosomes and sex chromosomes have a similar pattern and low amount of CH.

Although an MSY was not detected in the amblypygid *Charon*, it is possible to deduce its location. It is probably situated on the short arm of the putative acrocentric Y chromosome, i.e., close to the centromere. This arm differs from the corresponding region of the X chromosome by a reduction of centromeric CH and by the absence of an NOR. These structural changes could be involved in the Y chromosome differentiation of *Charon*. The evolution of a Y chromosome is often accompanied by an accumulation of heterochromatin [147]. However, evolution of the Y chromosome in some animals included the loss of specific heterochromatic regions [148]. The specific structure of an NOR could also play a role in sex chromosome evolution. The highly recombinogenic DNA of an NOR inhibits crossing over in the surrounding area via interference, which could promote formation of a sex determining region (SDR) close to the NOR [149]. According to the hypothesis presented herein, NORs could also be involved in the subsequent evolution of sex chromosomes. The recombination hotspot of an NOR could prevent the decay of the Y (or W) chromosome by forming a barrier to the expansion of an adjacent region without recombinations, which includes the SDR. The expansion of this region would only be possible after the relocation or elimination of this NOR. Consistent with this hypothesis, the Y (or W) chromosome-linked NOR is often reduced or even lost during the initial morphological differentiation of the sex chromosomes (e.g., [150,151,152], present study).

The most differentiated sex chromosomes of Pedipalpi were observed in *Thelyphonus*. The size of putative Y chromosome was distinctly reduced in *Thelyphonus* [64] (present study). Furthermore, the sex chromosomes exhibited a specific behavior during male meiosis in *T.* cf. *linganus*, which implies considerable structural differentiation. Firstly, the male sex chromosome bivalent contained a single chiasma confined to a specific region, which suggests that the other regions of the X and Y chromosomes already underwent a substantial sequence divergence and, consequently, they do not recombine. Secondly, the putative X chromosome was heterochromatic during some phases of male meiosis. The principal function of sex chromosome inactivation in meiosis of heterogametic sex is a suppression of recombination between non-homologous parts of sex chromosomes [153]. In contrast to meiosis, inactivation of an X chromosome in the specific interphase nuclei of *Thelyphonus* testes probably reflects a metabolic repression of this chromosome.

#### 4.1.7. Male Meiosis

Bivalents contained chiasmata during male meiosis in Pedipalpi and Ricinulei. Information regarding solifuge meiosis could not be obtained. The representatives of Schizomida and Ricinulei with predominantly acrocentric chromosomes had a low or moderate chiasma frequency, probably related to the chromosome morphology of these arachnids. In general, acrocentric chromosomes have fewer chiasmata than biarmed chromosomes [94]. Furthermore, a low level of chiasmata was observed in most amblypygids and thelyphonids that have chromosome numbers identical or close to the hypothetical ancestral diploid numbers of these orders [present study]. Surprisingly, the chiasma frequency of these Pedipalpi was low regardless of the predominance of monoarmed or biarmed chromosomes in their karyotypes. This unusual pattern indicates that some genetic regulatory mechanism exists in their genomes which maintains a low number of recombinations and chiasmata per chromosome. Among the Tetrapulmonata, a low number of chiasmata is characteristic, not only for schizomids and most amblypygids and thelyphonids with ancestral diploid numbers, but also for most spiders (e.g., [70,86]). This evolutionary distribution suggests a low chiasma frequency in ancestral tetrapulmonates. These findings, together with data available for other arachnid orders (see [154]), suggest that a low level of chiasmata is an ancestral feature of arachnids in general.

In amblypygids and thelyphonids, the frequency of male chiasmata was usually higher among species with low diploid numbers. Provided there is the same number of chiasmata per chromosome in species with high and low chromosome numbers, species with fewer chromosomes will have fewer chiasmata per karyotype. Therefore, the increased frequency of recombinations and chiasmata could be a compensatory mechanism to avoid the loss of genetic diversity associated with reduction in the number of chromosomes [155].

Male prophase I of Pedipalpi and Ricinulei included a period with an enormous decondensation of bivalents between pachytene and diplotene (the so-called diffuse stage). In the amblypygid *Heterophrynus*, diplotene was missing, and bivalents recondensed only during diakinesis. The diffuse stage arose multiple times during animal and plant evolution [130]. A considerable decondensation of chromatin during this period presumably indicates a high transcriptional activity of nuclei. This stage is common in females as a structural adaptation to the rapid production of nutritive reserves needed for the development of the ovum. However, it was also found in the males of some animal taxa [54,130]. Among tetrapulmonates, the male diffuse stage is characteristic not only of Pedipalpi, but also of haplogyne and some protoentelegyne, entelegyne, and mygalomorph spiders [54,55,130]. More data from spiders are necessary to determine whether or not the male diffuse stage is an ancestral trait for spiders. The diffuse stage was also described in the males of other arachnids, namely palpigrades as well as some pseudoscorpions, harvestmen, and acariform and parasitiform mites (reviewed by [71,82]).

### 4.2. Karyotype Evolution of Arachnids

#### 4.2.1. Solifugae and Putative Relatives

Based on morphology, solifuges are often considered the sister clade of pseudoscorpions, forming Haplocnemata (e.g., [156]). However, the karyotypes of these orders are very different, consistent with molecular phylogenetic hypotheses, most of which do not support a close relationship between pseudoscorpions and solifuges [46]. Solifuges are characterized by low diploid numbers and the absence of heteromorphic sex chromosomes. Their karyotypes usually exhibit a predominance of acrocentric chromosomes. In contrast, most pseudoscorpions have much higher diploid numbers than solifuges and a predominance of biarmed chromosomes. Moreover, pseudoscorpions have morphologically differentiated sex chromosomes. Most species have an X0 system with a large metacentric X chromosome exhibiting a specific behavior during male meiosis. This sex chromosome pattern is probably ancestral in pseudoscorpions [82].

Recently, however, acariform mites were suggested to be the sister group of Solifugae, forming a clade termed Poecilophysidea by Pepato et al. [28]. Support for this clade has been accumulating from ultrastructural and morphological features [157], and phylogenies based on molecular data [50,51] and combined molecular and morphological data [28] across Arachnida. Pepato et al. [28] and Ballesteros et al. [51] also recovered the Cephalosomata clade, comprising acariform mites, palpigrades, and solifuges. This hypothesis is supported by cytogenetic data in two respects. Firstly, the karyotypes of palpigrades and solifuges are very similar in terms of diploid numbers, chromosome morphology, NOR pattern, and sex chromosomes. The diploid numbers of palpigrades (2n = 14–18, [71]) fall within the range observed in solifuges (2n = 10–24). Palpigrade karyotypes probably also possess a predominance of acrocentric chromosomes and contain a single NOR locus [71], resembling the hypothetical ancestral karyotype of solifuges. Palpigrades and solifuges also have no heteromorphic sex chromosomes [71] (present study). Secondly, the karyotypes of acariform mites are similar in diploid numbers (2n = 4–26, [15]) to those of palpigrades and solifuges. Ancestral acariform mites probably possessed eighteen chromosomes, a number that is common in the karyotypes of both primary clades of acariform mites, namely the sarcoptiforms and trombidiforms. Moreover, most species with eighteen chromosomes belong to diploid clades of acariforms, which is the original ploidy level of these mites (see [15] for diploid numbers of mites). Among clades with occurrence of haplodiploidy, karyotypes composed of eighteen chromosomes were probably found in eupodids [158], an early diverging clade of trombidiforms [159]. Published data suggest an absence of heteromorphic sex chromosomes in ancestral acariform mites [15].

The karyotype evolution of acariform mites [160] and solifuges [present study] likely included multiple reductions of diploid numbers. Some palpigrade clades probably also have reduced diploid numbers [71]. The chromosome numbers of palpigrades and solifuges probably decreased mainly through tandem fusions [71] (present study). The evolution of some acariforms included an increase in diploid numbers by fissions [161].

Among solifuges, a derived karyotype was observed in galeodids and rhagodids [present study]. Recent molecular phylogenetic analyses of solifuges revealed a clade comprising galeodids and rhagodids [52], both of which exhibit a predominance of biarmed chromosome pairs and an enormous amount of CH, which are probably synapomorphic. Another possible synapomorphy of this clade is a considerable increase in the number of NOR loci, observed in the galeodid studied in the present investigation; the pattern of rhagodid NORs is unknown, however.

Acariform mites have two derived genomic features, namely extremely small genomes [162] and tiny holocentric chromosomes [15]. Holocentric chromosomes evolved multiple times from monocentric chromosomes during eukaryotic evolution [163,164]. Therefore, it is possible that they originated by various mechanisms. According to Diaz et al. [165] and Král et al. [53], the holocentric chromosomes of spiders arose through multiple chromosome fusions followed by an expansion of kinetic activity across most of the chromosome surface. However, this is improbable in acariform mites because the two orders to which they appear to be most closely related, Palpigradi and Solifugae, exhibit similar diploid numbers. The holocentric chromosomes of acariform mites could be a structural adaptation to ensure the correct segregation of tiny chromosomes arising by an extreme reduction of the genome. Due to dispersion of microtubule-binding sites along most of the surface of acariform chromosomes, the chromosome region organizing microtubules retained a size sufficient to ensure effective binding of microtubules. This origin of holocentric chromosomes could be widespread in eukaryotes, judging by the small size of the chromosomes and genomes in many taxa with holocentric chromosomes [164]. Alternatively, the holocentric chromosomes of acariform mites might have arisen from acrocentric chromosomes in which the end opposite to the centromere gained the ability to bind microtubules ([163], based on F. Marec pers. comm.). This hypothesis is based on the kinetic activity of both chromosomal ends during meiosis in some taxa that possess holocentric chromosomes, e.g., some acariform mites (e.g., [166]). The acrocentric karyotypes of Palpigradi and Solifugae, both potentially closely related to acariform mites, also support this hypothesis.

#### 4.2.2. Tetrapulmonata

Based on cytogenetic characters, two major tetrapulmonate lineages can be distinguished, i.e., Schizomida and a lineage comprising amblypygids, thelyphonids, and spiders. Surprisingly, the hypothetical ancestral karyotype of the first lineage, the schizomids, is almost identical to the hypothetical ancestral solifuge karyotype in terms of diploid numbers (2n = 22), chromosome morphology (composed exclusively of monoarmed chromosomes), NOR pattern (one NOR locus), and sex chromosomes (absence of heteromorphic sex chromosomes). Whereas the latter two traits are assumed to be ancestral in arachnids [59,71], the combination of the first two traits is common to the hypothetical ancestral karyotypes of both schizomids and solifuges. Therefore, this combination could be symplesiomorphic for cephalosomates (acariform mites, palpigrades, and solifuges) and schizomids, implying that schizomids are basal within Tetrapulmonata. According to this hypothesis, Cephalosomata would be sister to Arachnopulmonata and Schizomida sister to the remainder of Tetrapulmonata. A close relationship between cephalosomates and tetrapulmonates was suggested by the combined morphological and molecular phylogenetic analyses of Pepato et al. [28]. Considering the relatively basal position of Cephalosomata within Arachnida in some phylogenies [51], it is also possible that the common cytogenetic features of Cephalosomata and schizomids are symplesiomorphic not only for these groups but for all arachnids. Although most studies of arachnid phylogeny consider schizomids to be the sister group of thelyphonids, forming Uropygi ([8,27] and references therein), cytogenetic data do not support this hypothesis or the Pedipalpi clade. The only cytogenetic feature common to schizomids and thelyphonids is the male diffuse stage. The evolutionary distribution of this feature within tetrapulmonates suggests it is symplesiomorphic or arose independently several times. The hypothesised phylogenetic position of schizomids presented herein is also supported by the putative number of body segments, characters of prosoma, coxal gland, and sperm morphology (see [28,142]), which could be symplesiomorphic in cephalosomates and schizomids. The phylogenetic position of schizomids relative to other tetrapulmonates requires further investigation.

The second tetrapulmonate lineage implied by the cytogenetic data comprises amblypygids, thelyphonids, and spiders (hereafter referred to as “higher” tetrapulmonates). Cytogenetic data suggest that the ancestor of this lineage had a high diploid number (70–80 chromosomes), a predominance of monoarmed chromosomes, and a low to moderate amount of CH located mostly in the centromere and telomere regions. The ancestral diploid number of higher tetrapulmonates was probably close to the upper limit of the abovementioned range of chromosome numbers.

Comparison of the hypothetical ancestral karyotypes of schizomids and higher tetrapulmonates suggests that a considerable increase in the number of chromosomes during tetrapulmonate evolution predated the divergence of amblypygids, thelyphonids, and spiders. The most probable explanation for this increase is through one or two rounds of polyploidization. Two polyploidization events would transform the hypothetical ancestral schizomid karyotype into a set in which the number of chromosomes is close to the ancestral karyotype of higher tetrapulmonates. Genome polyploidization in ancient tetrapulmonates is supported by phylogenomic data for spiders, thelyphonids, and amblypygids. Many genes of these arachnids, including *HOX* genes, are duplicated, reflecting an ancient duplication of their genomes [43,167,168]. Interestingly, *HOX* genes are not duplicated in cephalosomate representatives, the acariform mite *Archegozetes* [169] and the solifuge, *Titanopuga* [170].

It has been suggested previously that tetrapulmonates share a genome duplication with pseudoscorpions and scorpions [46]. This hypothesis requires further testing, however. Based on the data presented herein, two separate events of genome duplication are hypothesized to have occurred in arachnopulmonates; one in the ancestor of pseudoscorpions and scorpions, and another in the ancestor of higher tetrapulmonates. Comparative genome mapping of spiders and other tetrapulmonates could be a reliable approach to test the hypotheses of polyploidization in tetrapulmonates. This would require genome analysis of schizomids.

The available data suggest that polyploid events were relatively common during arachnid evolution. Two independent events were revealed in the *Leiobunum* harvestmen [81] and two others in metastriate ticks [171,172]. Polyploidy is also suspected in an argasid tick [173]. Furthermore, the genomes of some arachnid clades underwent an ancient duplication (i.e., paleopolyploidy). Aside from the event discussed above [46], arachnopulmonate evolution likely included additional genome duplications, namely in caponiid [53] and mygalomorph spiders [55]. Based on genome sizes, it was hypothesized that one or even two paleopolyploid events occurred in ticks [174,175]. However, this hypothesis is unsupported by chromosome (see Section 4.2.3 below) and phylogenomic data [43]. Ancient polyploidy is also suspected in laniatorid harvestmen [117] and atemnid pseudoscorpions [176]. One reason for a relatively high frequency of polyploid events during arachnid evolution could be the low number of arachnid clades with highly differentiated sex chromosomes. Their genes often interfere with genome duplication due to a disruption of dosage compensation [177].

Polyploid events also interfere with meiotic processes [178]. Maintenance of standard meiosis in polyploids can be promoted through specific mechanisms, which may accelerate the meiotic diploidization of polyploids by promoting homologous chromosome pairing. Attachment of homologous chromosomes can be promoted by a low recombination frequency [179]. As argued below, somatic pairing of homologous chromosomes could fulfill the same function. Both mechanisms were demonstrated to be present in some tetrapulmonates [present study], which implies that they could have been present in the diploid ancestor of higher tetrapulmonates as pre-adaptations to genome duplication. The formation of multivalents is substantially reduced in polyploids with low recombination levels [180]. Therefore, this advantageous recombination pattern is often fixed through selection and genetically determined in established polyploids [181]. Within this context, it is remarkable that data presented herein indicate genetic control of low recombination level in those amblypygids and thelyphonids with a karyotype closest to the hypothetical ancestral karyotype of tetrapulmonate polyploids. Provided that the ancient higher tetrapulmonates were allopolyploid, the separation of their homologous and homeologous chromosomes could have been promoted through somatic pairing, which would have initiated the alignment of homologs already in the gonial cells.

#### 4.2.3. Ricinulei

The position of Ricinulei is unresolved in the hypothesis of karyotype evolution presented. Ricinulei have been variously placed as sister to tetrapulmonates [28], solifuges [6], mites [4,156], or parasitiform mites [31,33,51]. However, the ancestral diploid number of ricinuleids (40 chromosomes) is much higher than those of the ancestral tetrapulmonates and ancestral cephalosomates, the latter of which include solifuges. This could imply a derived position of extant Ricinulei. The data available are insufficient to reconstruct the ancestral karyotype of parasitiform mites, but these arachnids also have lower diploid numbers than ricinuleids (see [15] for karyotypes of parasitiforms). Based on the available data, the original male karyotype of ticks, one of three primary clades of parasitiforms, probably comprised 26 or 28 chromosomes including homomorphic or slightly heteromorphic sex chromosomes X and Y. Low differentiation of sex chromosomes is retained in most argasid ticks [173]. This hypothesis is supported by the karyotype of a representative of Antennophorina (the most early diverging clade of Mesostigmata, another primary clade of parasitiforms), which is composed of 26 chromosomes and does not contain heteromorphic sex chromosomes [182]. Diploid numbers decreased during the karyotype evolution of ticks.

The ancestral karyotype of ricinuleids differs considerably from the hypothetical ancestral karyotypes of harvestmen, pseudoscorpions, and scorpions, consistent with molecular phylogenetic hypotheses, which do not support a relationship between ricinuleids and any of these orders.

The ancestral diploid numbers of harvestmen, pseudoscorpions, and scorpions were higher than those of cephalosomates and tetrapulmonates. The ancestral harvestmen karyotype probably comprised 30 chromosomes with predominantly biarmed morphology and did not contain heteromorphic sex chromosomes [154]. Notably, the hypothetical ancestral karyotypes of harvestmen and ticks are similar, which could reflect a relationship between harvestmen and parasitiforms. A close relationship between these two orders was also suggested by Dunlop [37].

The ancestral diploid number of scorpions was suggested to be close to the diploid numbers of buthids [183], which range from 5 to 56 [17]. Although these scorpions have some plesiomorphic morphological features [184], they have holocentric chromosomes, a derived trait [71,163,164], the origin of which could be accompanied by a considerable decrease in diploid numbers [53,165]. Consistent with this hypothesis, the comparison of diploid numbers and molecular data suggests a low ancestral diploid number in buthids (6–32 chromosomes) [127]. It is therefore more plausible to reconstruct the hypothetical ancestral scorpion karyotype based on clades with standard chromosomes. The karyotypes of chaerilids, which along with pseudochactids form the sister group of buthids [185], comprise a high number (76–186) of standard chromosomes [17]. Iurida, the sister group of the clade comprising chaerilids, pseudochactids and buthids, includes all other scorpions. The most early diverging clade of this lineage is family Iuridae [185] in which high numbers of monocentric chromosomes are also found [17]. This phylogenetic pattern suggests that the ancestral diploid number of scorpions was much higher than those of extant buthids. High chromosome numbers of ancestral scorpions could reflect polyploidy in these arachnids. The ancestral karyotype of pseudoscorpions is also supposed to comprise a high diploid number [186], which could also reflect polyploidy in these arachnids.

## 5. Conclusions

The present study aimed to reconstruct fundamental traits in the karyotype evolution of tetrapulmonate arachnids, a major arthropod clade comprising the orders Araneae (spiders), Amblypygi, Thelyphonida, and Schizomida. The taxa examined represented most of the major clades of the three non-spider tetrapulmonate orders, plus two additional orders with unresolved placement, Ricinulei and Solifugae. Except for Amblypygi, the cytogenetics of these orders was largely unknown prior to the present study.

The most important cytogenetic events hypothesized in arachnid evolution are summarized in Table 7. This study, combined with previous data, suggests that ancestral arachnids had low to average diploid numbers, monocentric chromosomes, a single NOR locus, low levels of heterochromatin and recombinations, and none or homomorphic sex chromosomes. The arthropod motif of telomeric repeats was retained in arachnids except spiders. Furthermore, available data suggest a relatively common occurrence of polyploidy during arachnid evolution.

Although amblypygids and thelyphonids each contain far fewer species than spiders, both orders exhibit considerable karyotype differentiation. The hypothetical ancestral karyotype of Amblypygi probably comprised 74–78 predominantly monoarmed chromosomes. The present study, combined with previously published data, suggests that the ancestral karyotype of thelyphonids and spiders had a diploid number close to the upper limit of the ancestral diploid number of amblypygids and a similar proportion of biarmed and monoarmed chromosomes to that of ancestral amblypygids. Although thelyphonids exhibiting the hypothetical ancestral karyotype belong to different subfamilies, they are restricted to the Indian subcontinent, which may reflect a Gondwanan origin of thelyphonids. The karyotypes of amblypygids and thelyphonids diversified mainly via centric fusions and pericentric inversions.

Ricinuleids, schizomids, and solifuges exhibit less karyotype diversity than amblypygids and thelyphonids, which facilitates reconstruction of their karyotype evolution. The ancestor of extant ricinuleids probably exhibited 40 predominantly biarmed chromosomes. The karyotype differentiation of New World ricinuleids included pericentric inversions and chromosome fissions. Remarkably, the hypothetical ancestral karyotypes of schizomids and solifuges are similar in diploid number (22 chromosomes in schizomids, 24 chromosomes in solifuges) and chromosome morphology (all or almost all chromosomes acrocentric). Available data suggest the occurrence of centric fusions and pericentric inversions during schizomid karyotype evolution, as well as tandem fusions during solifuge karyotype evolution. Homologous chromosomes of solifuges are associated throughout the cell cycle. Galeodids and rhagodids differ from other solifuges in two possible synapomorphies, i.e., predominantly biarmed chromosomes and an enormous amount of constitutive heterochromatin. The considerable increase in heterochromatin could promote somatic chromosome pairing and formation of a specific environment for NOR activity.

The hypothetical ancestral karyotype of arachnids probably contained a single NOR. The number of NOR loci increased in some tetrapulmonate clades, specifically galeodid solifuges, some amblypygids (*Charon* and *Phrynoidea*), and thelyphonids. The subsequent evolution of Phrynoidea and thelyphonids included frequent reductions in the number of NOR loci. Centric fusions in the amblypygids *Euphrynichus* and *Paraphrynus* led to a relocation of NOR loci into the pericentric region. The interstitial NOR locus observed in two schizomids probably arose by an inversion.

The present study suggests that the ancestor of amblypygids, thelyphonids, and spiders had homomorphic sex chromosomes X and Y. The differentiation of MSY in amblypygids could be promoted by a recombination suppression from adjacent structures (telomeric heterochromatin, NOR). The sex chromosomes X and Y differentiated morphologically in only a few amblypygids (*Charon*, *Paraphrynus*) and thelyphonids (*Thelyphonus*). In *Charon*, this process involved the loss of Y chromosome-linked NOR, which could promote structural differentiation of the presumed Y chromosome. Except for *Thelyphonus*, differentiation of sex chromosomes was not accompanied by their inactivation in the male germline.

The prophase of the first meiotic division includes a diffuse stage in males of tetrapulmonate arachnids (except for some spider taxa) as well as some other arachnid clades (e.g., Palpigradi and Ricinulei). The male diffuse stage is either symplesiomorphic for Tetrapulmonata or originated independently several times during the evolution of this clade.

It is hypothesized that ancestral arachnids exhibited 30–40 chromosomes. The hypothetical ancestral karyotypes of harvestmen (30) and ricinuleids (40) fall within this range. The present study supports the Cephalosomata clade of arachnids (including acariforms, palpigrades, and solifuges) and a sister relationship between cephalosomates and tetrapulmonates. It is hypothesized that the diploid number decreased to 22–24 in the common ancestor of cephalosomates and tetrapulmonates, the karyotype of which was composed exclusively of monoarmed chromosomes. Alternatively, this karyotype could be ancestral also for arachnids. The holocentric chromosomes of acariforms could have evolved by the spreading of kinetic activity to most of the surface of their tiny chromosomes, to ensure correct chromosome segregation. Alternatively, holocentric chromosomes arose from acrocentric chromosomes of which the end opposite to the centromere gained the ability to bind microtubules.

Unlike current phylogenetic hypotheses, the results presented herein suggest a sister relationship between Schizomida and a clade comprising the remaining orders of Tetrapulmonata (Araneae, Amblypygi, and Thelyphonida). These results further support an ancient polyploidization in the clade comprising Araneae, Amblypygi and Thelyphonida. Polyploidy of the ancient tetrapulmonates could be maintained by specific mechanisms, observed in some arachnids in the present study, namely by low recombination frequency during meiosis and by somatic pairing of homologous chromosomes.

To fully reconstruct the karyotype evolution of arachnids, more data on their cytogenetics are needed. The cytogenetics of key clades, especially in acariforms, harvestmen, parasitiforms, pseudoscorpions, scorpions, and many spiders, remains unknown or poorly studied. Future investigations should identify additional cytogenetic characters suitable for phylogenetic analysis, include more DNA markers, as well as identify conservative syntenic regions and their rearrangements during arachnid evolution. This approach, along with comparative phylogenomic analysis of particular arachnid orders, would allow more accurate mapping of polyploid events in arachnids. Ancient genome duplications observed in the clade comprising pseudoscorpions and scorpions may be shared with tetrapulmonates or may represent an independent evolutionary event.

## Figures and Tables

**Table 1:** Karyotype data on Pedipalpi, Ricinulei, and Solifugae. Some chromosome pairs exhibited a transitional morphology between two types, denoted by morphology a/morphology b. *^1^ In some plates, two additional small pairs were also biarmed. *^2^ One nucleolus organizer region (NOR) locus was heterozygous for the presence of an NOR in some specimens (i.e., one chromosome of the chromosome pair included NOR only). *^3^ NOR locus heterozygous for NOR size. Abbreviations: ♂, male; ♀, female; ♂♀, both in males and females; +, TTAGG sequence detected; ap, acrocentric pair; bip, biarmed pair; bip and monop, karyotype comprises both biarmed and monoarmed chromosome pairs; j, juvenile of unknown sex; monop, monoarmed pair; mp, metacentric pair; NORarm, NOR occupies most of chromosome arm; NORi, NOR at interstitial region of chromosome arm; NORperic, pericentric NOR; NORpt, NOR at end of short arm; NORqi, NOR at interstitial region of long arm; NORqt, NOR at the end of long arm; NORsubt, subterminal NOR; NORt, terminal NOR; p, chromosome pair; SC, sex chromosomes; smp, submetacentric pair; stp, subtelocentric pair; TS, telomeric sequence; unknown morphology, unknown morphology of chromosome pair; Xa, acrocentric X chromosome; Xm, metacentric X chromosome; Xsm, submetacentric X chromosome; Ya, acrocentric Y chromosome; Ym, metacentric Y chromosome; Ysm, submetacentric Y chromosome.

Species	Source of Data	2n	Morphology of Autosome Pairs	SC	NOR Number	Number and Morphology of NOR-Bearing Chromosomes/Pairs (NOR Location)	TS (TTAGG)
**Amblypygi**							
Charontidae							
*Charinus cavernicolus* Weygoldt, 2006	[62]	76♂, j	5 mp, 9 smp, 18 stp, 6 ap				
*Charinus dominicanus* Armas and González, 2002	[62]	42♂	16 mp, 5 smp				
*Charinus neocaledonicus* Simon, 1895	[62]	74♂	9 mp, 2 smp, 2 stp, 24 ap		1	1 p unknown morphology (NORt)	
*Charinus pescotti* Dunn, 1949	[62]	74♂	9 mp, 5 smp, 23 ap		1	1 p unknown morphology (NORt)	
*Charon* cf. *grayi* (Gervais, 1842)	this study	70♂♀	4 mp, 3 smp, 27 monop	XY (Xa, Ya)	2	1 ap (NORpt), 1 Xa (NORpt)	
*Sarax* aff. *batuensis* Roewer, 1962	[62]	56♂	5 mp, 8 smp, 6 stp, 9 ap				
*Sarax huberi* Seiter et al., 2015	[62]	50♂	15 mp, 2 stp, 8 ap				
*Sarax ioanniticus* Kritscher, 1959	[62]	72♀	monop predominant				
*Sarax seychellarum* Kraepelin, 1898	[62]	22♂♀	9 mp, 2 smp		1	1 mp (NORi)	+
*Sarax* sp. (*brachydactylus* group)	[62]	74♂	9 mp, 3 smp, 2 stp, 23 ap				
Phrynichidae							
*Damon medius* (Herbst, 1797)	this study	♂	bip and monop		2	2 smp (each pair with NORpt)	
	[67]	70♂					+
*Euphrynichus amanica* (Werner, 1916)	this study	78♂	15 mp, 4 smp, 20 monop		3	1 mp (NORt), 1 stp (NORpt), 1 ap (NORpt)	
*Euphrynichus bacillifer* (Gerstaecker,1873)	this study	56♂	22 mp, 2 smp, 1 stp, 3 ap		1	1 mp (NORperic)	
*Phrynichus ceylonicus* (C.L. Koch, 1843)	this study	52♂♀	17 mp, 3 mp/smp, 5 smp, 1 stp		3	3 mp (each pair with NORt)	
Phrynidae							
*Acanthophrynus coronatus* (Butler, 1873)	this study	86♂	27 bip, 6 stp, 10 ap		1	1 stp (NORpt)	+
*Heterophrynus* cf. *elaphus* Pocock, 1903	this study	76♂	17 bip, 21 monop		1	1 ap (NORqt)	
*Heterophrynus longicornis* Butler, 1873	[61]	66♂♀	24 mp, 3 smp, 6 stp		2	2 mp (each pair with NORt)	
*Paraphrynus aztecus* Pocock, 1894	[60]	36♂	14 mp, 4 smp				
*Paraphrynus carolynae* Armas, 2012	[60]	30♂	12 mp, 3 smp				
*Paraphrynus cubensis* Quintero, 1983	[60]	34♂	16 mp, 1 sm				
*Paraphrynus mexicanus* (Bilimek, 1867)	this study	24♂♀	11 bip	XY (Xm, Ysm)	1	1 mp (NORperic)	
	[60]	24♂	9 mp, 3 smp				
*Paraphrynus pseudomexicanus* Seiter et al., 2020	[60]	32♂	8 mp, 1 smp, 7 ap				
*Paraphrynus robustus* Franganillo, 1931	[60]	64♂	16 mp, 6 smp, 2 st, 8 ap				
*Phrynus marginemaculatus * C.L. Koch, 1840	[60]	68♂	14 mp, 5 smp, 5 stp, 10 ap				
**Thelyphonida**							
Thelyphonidae (Hypoctoninae)							
*Hypoctonus* cf. *gastrostictus* Kraepelin, 1897	this study	66♂	22 bip, 2 stp, 9 ap		2	2 mp (each pair with NORt)	
*Labochirus proboscideus* (Butler, 1872)	this study	78♂♀	6 mp, 2 mp/smp, 3 smp, 28 monop *^1^				
*Yekuana venezolensis* (Haupt, 2009)	this study	38♂	4 mp, 1 mp/smp, 4 smp, 1 stp, 9 ap		1	1 smp (NORpt)	
Thelyphonidae (Mastigoproctinae)							
*Mastigoproctus giganteus* (Lucas, 1835)	this study	28♂♀	7 mp, 1 smp, 1 smp/stp, 1 stp, 4 ap		2	2 mp (each pair with NORt)	
*Uroproctus assamensis* (Stoliczka, 1869)	this study	72♂♀	4 mp, 3 smp,1 smp/stp, 28 monop		3	3 p unknown morphology (NORt)	
Thelyphonidae (Thelyphoninae)							
*Ginosigma* sp.	this study	40♂	4 mp, 4 smp, 1 smp/stp, 4 stp, 7 ap		3	1 mp (NORpt), 1 smp/stp (NORpt), 1 stp (NORpt)	+
*Thelyphonus* cf. *linganus* C.L. Koch, 1843	this study	66♂♀	19 bip, 3 stp, 10 ap	XY (Xsm, Ym)			
*Thelyphonus sepiaris* Butler, 1873	[64]	44♂	21 ap	XY (Xa, Ya)			
	[65]	42♂♀	1 mp, 8 stp, 12 ap				
Thelyphonidae (Typopeltinae)							
*Typopeltis crucifer* Pocock, 1894	this study	40♀					
*Typopeltis guangxiensis* Haupt and Song, 1996	this study	36♂♀	6 mp, 4 smp, 1 stp, 7 ap		5	1 mp (NORt), 1 mp (NORpt + NORqt)*^2^, 2 ap (each pair with NORqt)	
**Schizomida**							
Hubbardiidae							
*Clavizomus* sp.	this study	22♂	1 mp, 3 stp, 7 ap				
*Notozomus* sp.	this study	22♂	2 stp, 9 ap				
*Orientzomus* sp. (Luzon)	this study	16♂♀	5 bip, 3 ap				
*Orientzomus* sp. (Mindanao)	this study	16♀			1	1p unknown morphology (NORt)	
*Stenochrus* sp.	this study	22♂♀	8 stp, 3 ap				+
*Olmecazomus brujo* Monjaraz-Ruedas and Francke, 2017	this study	22♂♀	11 ap		1	1 ap (NORqi)	
*Rowlandius ubajara * Santos et al., 2013	[66]	22♀	1 mp, 10 monop				
*Rowlandius* sp.	[66]	20♀	1 mp, 9 monop				
Hubbardiidae sp. (Seychelles)	this study	22♀,j	11 ap		1	1 ap (NORt)	
Hubbardiidae sp. (Cameroon)	this study	22♀, j	11 ap		1	1 ap (NORqi)	
Protoschizomidae							
*Agastoschizomus lucifer* Rowland, 1971	this study	22♀	11 ap		1	1 ap (NORt)	
**Ricinulei**							
*Cryptocellus narino* Platnick and Paz, 1979	this study	46♂♀	1 mp, 1 mp/smp, 2 smp, 1 stp, 18 ap		1	1 p unknown morphology (NORsubt)	
*Pseudocellus gertschi* (Márquez and Conconi, 1974)	this study	40♂♀	2 mp, 4 smp, 1 smp/stp, 13 monop		1	1 ap (NORqt)*^3^	+
*Ricinoides olounoua* Legg, 1978	this study	40♂	16 bip, 4 stp		1	1 p unknown morphology (NORsubt)	
**Solifugae**							
Ammotrechidae							
*Ammotrechula mulaiki* Muma, 1951	this study	24♂♀	2 stp, 10 ap				
Daesiidae							
*Eberlanzia flava* Roewer, 1941	this study	22♂	2 mp, 2 stp, 7 ap		1	1 stp (NORpt)	
*Gluvia dorsalis* (Latreille, 1817)	this study	10♂♀	5 ap				
*Gnosippus* sp.	this study	16♂	karyotype predominated by ap				
Eremobatidae							
*Eremobates pallipes* (Say, 1823)	this study	22♂	1 mp, 2 stp, 8 ap		1	1 mp (NORarm)	
*Eremobates similis* Muma, 1951	this study	22♂	1 mp, 10 ap		1	1 p unknown morphology (NORt)	
Galeodidae							
*Paragaleodes pallidus* (Birula, 1890)	this study	12♂♀	6 mp		8	2 mp (each pair with NORsubt), 3 mp (each pair with NORpsubt +	
						NORqsubt)	
Rhagodidae							
*Rhagodes* sp.	this study	18j	7 mp, 2 stp				
	[67]	18j					+
Solpugidae							
*Solpugista* sp.	this study	20♀	10 ap		1	1 ap (NORt) *^3^	

**Table 2:** Distribution of cytogenetic characters scored for 24 representatives of order Amblypygi and hypothetical (all-zero) outgroup. Characters scored 0–9 and then A–G, with missing (unknown) entries indicated by ? Character descriptions listed below.

Outgroup	00000
*Acanthophrynus coronatus*	12000
*Charon* cf. *grayi*	30101
*Charinus cavernicolus*	00???
*Charinus dominicanus*	A2???
*Charinus neocaledonicus*	00000
*Charinus pescotti*	00000
*Damon medius*	3?100
*Euphrynichus amanica*	01200
*Euphrynichus bacillifer*	72020
*Heterophrynus* cf. *elaphus*	00000
*Heterophrynus longicornis*	52100
*Paraphrynus aztecus*	B2???
*Paraphrynus carolynae*	E2???
*Paraphrynus cubensis*	C2???
*Paraphrynus mexicanus*	F2020
*Paraphrynus pseudomexicanus*	D1???
*Paraphrynus robustus*	62???
*Phrynichus ceylonicus*	82200
*Phrynus marginemaculatus*	42???
*Sarax* aff. *batuensis*	71???
*Sarax huberi*	92???
*Sarax ioanniticus*	20???
*Sarax seychellarum*	G2010
*Sarax* sp. (*brachydactylus* group)	00???

**Number and morphology of chromosomes. 1.** Diploid number: (0) 74–78; (1) 86; (2) 72; (3) 70; (4) 68; (5) 66; (6) 64; (7) 56; (8) 52; (9) 50; (A) 42; (B) 36; (C) 34; (D) 32; (E) 30; (F) 24; (G) 22. **2.** Morphology of chromosome pairs: (0) monoarmed pairs predominant; (1) approximately same number of biarmed and monoarmed pairs; (2) biarmed pairs predominant. **Nucleolus organizer regions (NORs). 3.** Total number of NOR loci: (0) 1; (1) 2; (2) 3. **4.** Location of NOR loci on chromosome pairs: (0) terminal; (1) interstitial; (2) pericentric. 5. Sex-chromosome linked NORs: (0) NORs located exclusively on autosome pairs; (1) karyotype includes presumably X-chromosome linked NOR.

**Table 3:** Distribution of cytogenetic characters scored for ten representatives of order Thelyphonida and hypothetical (all-zero) outgroup. Character states scored 0–6, with missing (unknown) entries indicated by ? Character descriptions listed below.

Outgroup	000000
*Ginosigma* sp.	300000
*Hypoctonus* cf. *gastrostictus*	120000
*Labochirus proboscideus*	000??0
*Mastigoproctus giganteus*	620000
*Thelyphonus* cf. *linganus*	121??0
*Thelyphonus sepiaris*	201??0
*Typopeltis crucifer*	3?????
*Typopeltis guangxiensis*	510200
*Uroproctus assamensis*	000000
*Yekuana venezolensis*	410100

**Number and morphology of chromosomes. 1.** Diploid number: (0) 72–78; (1) 66; (2) 42–44; (3) 40; (4) 38; (5) 36; (6) 28. **2.** Morphology of chromosome pairs: (0) monoarmed pairs predominant; (1) approximately same number of biarmed and monoarmed pairs; (2) biarmed pairs predominant. **3.** Morphological differentiation of putative sex chromosomes X and Y: (0) sex chromosomes do not exist or are homomorphic; (1) sex chromosomes are heteromorphic. **Nucleolus organizer regions (NORs). 4.** Total number of NOR loci: (0) 2 or 3; (1) 1; (2) 5. **5.** Location of NOR loci on chromosome pairs: (0) terminal. **Constitutive heterochromatin. 6.** Pattern of heterochromatin: (0) heterochromatin not detected by microscopy or small blocks of heterochromatin at centromeres and telomeres, respectively.

**Table 4:** Distribution of cytogenetic characters scored for eleven representatives of order Schizomida and hypothetical (all-zero) outgroup. Character states scored 0–3, with missing (unknown) entries indicated by ? Character descriptions listed below.

Outgroup	0000
*Agastoschizomus lucifer*	0000
*Clavizomus* sp.	00??
Hubbardiidae (Cameroon)	0001
Hubbardiidae (Seychelles)	0000
*Notozomus* sp.	00??
*Olmecazomus brujo*	0001
*Orientzomus* sp. (Luzon)	21??
*Orientzomus* sp. (Mindanao)	2?00
*Rowlandius ubajara*	00??
*Rowlandius* sp.	10??
*Stenochrus* sp.	00??

**Number and morphology of chromosomes. 1.** Diploid number: (0) 22; (1) 20; (2) 16. **2.** Morphology of chromosome pairs: (0) all or almost all pairs monoarmed; (1) approximately same number of biarmed and monoarmed pairs. **Nucleolus organizer regions (NORs). 3.** Total number of NOR loci: (0) 1. **4.** Location of NOR locus on chromosome pair: (0) terminal; (1) interstitial.

**Table 5:** Distribution of cytogenetic characters scored for three representatives of order Ricinulei and hypothetical (all-zero) outgroup. Character states scored 0 and 1. Character descriptions listed below.

Outgroup	0000
*Cryptocellus narino*	1100
*Pseudocellus gertschi*	0110
*Ricinoides olounoua*	0010

**Number and morphology of chromosomes. 1.** Diploid number: (0) 40; (1) 46. **2.** Morphology of chromosome pairs: (0) most pairs biarmed; (1) most pairs monoarmed. **3.** Relative size of first chromosome pair: (0) chromosome pairs decrease gradually in length; (1) first pair considerably larger. **Nucleolus organizer regions (NORs). 4.** Total number of NOR loci: (0) 1.

**Table 6:** Distribution of cytogenetic characters scored for nine representatives of order Solifugae and hypothetical (all-zero) outgroup. Character states scored 0–6, with missing (unknown) entries indicated by ? Character descriptions are listed below.

Outgroup	00000
*Ammotrechula mulaiki*	00???
*Eberlanzia flava*	10000
*Eremobates pallipes*	10000
*Eremobates similis*	10000
*Gluvia dorsalis*	60??0
*Gnosippus* sp.	40???
*Paragaleodes pallidus*	51111
*Rhagodes* sp.	31??1
*Solpugista* sp.	20000

**Number and morphology of chromosomes. 1.** Diploid number: (0) 24; (1) 22; (2) 20; (3) 18; (4) 16; (5) 12; (6) 10. **2.** Morphology of chromosome pairs: (0) all or almost all pairs acrocentric; (1) biarmed pairs predominant. **Nucleolus organizer regions (NORs). 3.** Total number of NOR loci: (0) 1; (1) most chromosome pairs include one or two NOR loci. **4.** Location of NOR loci on chromosome pairs: (0) terminal; (1) subterminal. **Constitutive heterochromatin. 5.** Pattern of heterochromatin: (0) heterochromatin not detected by microscopy or small centromeric blocks of heterochromatin; (1) enormous blocks of heterochromatin.

**Table 7:** Summary of hypotheses concerning ancestral cytogenetic features of arachnid taxa. Alternative hypotheses in brackets {}.

Taxon	Ancestral Pattern	Hypothesis Source
	**Male diploid number or genome event**	
Arachnida	low to moderate 2n (30–40) {low 2n (22–24)}	this study
Arachnopulmonata	duplication of genome	[46]
Cephalosomata + Arachnopulmonata	22–24	this study
Higher Tetrapulmonata (Amblypygi + Thelyphonida + Araneae)	duplication of genome	this study
Panscorpiones (Pseudoscoriones + Scorpiones)	duplication of genome	this study
Acariformes	18	this study
Amblypygi	74–78	this study
Opiliones	30	[154]
Pseudoscorpiones	a high diploid number	[186]
Ricinulei	40	this study
Schizomida	22	this study
Scorpiones	a high diploid number	this study
Solifugae	24	this study
Amblypygi (Charontidae)	74–76	this study
Amblypygi (Phrynoidea)	76–78	this study
Araneae (Araneomorphae, Araneoidea)	24	[187]
Araneae (Araneomorphae, Caponiidae)	duplication of genome	[53]
Araneae (Araneomorphae, Entelegynae)	42	[130]
Araneae (Mygalomorphae, Avicularioidea)	70–90, duplication of genome	[55]
Araneae (Opisthothelae)	40–50	[130]
Opiliones (Laniatores)	duplication of genome {increase in diploid number by chromosome fissions}	[117]
Parasitiformes (Ixodida)	26–28	this study
Pseudoscorpiones (Atemnidae)	duplication of genome {increase in diploid number by chromosome fissions}	[176]
Scorpiones (Buthidae)	6–32	[127]
Thelyphonida	72–78	this study
	**Chromosome morphology**	
Arachnida	monocentric chromosomes	[71]
Clade Cephalosomata + Arachnopulmonata	monoarmed chromosomes, predominantly acrocentric elements	this study
Higher Tetrapulmonata (Amblypygi + Thelyphonida + Araneae)	predominantly monoarmed chromosomes (1/3 of chromosomes biarmed)	this study
Acariformes	holocentric chromosomes	[15]
Opiliones	predominantly biarmed chromosomes	[154]
Ricinulei	predominantly biarmed chromosomes	this study
Schizomida	acrocentric chromosomes	this study
Solifugae	almost all or all chromosomes acrocentric	this study
Amblypygi (Phrynoidea)	karyotype includes 17–19 biarmed pairs	this study
Araneae (Araneomorphae, Dysderoidea)	holocentric chromosomes	[130]
Araneae (Araneomorphae, Entelegynae)	acrocentric chromosomes	[86]
Araneae (Opisthothelae)	predominantly biarmed chromosomes	[55]
Scorpiones (Buthidae)	holocentric chromosomes	[188]
Solifugae (Galeodidae + Rhagodidae)	predominantly biarmed chromosomes	this study
	**Chromosome behaviour throughout cell cycle**	
Solifugae	pairing of homologous chromosomes throughout cell cycle	this study
	**Constitutive heterochromatin**	
Arachnida	low to moderate content of constitutive heterochromatin	this study
Arachnida	constitutive heterochromatin located in centromeric and telomeric regions	this study
Arachnida	centromeric heterochromatin predominantly AT-rich	this study
Araneae	constitutive heterochromatin located at centromeric regions only	[189]
Solifugae (Galeodidae + Rhagodidae)	considerable expansion of constitutive heterochromatin	this study
	**NORs**	
Arachnida	1 NOR	[59]
Arachnida	terminal position of NORs	[61]
Amblypygi	1 NOR	this study
Thelyphonida	2 or 3 NORs	this study
Amblypygi (*Phrynichus* + *Euphrynichus*)	3 NORs	this study
Amblypygi (Phrynidae)	two subtelocentric NOR-bearing chromosome pairs	this study
Amblypygi (Phrynoidea)	2 NORs	this study
Araneae (Araneomorphae, Entelegynae)	2 autosome NORs	[70]
Araneae (Opisthothelae)	1 or 2 autosome NORs	[55]
Schizomida (Mexican + West African clade of Hubbardiidae)	interstitial NOR	this study
Solifugae (Galeodidae)	a high number of NORs	this study
	**Telomeric repeats**	
Arachnida	insect motif	[67]
Araneae	absence of insect motif	[67]
	**Sex chromosomes (heterogametic sex)**	
Arachnida	homomorphic sex chromosomes or absence of sex chromosomes	[71]
Higher Tetrapulmonata (Amblypygi + Thelyphonida + Araneae)	homomorphic sex chromosomes X and Y	this study
Araneae	homomorphic sex chromosomes X and Y + X_1_ and X_2_	[56]
Pseudoscorpiones	X0 system	[190]
Pseudoscorpiones	metacentric morphology of X chromosome	[190]
Scorpiones	homomorphic sex chromosomes X and Y	[125]
Araneae (Araneomorphae)	homomorphic sex chromosomes X and Y + chromosomes X_1_, X_2_, and Y	[73,191]
Araneae (Mygalomorphae, Avicularioidea)	two homomorphic XY pairs + X_1_X_2_X_3_X_4_ (result of genome duplication)	[55]
Parasitiformes (Ixodida)	sex chromosomes X and Y	this study
	**Male meiosis (heterogametic sex)**	
Arachnida	low frequency of chiasmata	this study
Tetrapulmonata	diffuse stage {diffuse stage arose several times during evolution of Tetrapulmonata}	this study
Araneae	specific end-to-end pairing of X chromosome univalents	[57]
Palpigradi	diffuse stage	[71]
Ricinulei	diffuse stage	this study
Scorpiones	achiasmatic meiosis	[192]
Araneae (Araneomorphae, Haplogynae)	diffuse stage	[130]
Pseudoscorpiones (Chthoniidae)	achiasmatic meiosis	[186]

## Data Availability

The original contributions presented in this study are included in the article/Supplementary Materials. Further inquiries can be directed to the corresponding author.

## Supplementary Materials

The following supporting information can be downloaded at: https://www.mdpi.com/article/10.3390/genes16020207/s1, **Figure S1**. Amblypygi: Phrynichidae: *Euphrynichus amanica*, male karyotype. Based on two sister metaphases II (Giemsa staining). Scale bar = 10 μm. **Figure S2.** Amblypygi: Phrynichidae: *Euphrynichus bacillifer* (A) and *Phrynichus ceylonicus* (B), male karyotypes. Based on two sister metaphases II (Giemsa staining). Scale bars = 10 μm. **Figure S3.** Amblypygi: Phrynichidae: *Damon medius*, male, pattern of NORs (red). Metaphase II (FISH, DAPI staining). Note two submetacentric chromosomes (belonging to different chromosome pairs) bearing terminal NOR (arrowheads). Scale bar = 10 μm. **Figure S4.** Thelyphonida: Thelyphonidae: Hypoctoninae: *Hypoctonus* cf. *gastrostictus* (A) and *Labochirus proboscideus* (B), male karyotypes. Based on two sister metaphases II (Giemsa staining). Scale bars = 10 μm. **Figure S5.** Thelyphonida: Thelyphonidae: Hypoctoninae: *Yekuana venezolensis*, diploid set (A) and Mastigoproctinae: *Uroproctus assamensis*, haploid set (B), male karyotypes. In the haploid set, each chromosome pair is represented by one chromosome. Based on two sister metaphases II (A) or single metaphase II (B), stained by Giemsa. Scale bars = 10 μm. **Figure S6.** Thelyphonida: Thelyphonidae: Thelyphoninae: *Ginosigma* sp. (A) and *Thelyphonus* cf. *linganus* (B), male karyotypes. Based on two sister metaphases II (Giemsa staining). Scale bars = 10 μm. **Figure S7.** Ricinulei: *Cryptocellus narino* (A,C,D) and *Ricinoides olounoua* (B), male, pattern of NORs (red) and meiosis. (A) Pachytene, visualization of NORs (FISH, DAPI staining). One bivalent with subterminal NOR locus (arrowhead). (B) Mitotic metaphase, visualization of NORs (FISH, DAPI staining). Note small pair with subterminal NOR locus (arrowheads). (C) Diffuse stage (Giemsa staining). Bivalents exhibit enormous decondensation. (D) Metaphase I (Giemsa staining). Plate did not contain any heteromorphic bivalent. Note precocious segregation of chromosomes (arrows) of bivalent. Scale bars = 10 μm. **Figure S8.** Solifugae: females, association of homologous chromosomes throughout cell cycle (Giemsa staining). Letter pairs indicate putative homologs. (A) *Gluvia dorsalis*, early mitotic metaphase. (B) *Paragaleodes pallidus*, interphase nucleus containing haploid number (i.e., six) of blocks of CH. Scale bars = 10 μm. **Figure S9.** Solifugae: Eremobatidae (A) and Daesiidae (B), male karyotypes. Based on mitotic metaphase (Giemsa staining). (A) *Eremobates pallipes* karyotype with predominance of acrocentric pairs. Insets: NOR-bearing chromosomes from another mitotic metaphase (FISH, DAPI staining). Most of arm of metacentric pair no. 6 formed by NOR locus (red). (B) *Gnosippus* sp. karyotype with predominance of acrocentric chromosomes. Scale bars = 10 μm. **Table S1.** Material examined, including sex, instar, localities, and methods used. Abbreviations: C, C-banding; CGH, detection of sex chromosomes through CGH; F, fluorescent banding; fj, female juvenile; mj, male juvenile; j, juvenile, sex undetermined; NOR, NOR detection through FISH; S, standard evaluation of mitosis and meiosis (Giemsa-stained preparations); subad, subadult; T, telomeric repeat detection through FISH.
