# Supplementary material for: Advances in Understanding the Karyotype Evolution of Tetrapulmonata and Two Other Arachnid Taxa, Ricinulei and Solifugae
Source: Genes (Basel). 2025 Feb 8;16(2):207. doi: 10.3390/genes16020207 (PMC11855311; doi:10.3390/genes16020207)
Supplement: Supplementary file 1 [file genes-16-00207-s001.zip › Table_S1.pdf]

Species	Methods used	Locality/origin	Specimens studied
**Amblypygi**			
Charontidae			
Charon cf. *grayi* (Gervais, 1842)	S, NOR, CGH	Mambucal-Kanlaon volcano, island Negros, Philippines	4♂, 2♀
Phrynichidae			
Damon medius (Herbst, 1797)	S, NOR	breeding (Togo)	1♂
Euphrynichus amanica (Werner, 1916)	S, NOR	breeding (Tanzania)	1♂
E. bacillifer (Gerstaecker,1873)	S, NOR	breeding (Tanzania)	1♂
Phrynichus ceylonicus (C.L. Koch, 1843)	S, NOR	Sigiriya, Sri Lanka	3♂, 1♀
Phrynidae			
Acanthophrynus coronatus (Butler, 1873)	S, C, NOR, T	Cerro de los Túneles, Anenecuilco municipality, Cuautla State, Mexico	1♂
Heterophrynus cf. *elaphus* Pocock, 1903	S, NOR	Manu Biosphere Reserve, Peru	1♂
Paraphrynus mexicanus (Bilimek, 1867)	S, NOR, CGH	cave Juxtlahuaca, Colotlipa, Guerrero State, Mexico	2♂, 4♀
**Thelyphonida**			
Thelyphonidae (Hypoctoninae)			
Hypoctonus cf. *gastrostictus* Kraepelin, 1897	S, NOR	breeding (Malaysia)	2♂
Labochirus proboscideus (Butler, 1872)	S, C, F	Palolem env., 30 km S of Madgaon, Goa province, India	2♂, 1subad♂, 2♀
Yekuana venezolensis (Haupt, 2009)	S, NOR	88 km road to Guiana Upland from San Isidro, Bolivar State, Venezuela	1♂
Thelyphonidae (Mastigoproctinae)			
Mastigoproctus giganteus (Lucas, 1835)	S, C, NOR	breeding (USA)	1♂, 1♀
Uroproctus assamensis (Stoliczka, 1869)	S, NOR	Nongpoh, Meghalaya State, India	1mj, 1♀
Thelyphonidae (Thelyphoninae)			
Ginosigma sp.	S, C, NOR, T	Gunung Jasar, Cameron Highlands, Tanah Rata, Malaysia	1subad♂
Thelyphonus cf. *linganus* C.L.Koch, 1843	S, F	Genting, island Tioman, Malaysia	2♂, 1♀
Thelyphonidae (Typopeltinae)			
Typopeltis crucifer Pocock, 1894		breeding (Taiwan)	1♀
T. guangxiensis Haupt and Song, 1996	S, C, NOR, CGH	breeding (Guangxi Province, China)	2♂, 1♀

Species	Methods used	Locality/origin	Specimens studied
**Schizomida**			
Hubbardiidae			
Clavizomus sp.	S	Singapore	1♂
Notozomus sp.	S	Cape Tribulation, Queensland, Australia	1♂
Orientzomus sp. (Luzon)	S	botanical garden, Los Baños, Laguna, Luzon, Philippines	1 subad♂, 1♀
Orientzomus sp. (Mindanao)	S, NOR	Mt. Malambo, San Fernando, Mindanao, Philippines	1♀
		Mt. Musuan, Central Mindanao University, Mindanao, Philippines	1♀
Stenochrus sp.	S, T	surroundings of Laguna Escondida, S of Montepío, Los Tuxtlas Mts., Veracruz State, Mexico	2♂, 1♀
Olmecazomus brujo Monjaraz-Ruedas and Francke, 2017	S, NOR	tropical forest, Playa Escondida, SE of Montepío, Los Tuxtlas Mts., Veracruz State, Mexico	2♂, 2♀
		tropical forest, field station of UNAM, S of Montepío, Los Tuxtlas Mts., Veracruz State, Mexico	3♂, 1mj, 3fj
		surroundings of Laguna Escondida, S of Montepío, Los Tuxtlas Mts., Veracruz State, Mexico	2♀, 2fj
Hubbardiidae sp. (Seychelles)	S, NOR	Mahé Island, Republic of Seychelles	2♀, 1j
Hubbardiidae sp. (Cameroon)	S, NOR	Mt. Coupe, N of Loume, Cameroon	2♀, 1j
Protoschizomidae			
Agastoschizomus lucifer Rowland, 1971	S, NOR	Sótano de Yerbaniz cave, municipality Ciudad Valles, San Luis Potosí State, Mexico	10♀
**Ricinulei**			
Cryptocellus narino Platnick and Paz, 1979	S, NOR	122 km from Medellín on highway to Nariño, Colombia	2♂, 1♀
Pseudocellus gertschi (Márquez and Conconi, 1974)	S, NOR, T	tropical forest, Playa Escondida, SE of Montepío, Los Tuxtlas Mts., Veracruz State, Mexico	2♂, 2♀
Ricinoides olounoua	S, NOR	Ebogo near Mbalmayo, Cameroon	2♂
**Solifugae**			
Ammotrechidae			
Ammotrechula mulaiki Muma, 1951	S	Anenecuilco, Cuautla State, Mexico	1mj, 1fj
Daesiidae			
Eberlanzia flava Roewer, 1941	S, NOR	breeding (Namibia)	1mj
Gluvia dorsalis (Latreille, 1817)	S, C	Valverde da Mitra, Evora, Alentejo, Portugal	1mj, 3fj
Gnosippus sp.	S	Faliraki, Rhodos, Greece	1mj

Species	Methods used	Locality/origin	Specimens studied
Eremobatidae			
Eremobates pallipes (Say, 1823)	S, NOR	Comanche National Grassland, Colorado, USA	1mj
E. similis Muma, 1951	S, NOR	Moffat, Saguache County, Colorado, USA	1mj
Galeodidae			
Paragaleodes pallidus (Birula, 1890)	S, C, NOR	right river side of Ili river at Kapchagay canyon, Itzhon Plateau, 10 km N of Kapchagay, Kazakhstan	1subad♂, 2mj, 1subad♀
Rhagodidae			
Rhagodes sp.	S, C	Mashabbim sands, Negev Desert, Israel	5 postembryos
Solpugidae			
Solpugista sp.	S, NOR	Erfenis Dam, Free State, South Africa	1fj
