# Supplementary figures and images for: Advances in Understanding the Karyotype Evolution of Tetrapulmonata and Two Other Arachnid Taxa, Ricinulei and Solifugae
Source: Genes (Basel). 2025 Feb 8;16(2):207. doi: 10.3390/genes16020207 (PMC11855311; doi:10.3390/genes16020207)

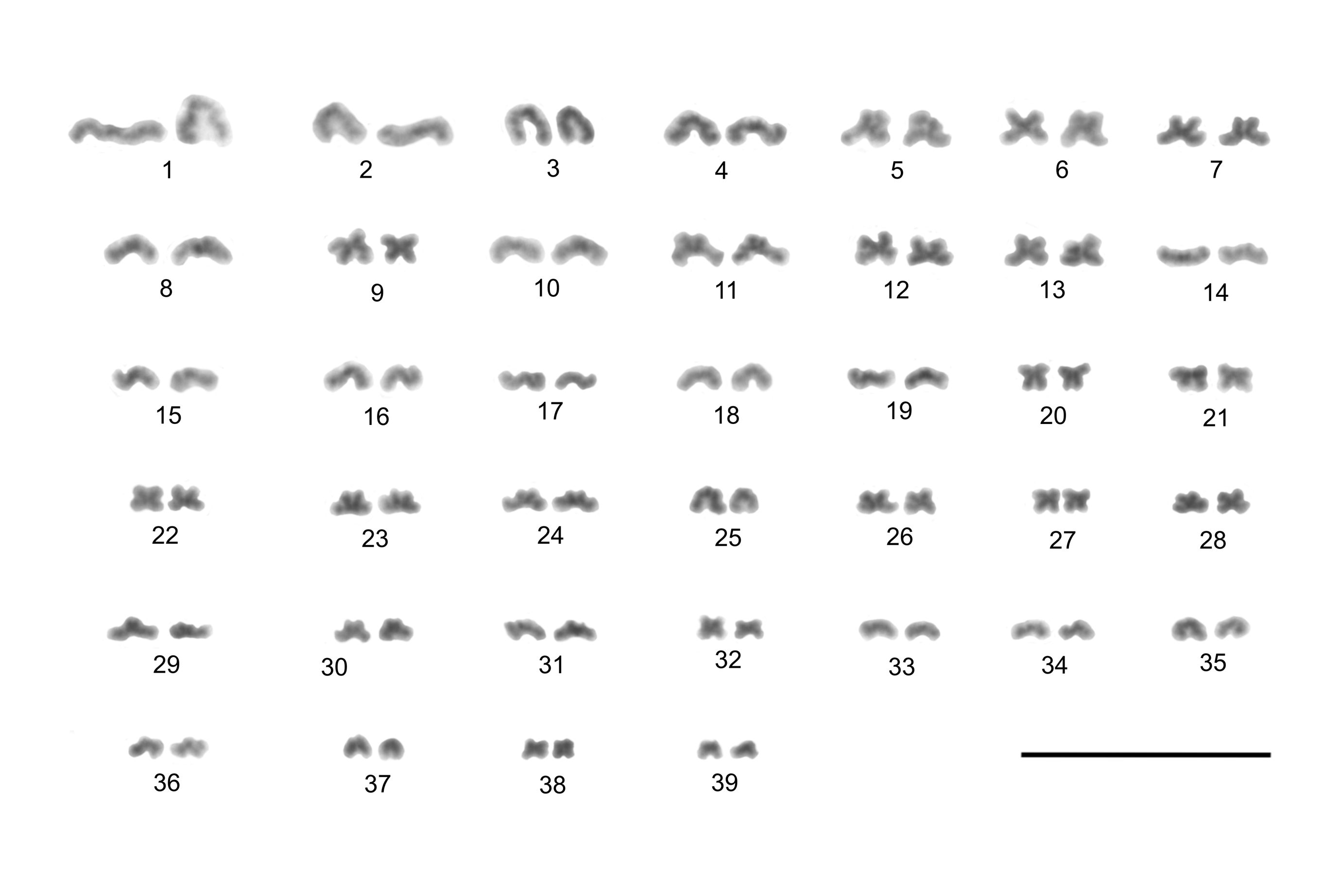

Supplement: Supplementary file 1 [file genes-16-00207-s001.zip › fig S1.tif]

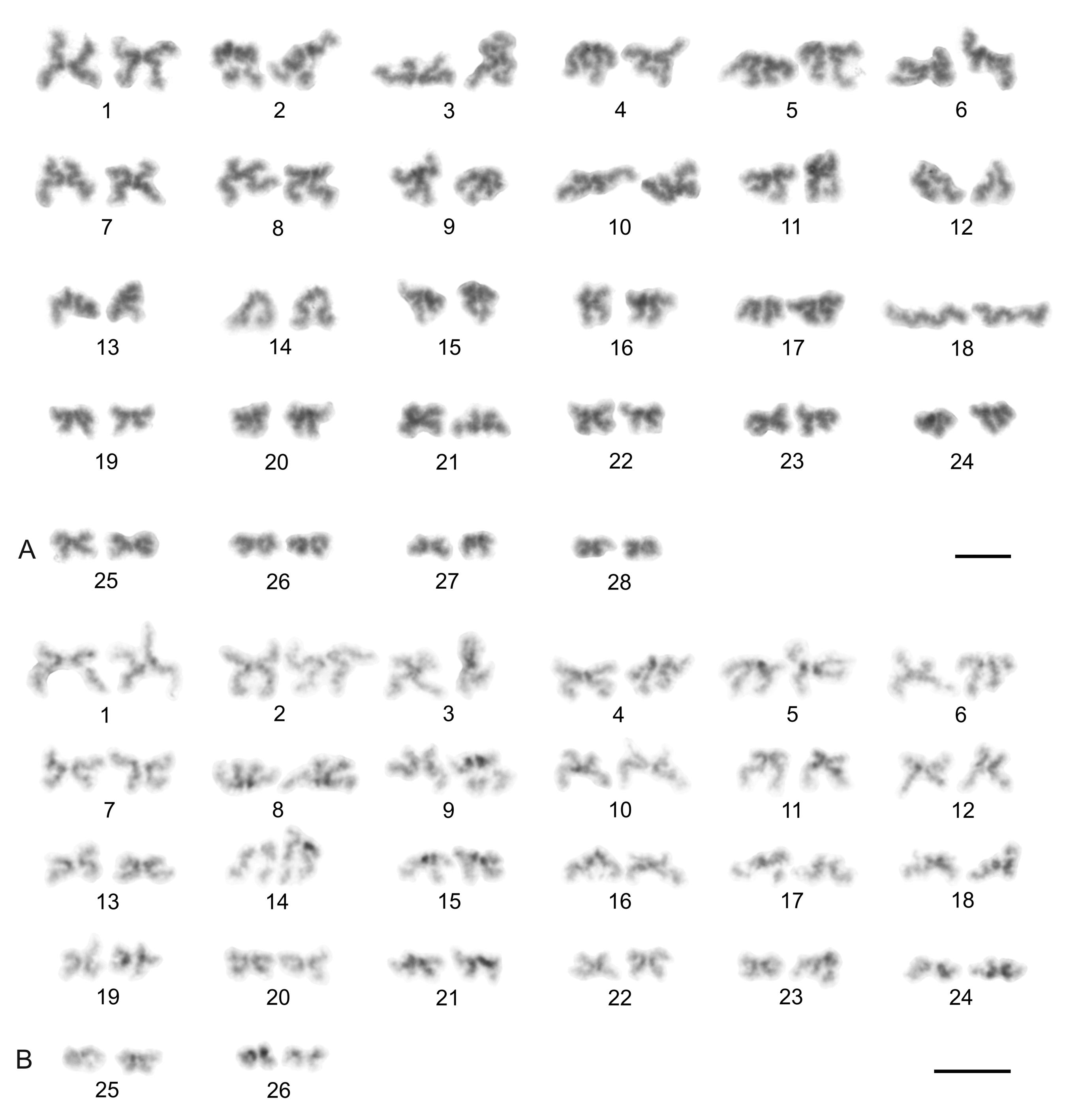

Supplement: Supplementary file 1 [file genes-16-00207-s001.zip › fig S2.tif]

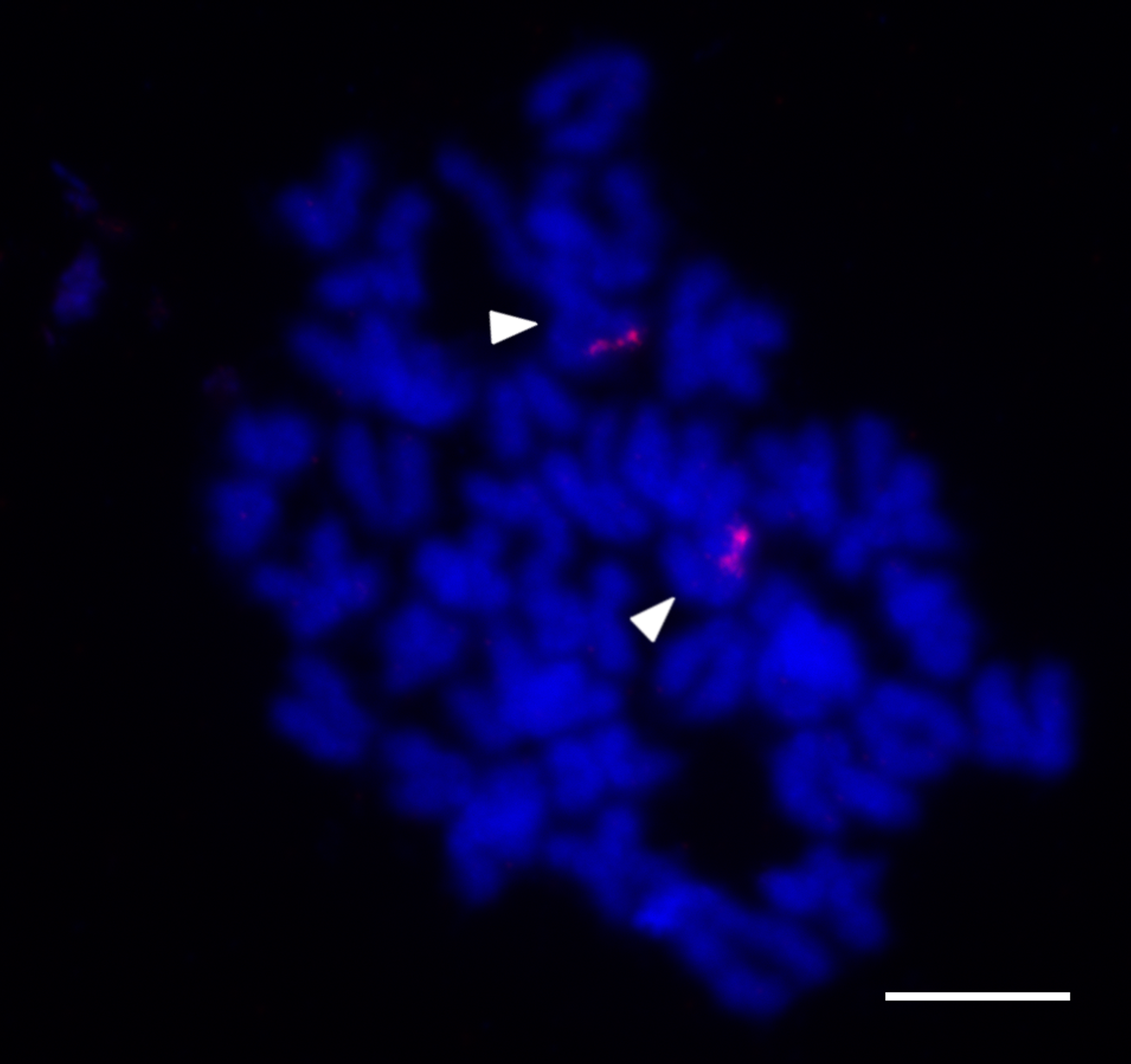

Supplement: Supplementary file 1 [file genes-16-00207-s001.zip › fig S3.tif]

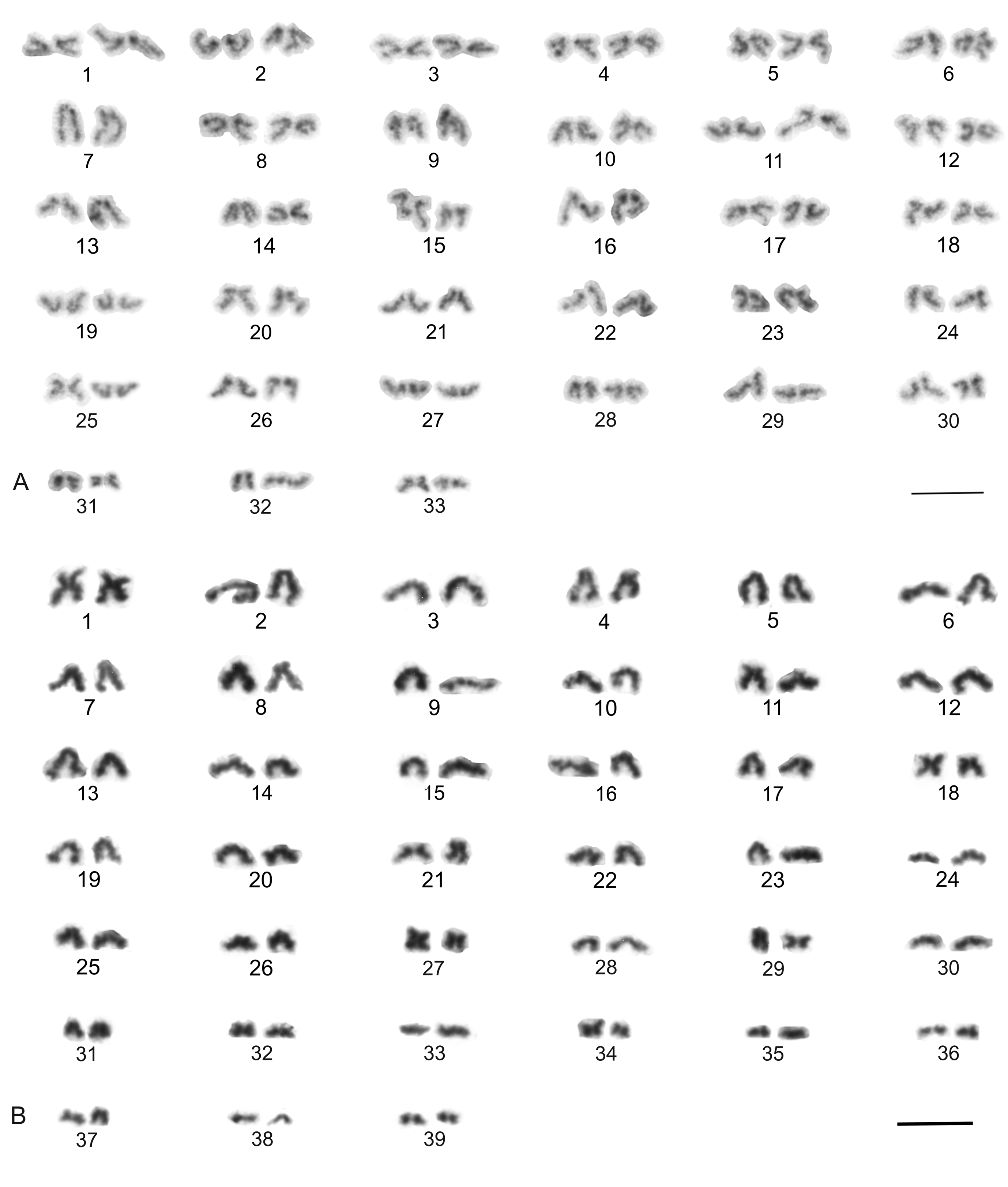

Supplement: Supplementary file 1 [file genes-16-00207-s001.zip › fig S4.tif]

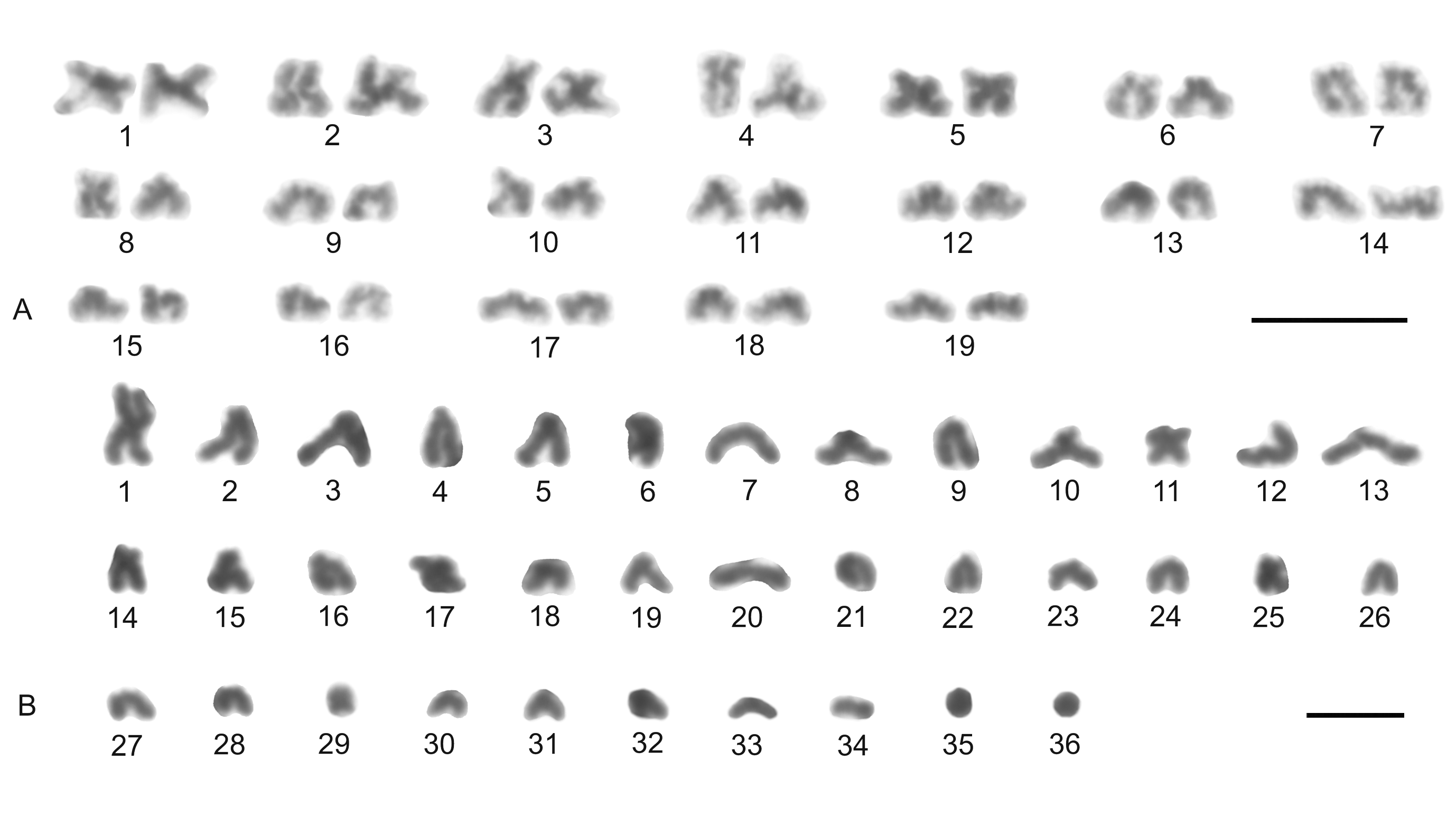

Supplement: Supplementary file 1 [file genes-16-00207-s001.zip › fig S5.tif]

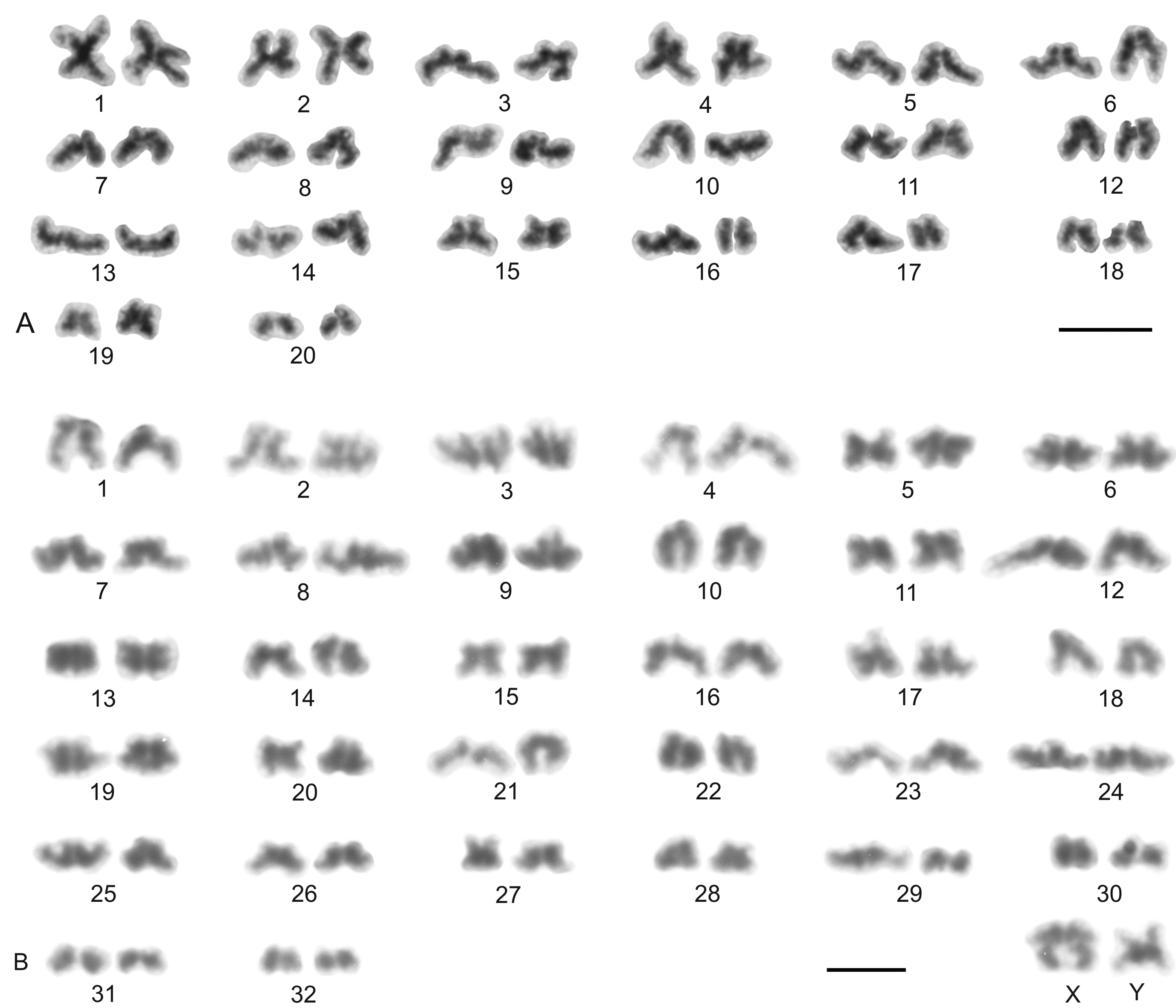

Supplement: Supplementary file 1 [file genes-16-00207-s001.zip › fig S6.tif]

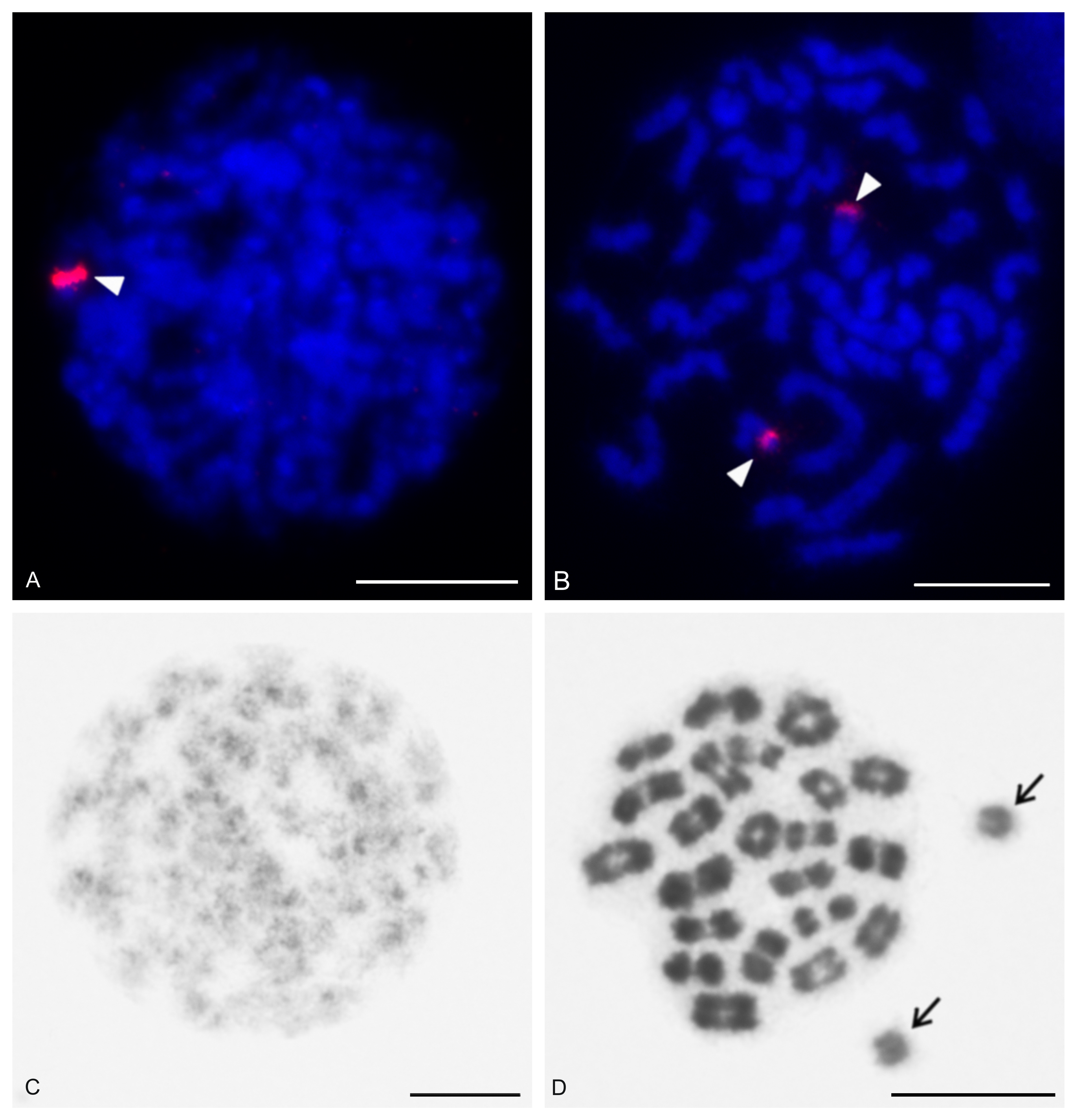

Supplement: Supplementary file 1 [file genes-16-00207-s001.zip › fig S7.tif]

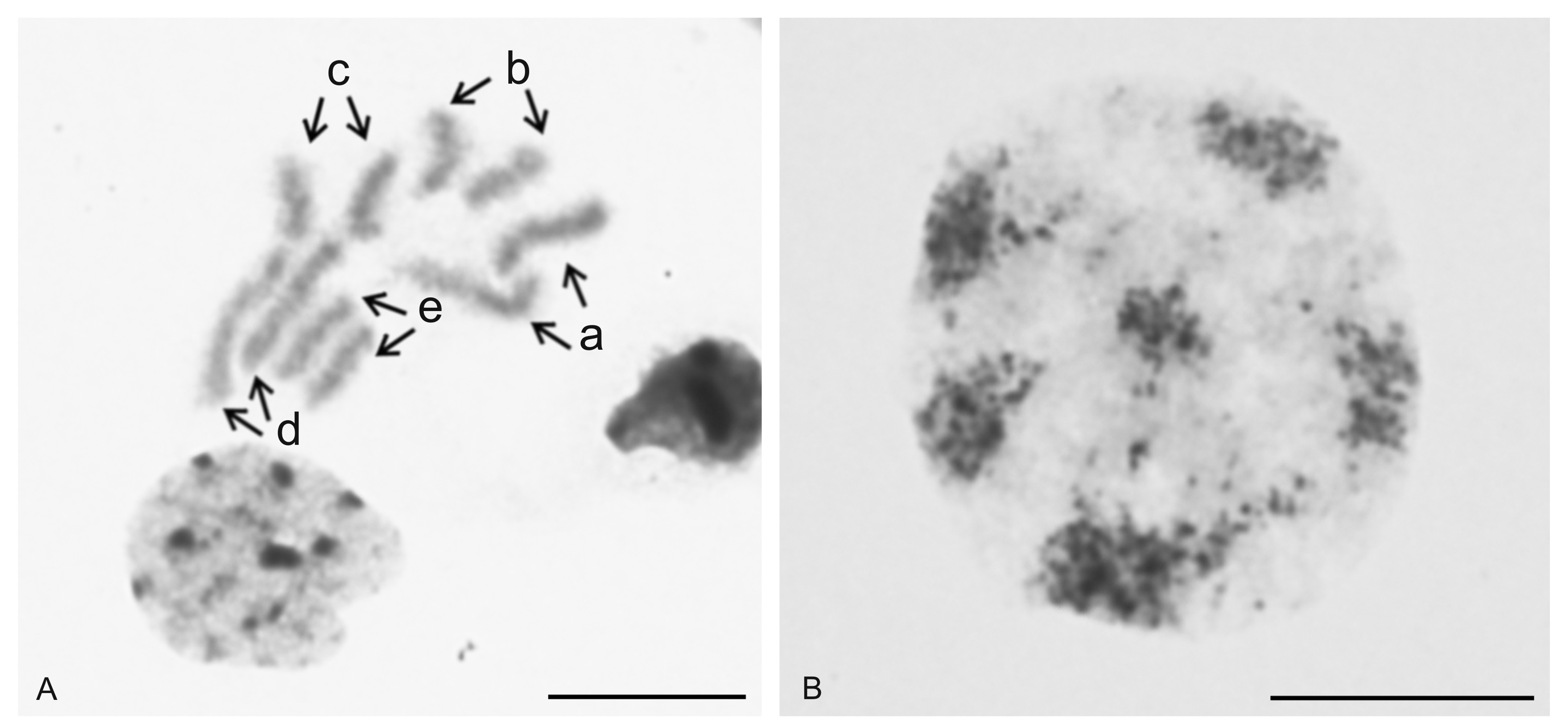

Supplement: Supplementary file 1 [file genes-16-00207-s001.zip › fig S8.tif]
